# Supplementary material for: Design and Synthesis of Novel Chalcone Derivatives: Anti-Breast Cancer Activity Evaluation and Docking Study
Source: Int J Mol Sci. 2023 Oct 25;24(21):15549. doi: 10.3390/ijms242115549 (PMC10649752; doi:10.3390/ijms242115549)
Supplement: Supplementary file 1 [file ijms-24-15549-s001.zip › ijms-2598057-supplementary.pdf]

Design and Synthesis of Novel Chalcone Derivatives: Anti-breast cancer Activity Evaluation and Docking Study

Weihong Lai [1], Jiaxin Chen [1], Xinjiao Gao [1], Xiaobao Jin [2], Gong Chen[1,*], Lianbao Ye[1,*]

[1] School of Pharmacy, Guangdong Pharmaceutical University, Guangzhou 510006, China.

[2] Guangdong Key Laboratory of Pharmaceutical Bioactive Substances, Guangdong Pharmaceutical University, Guangzhou 510006, China.

* Correspondence：Gong Chen: gongchen@gdpu.edu.cn, Tel: +86-20-39352139;

Lianbao Ye: yelb7909@163.com,Tel: +86-20-39352128;

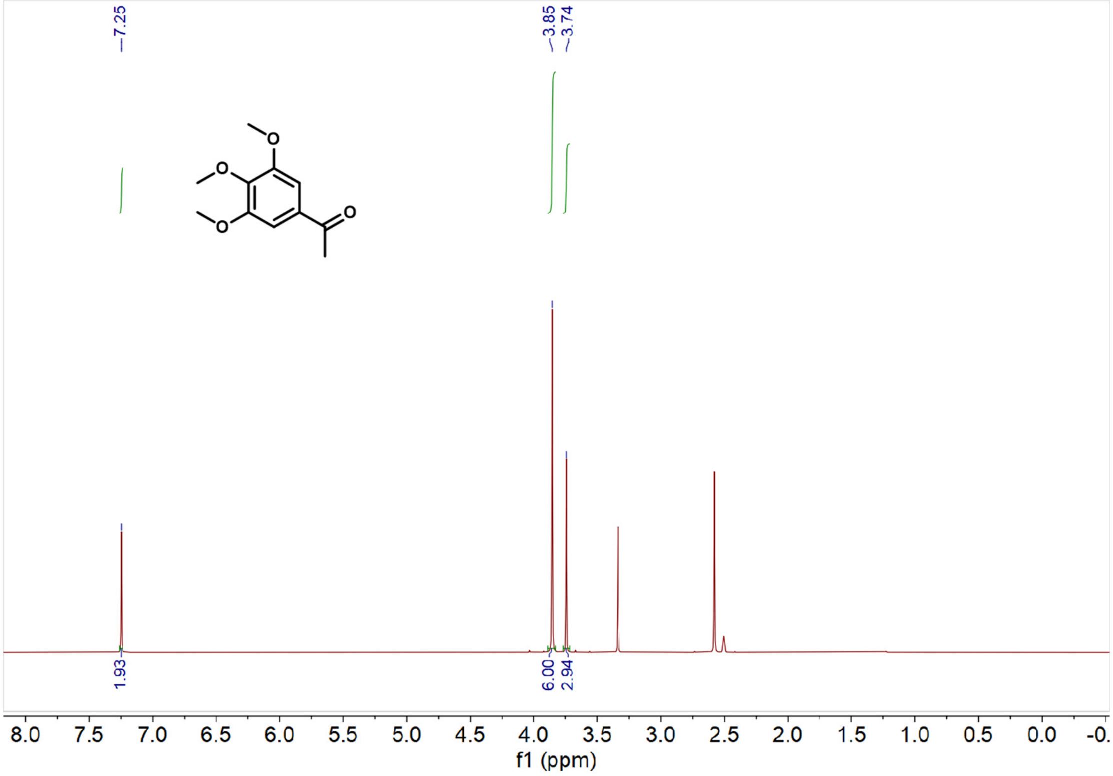

Figure S1^{1}H-NMR（**2a**）

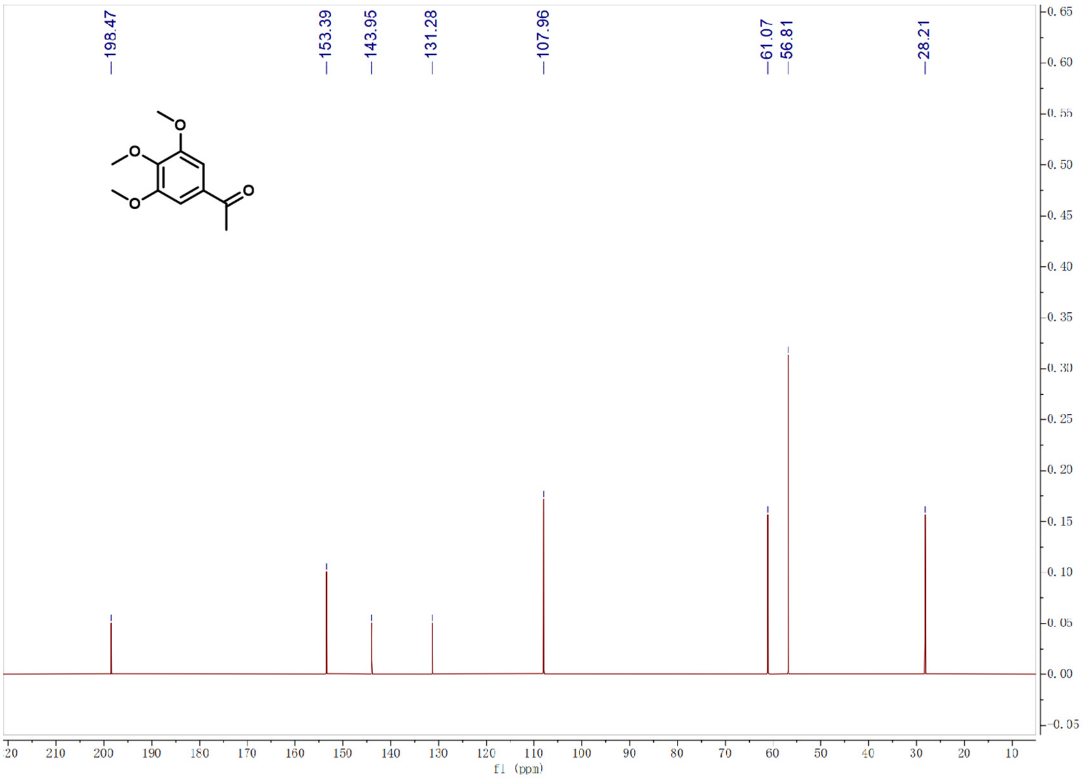

Figure S2^{13}C-NMR（**2a**）

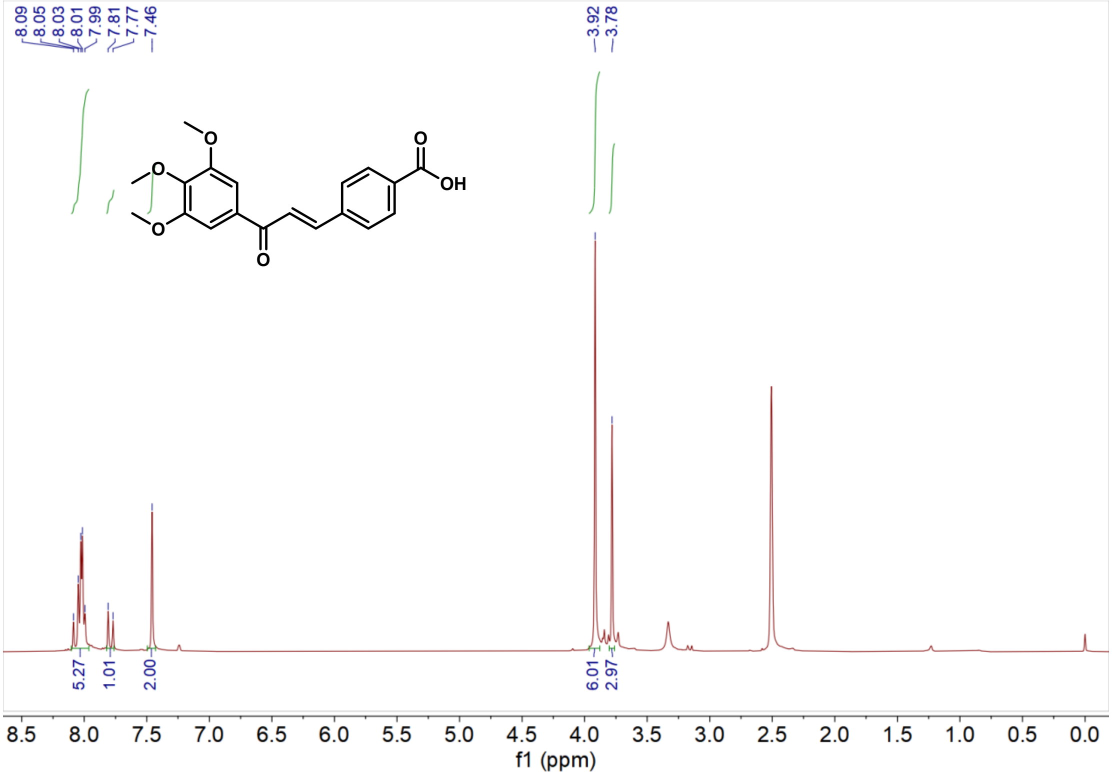

Figure S3 ^{1}H-NMR（**4a**）

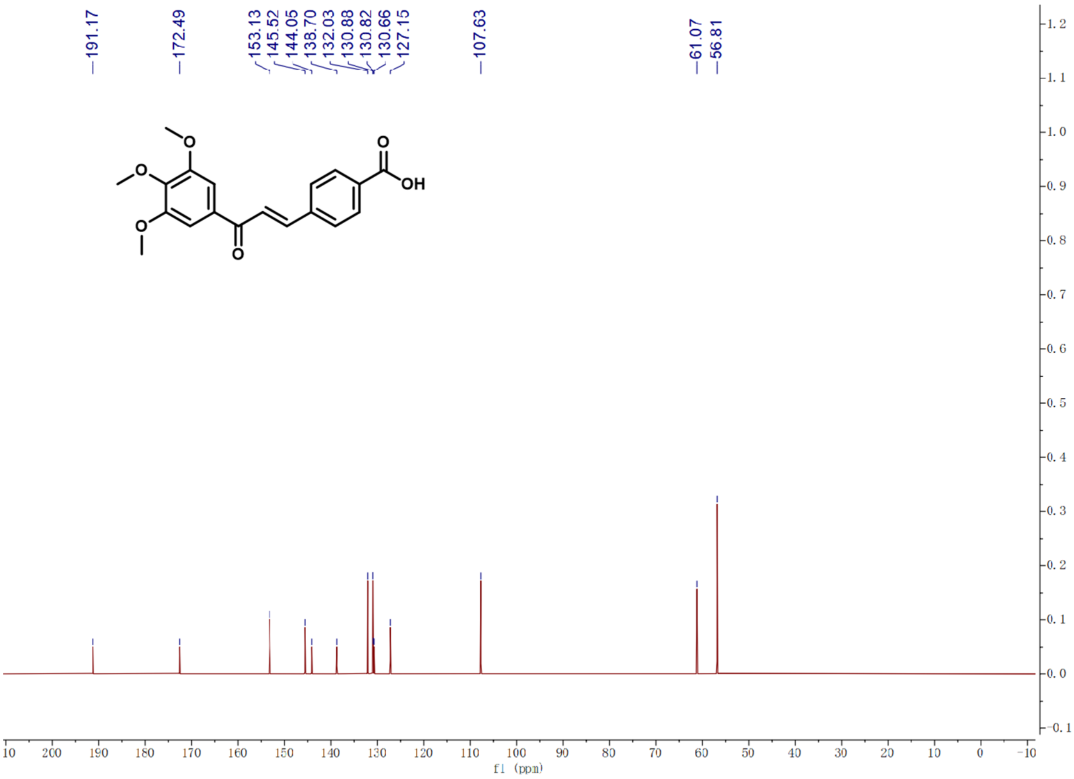

Figure S4 ^{13}C-NMR（**4a**）

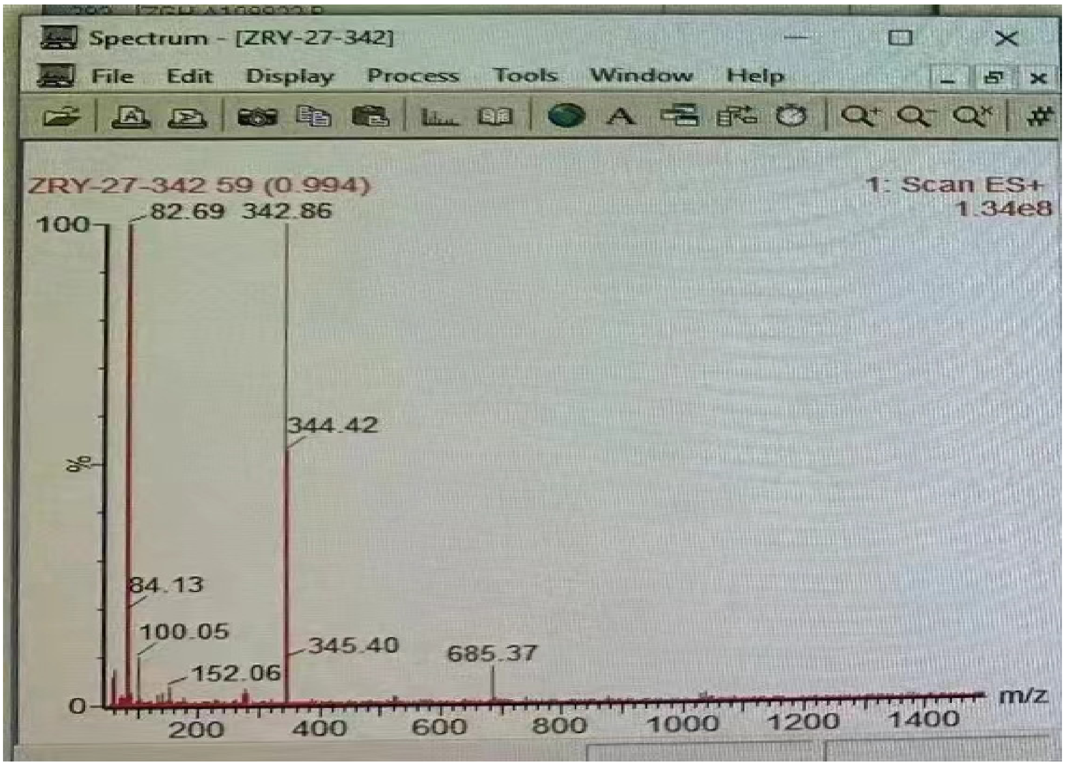

Figure S5 MS (4a)

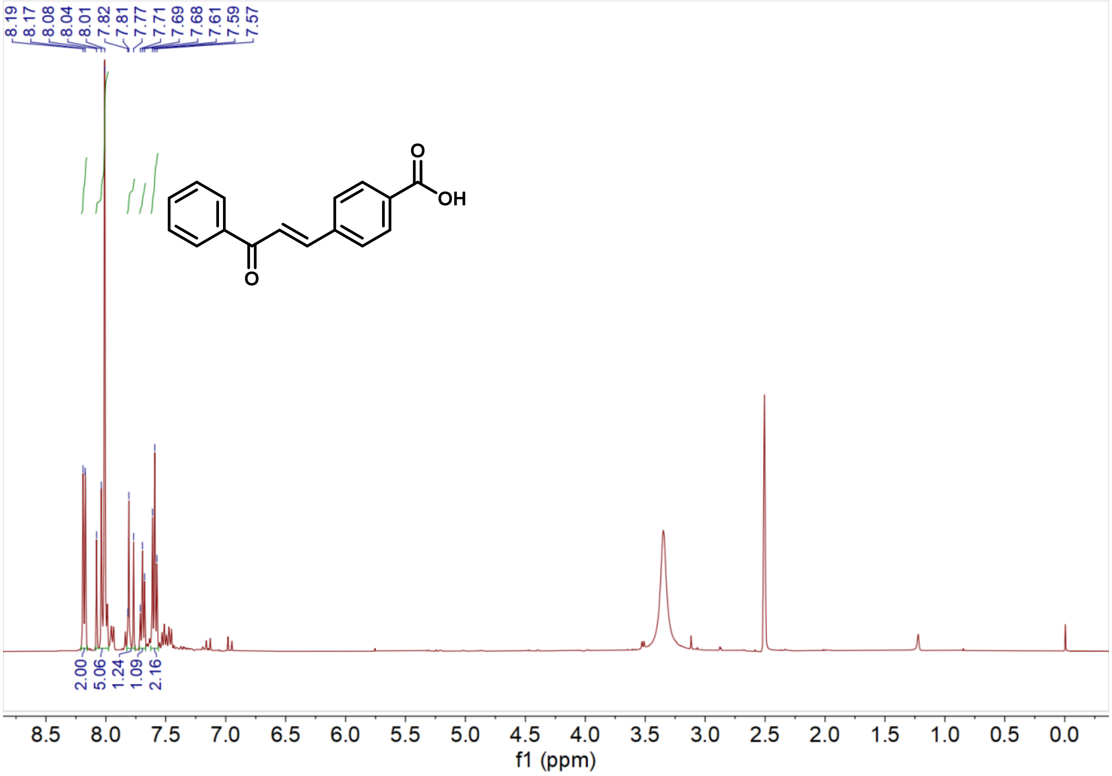

Figure S6 ^{1}H-NMR（**4b**）

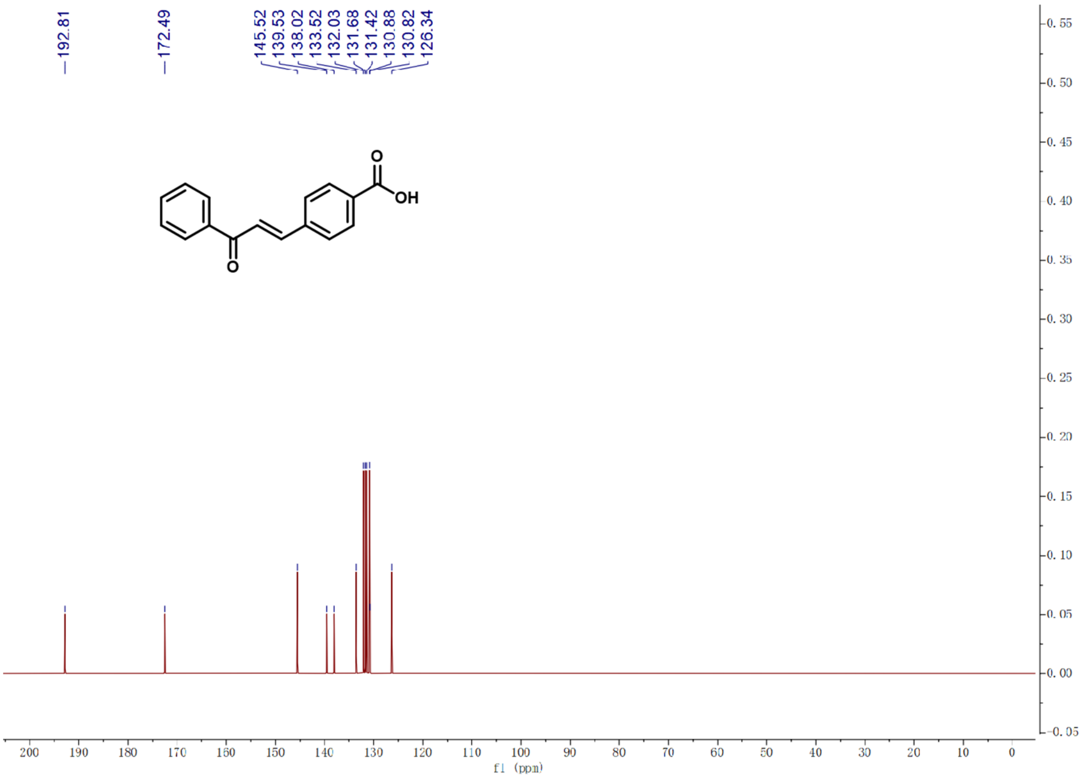

Figure S7 ^{13}C-NMR（**4b**）

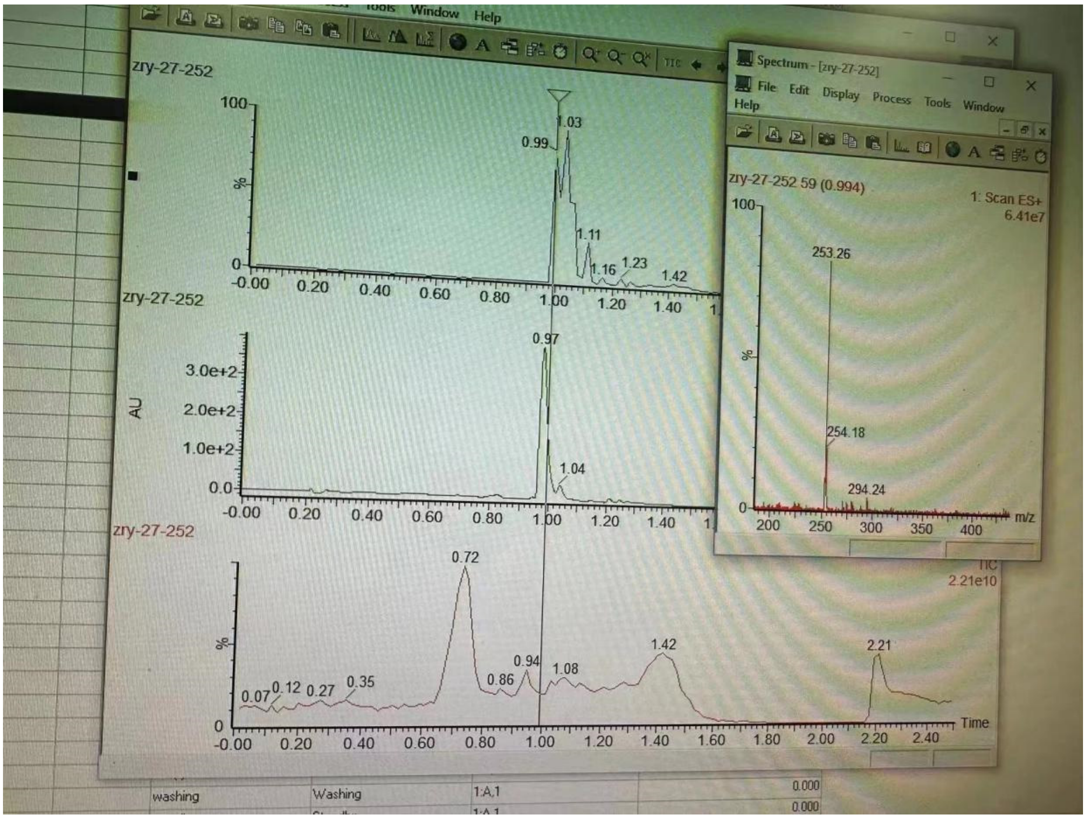

Figure S8 MS (**4b**)

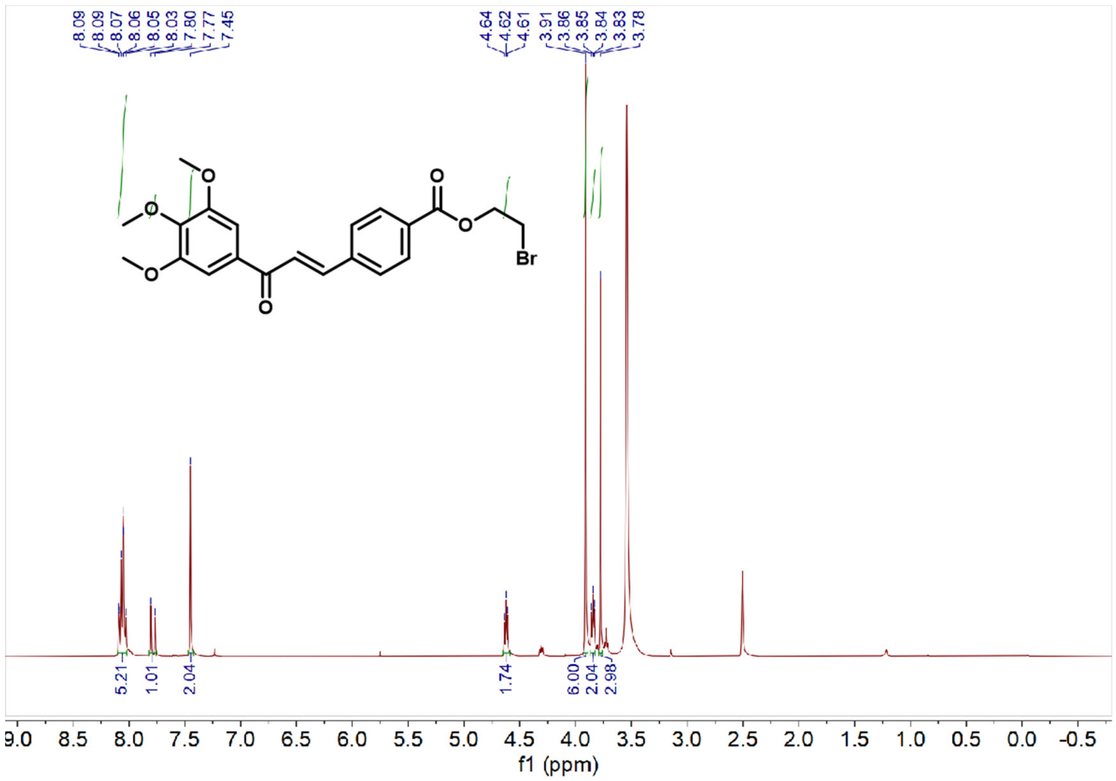

Figure S9 ^{1}H-NMR（**5b**）

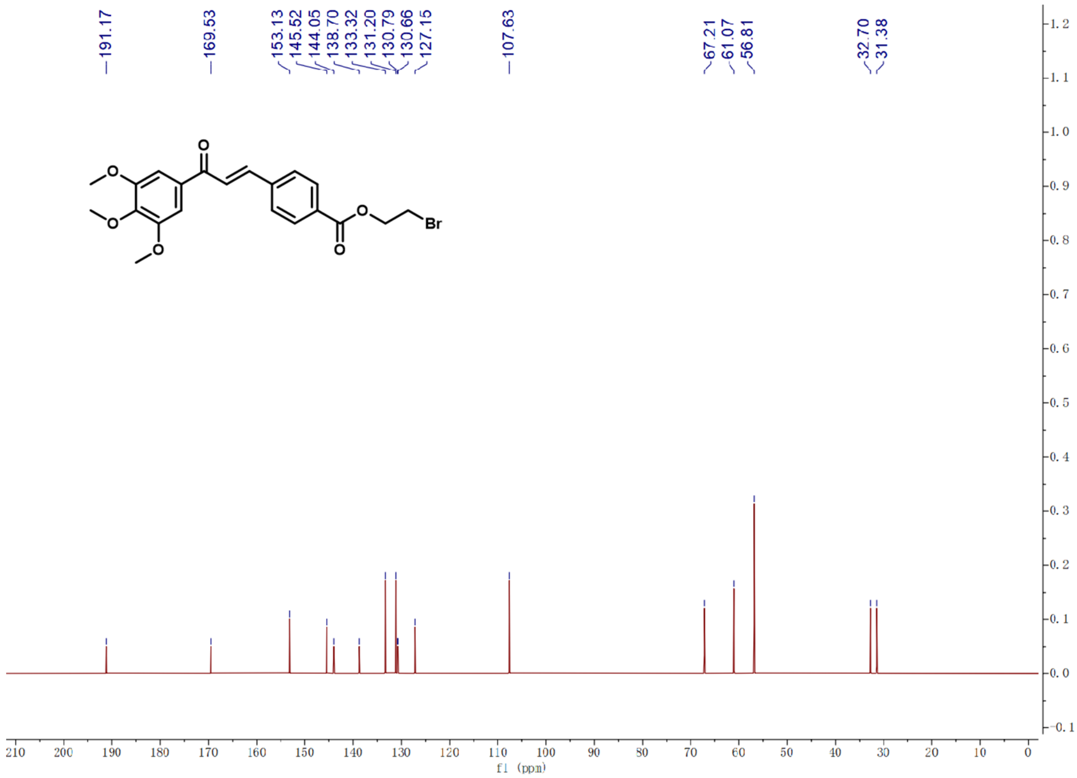

Figure S10 ^{13}C-NMR（**5b**）

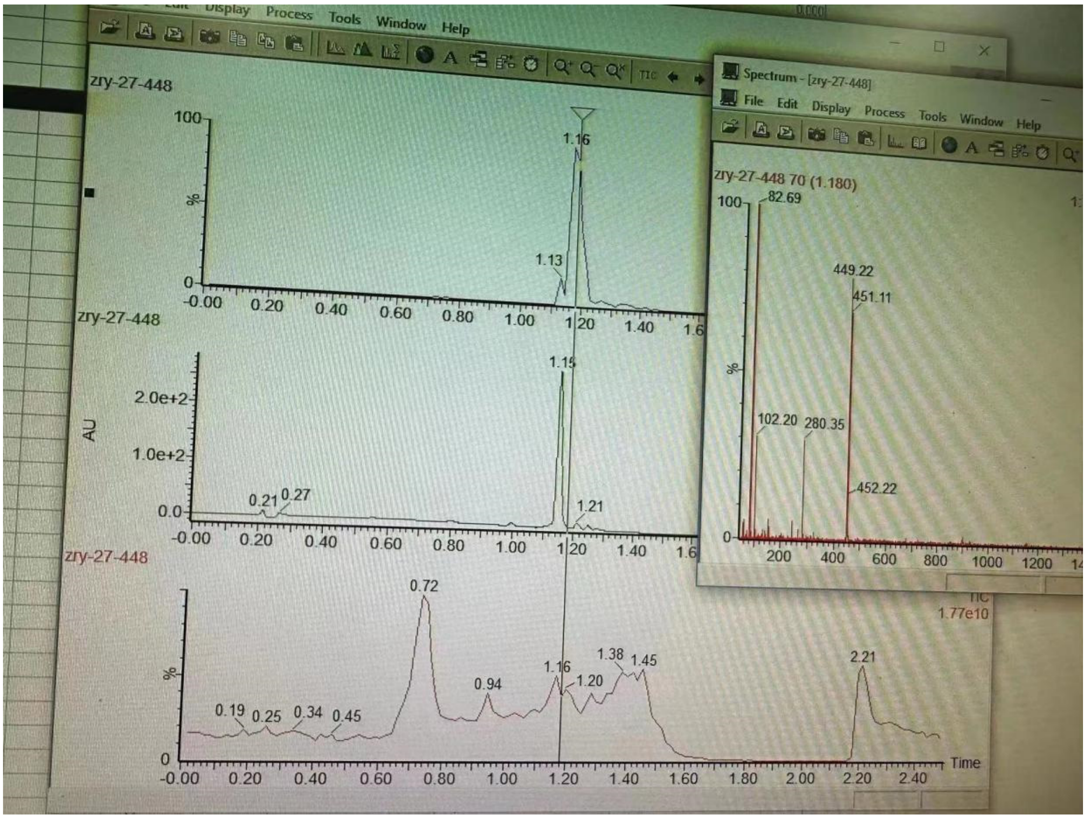

Figure S11 MS (**5b**)

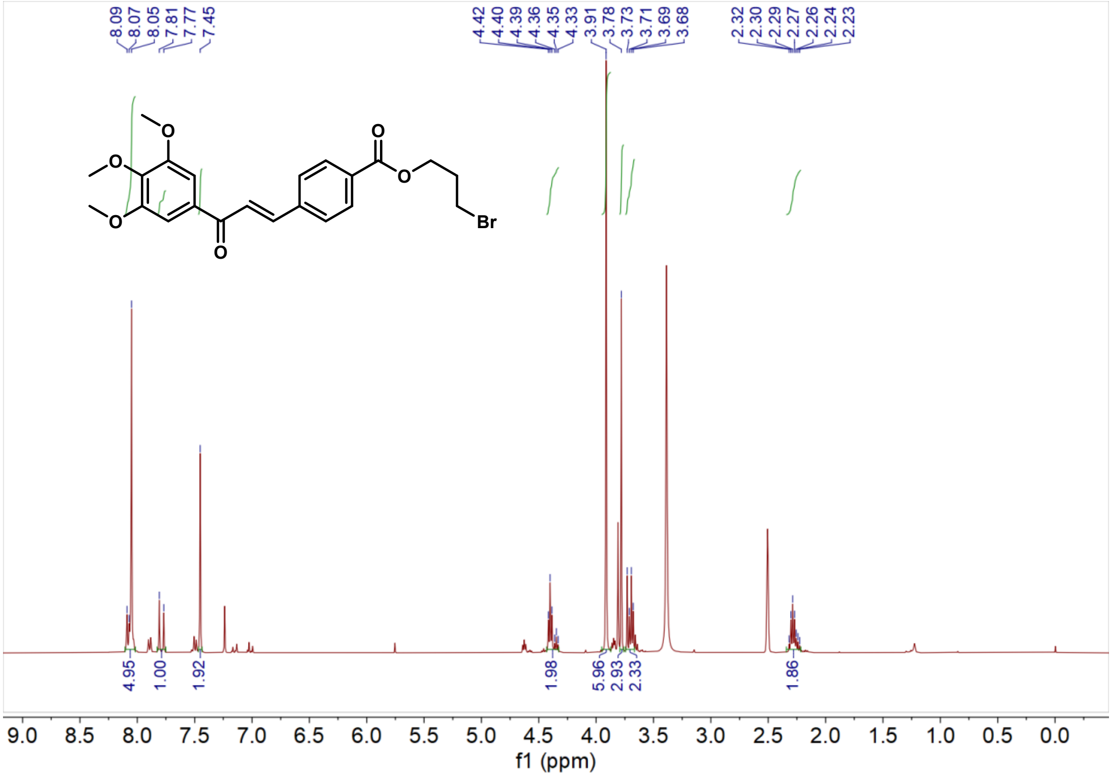

Figure S12 ¹H-NMR（**5c**）

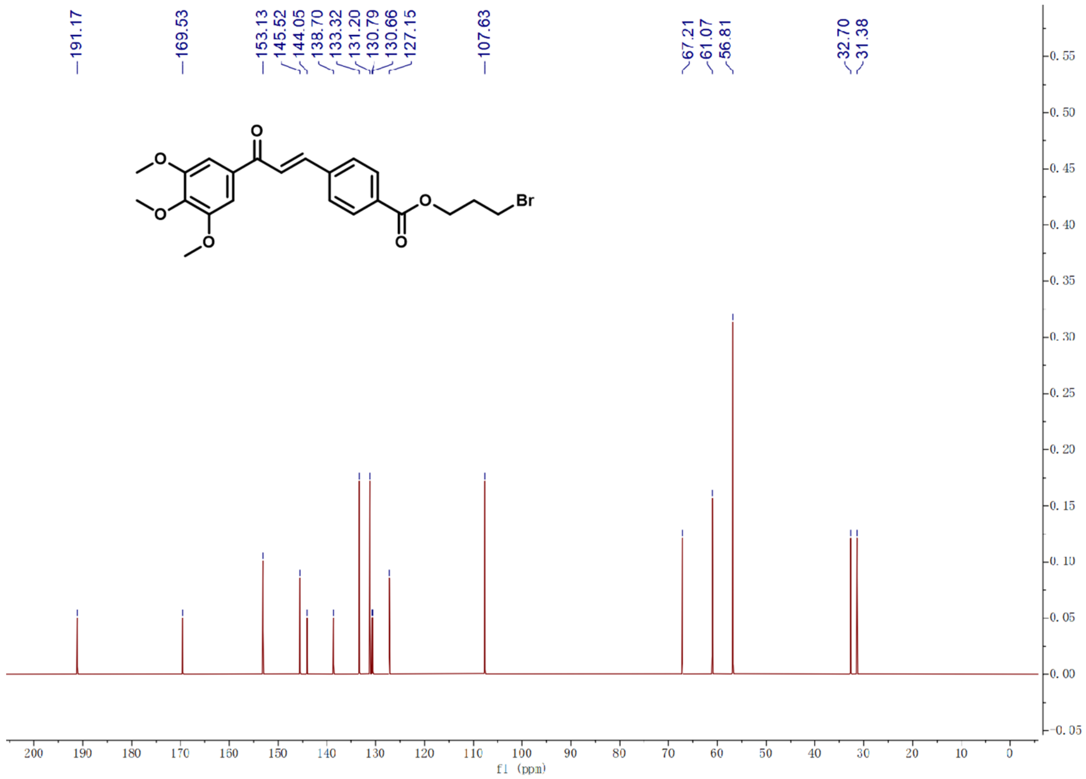

Figure S13 ^{13}C-NMR（**5c**）

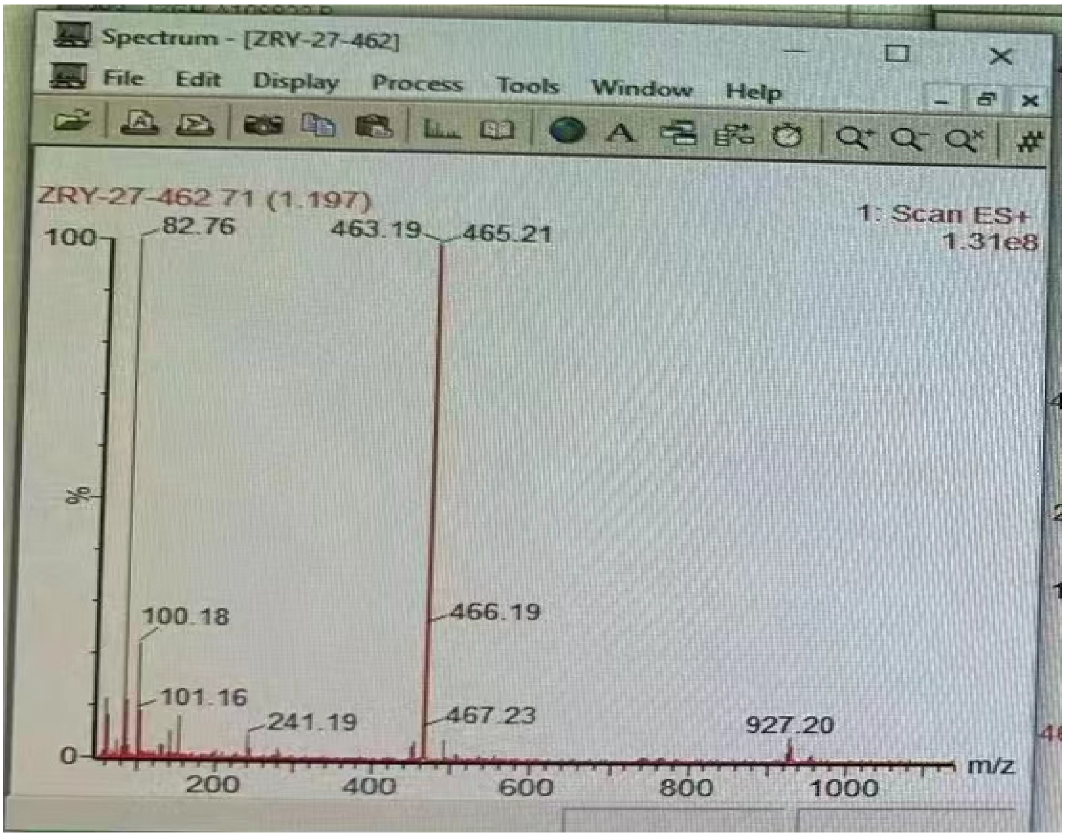

Figure S14 MS (**5c**)

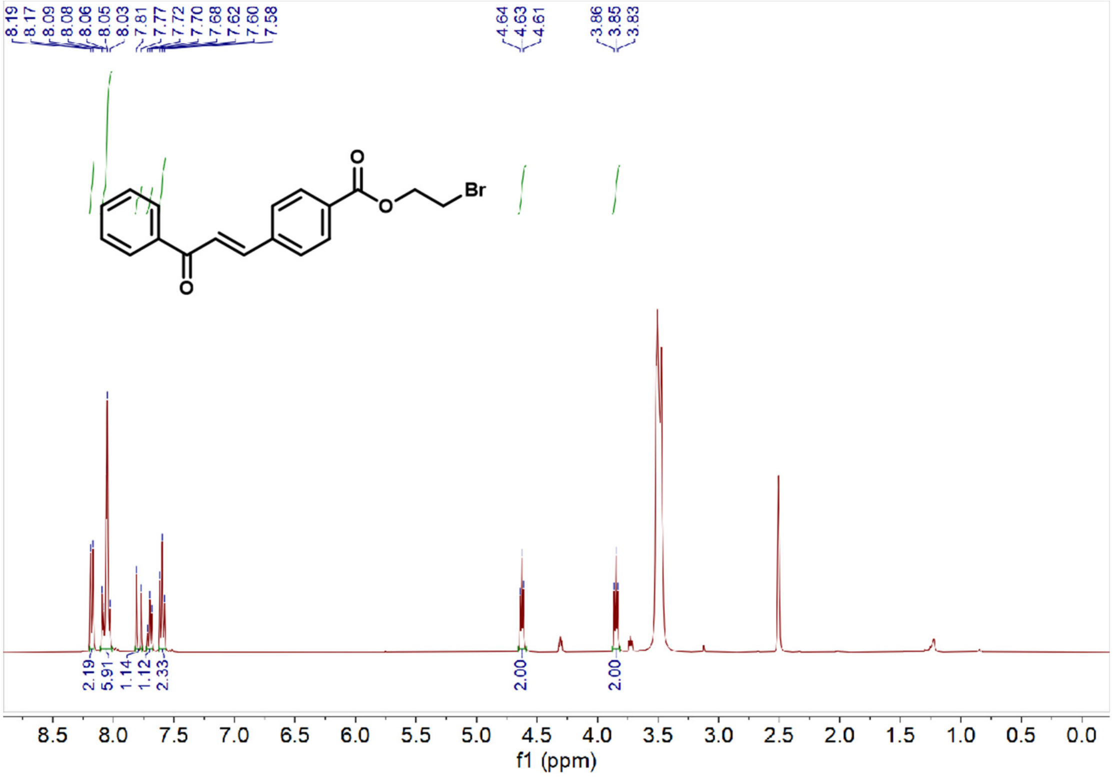

Figure S15 ^{1}H-NMR（**5e**）

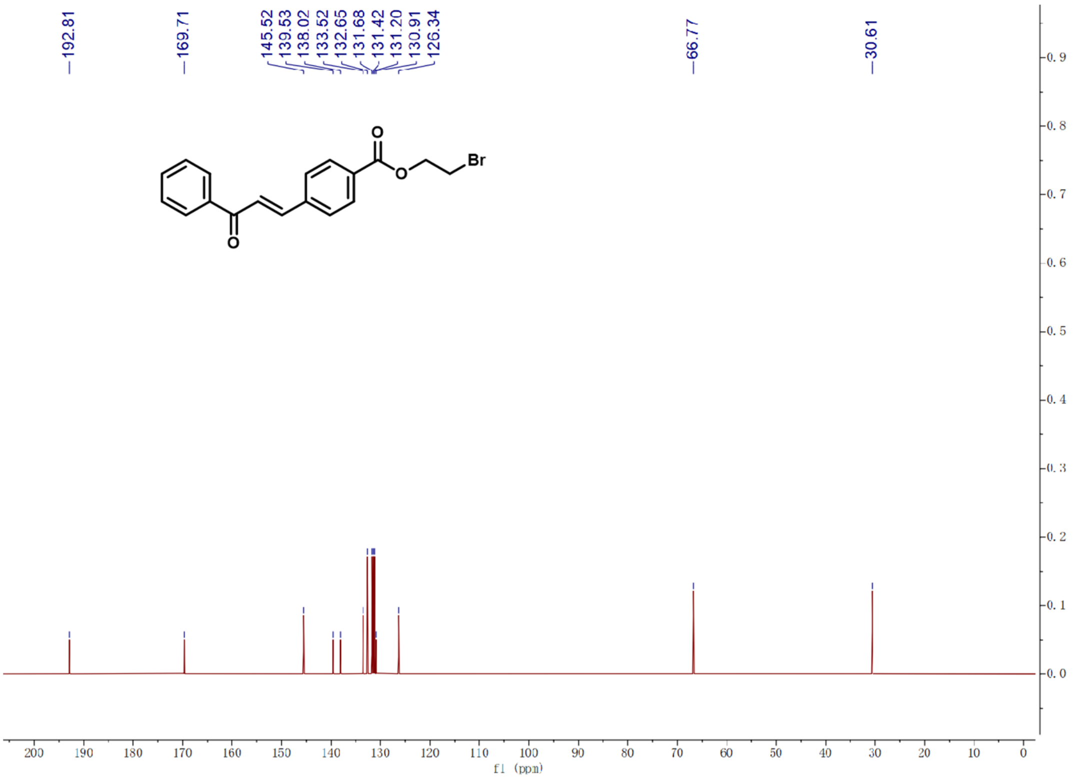

Figure S16 ^{13}C-NMR（**5e**）

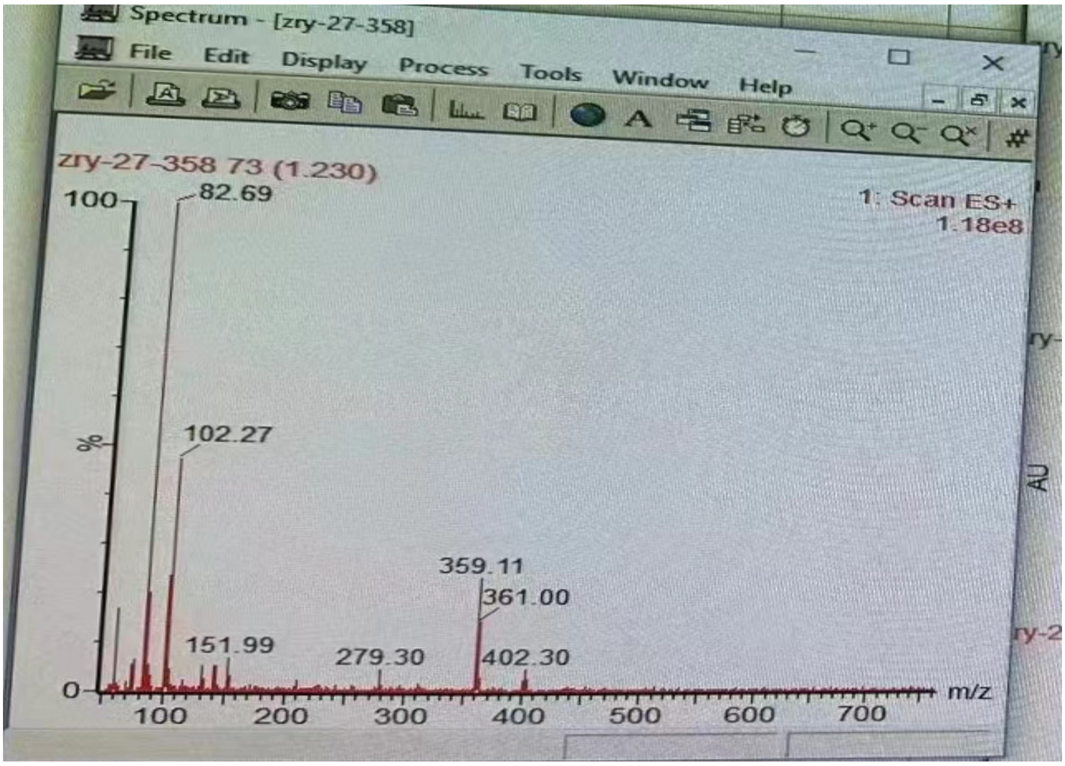

Figure S17 MS (**5e**)

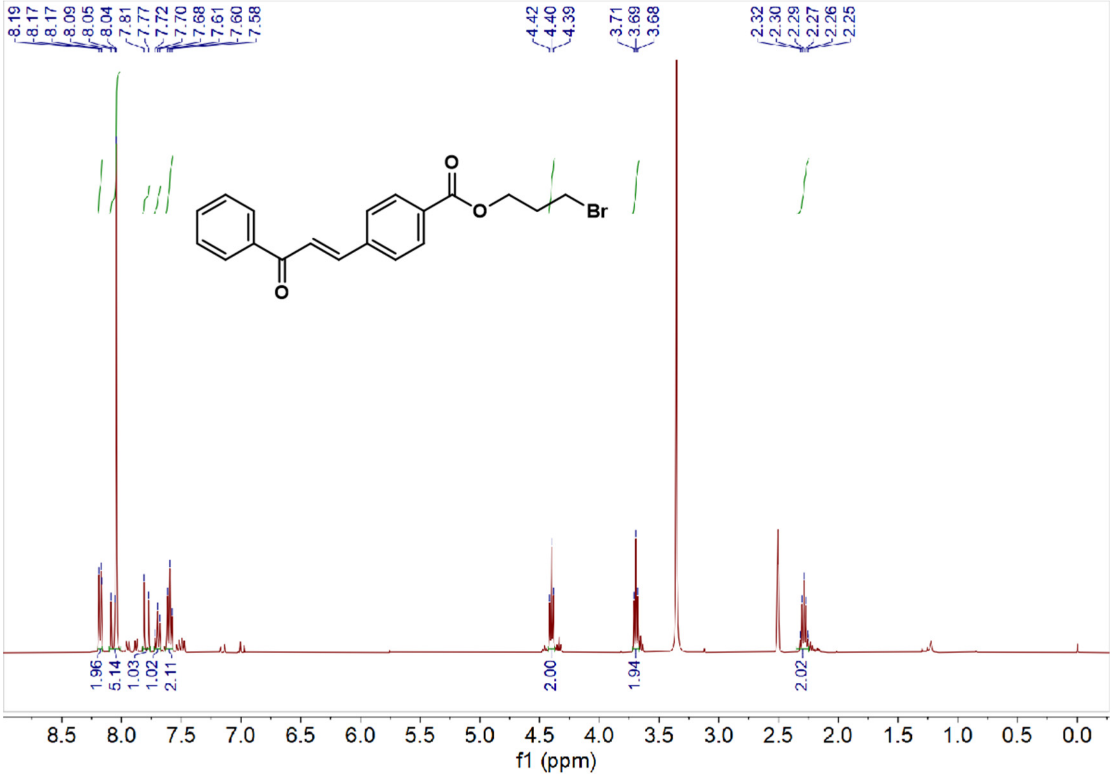

Figure S18 ^{1}H-NMR（**5f**）

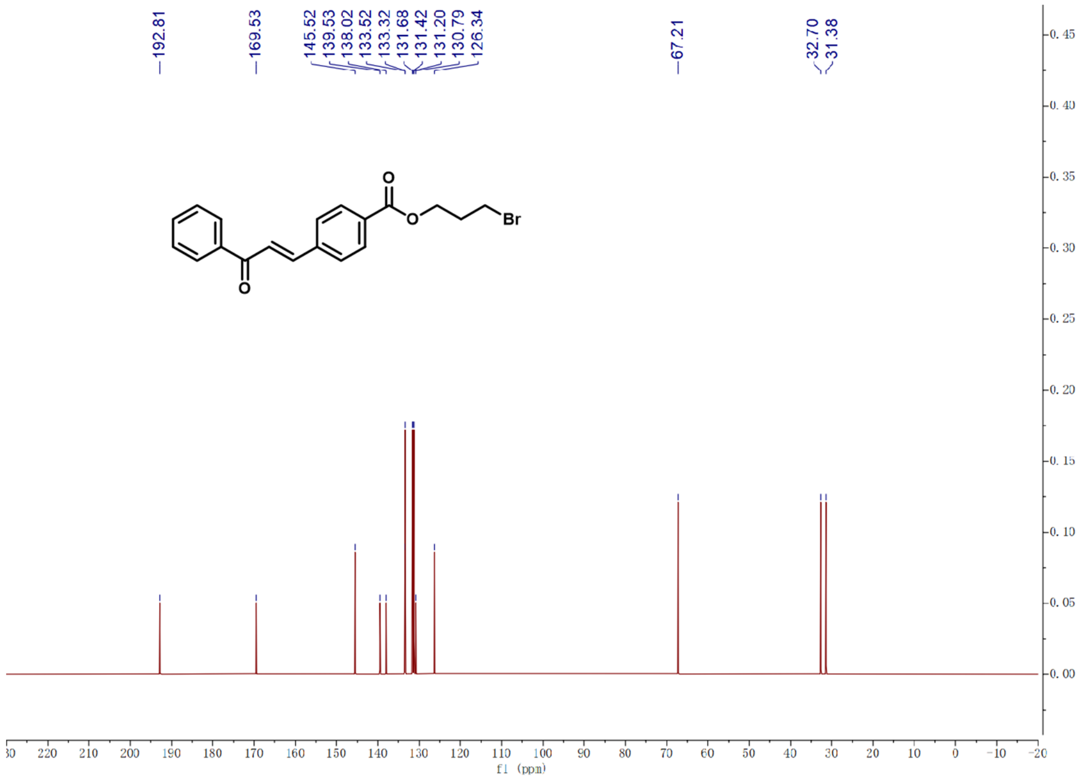

Figure S19 ^{13}C-NMR（**5f**）

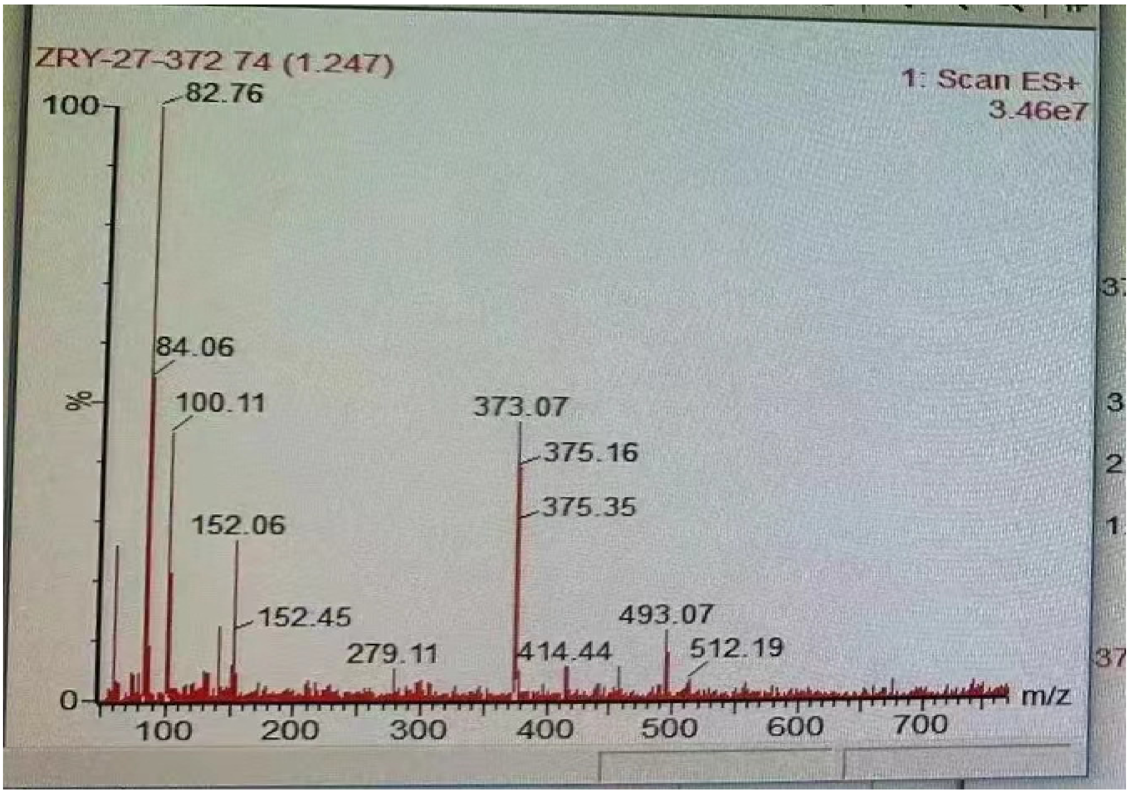

Figure S20 MS (**5f**)

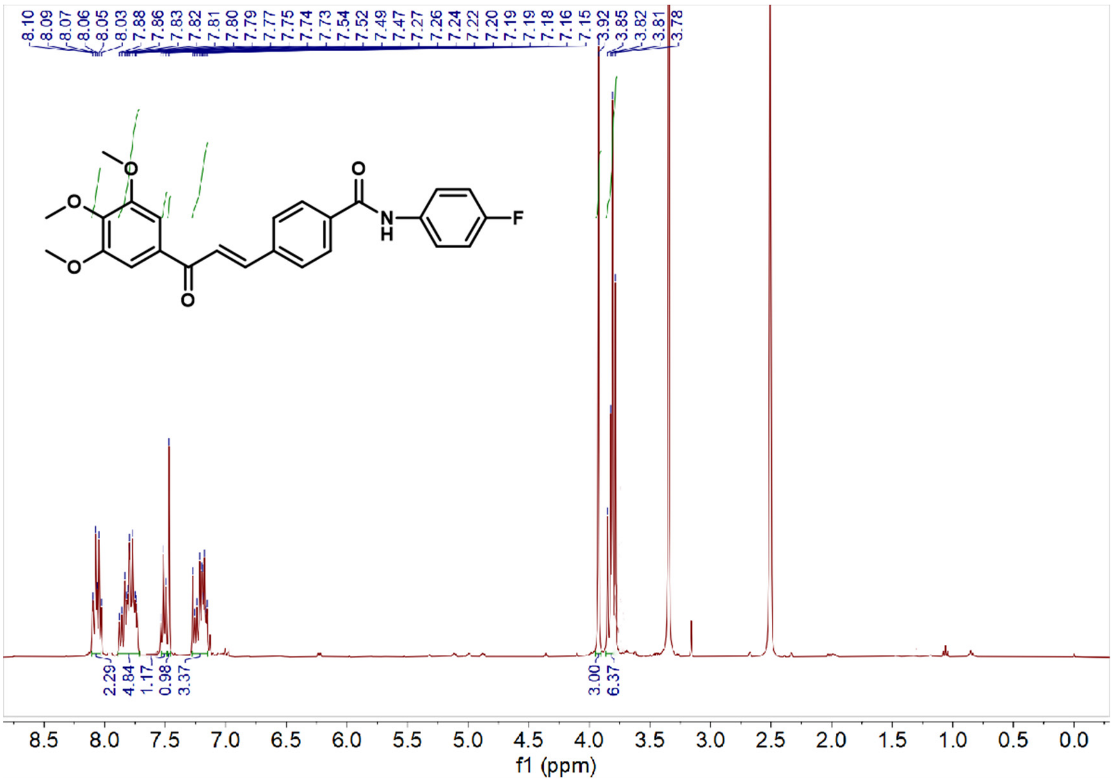

Figure S21 ^{1}H-NMR（**A1**）

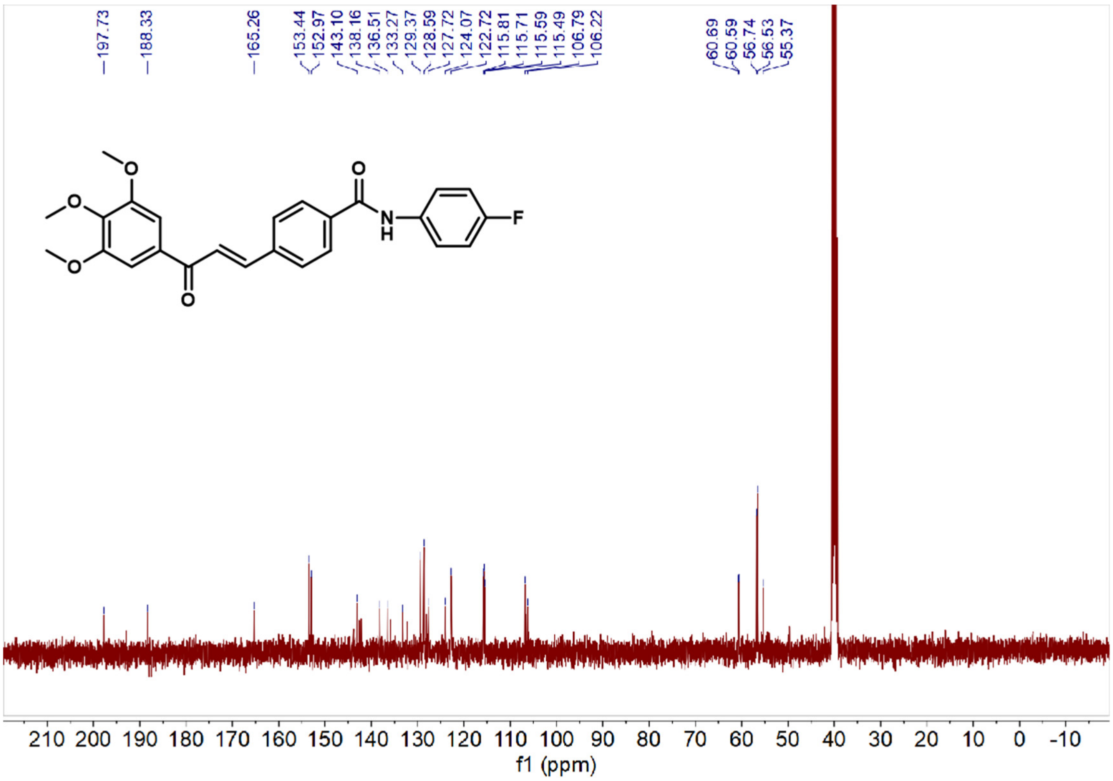

Figure S22 ^{13}C-NMR（**A1**）

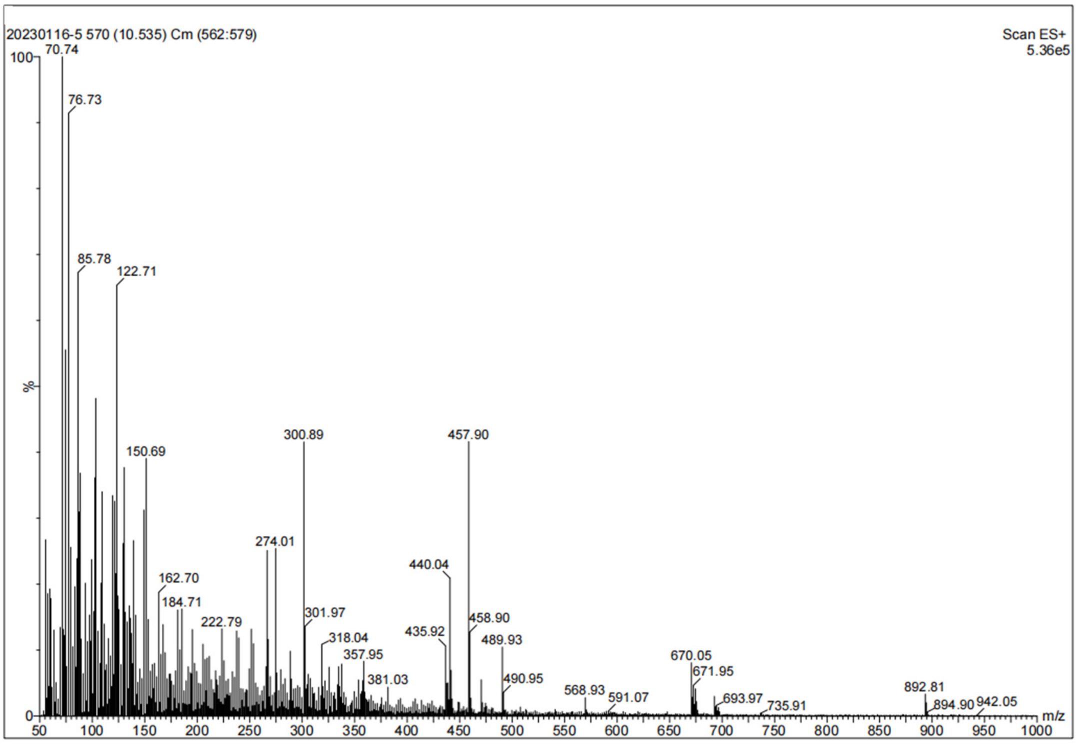

Figure S23 MS (**A1**)

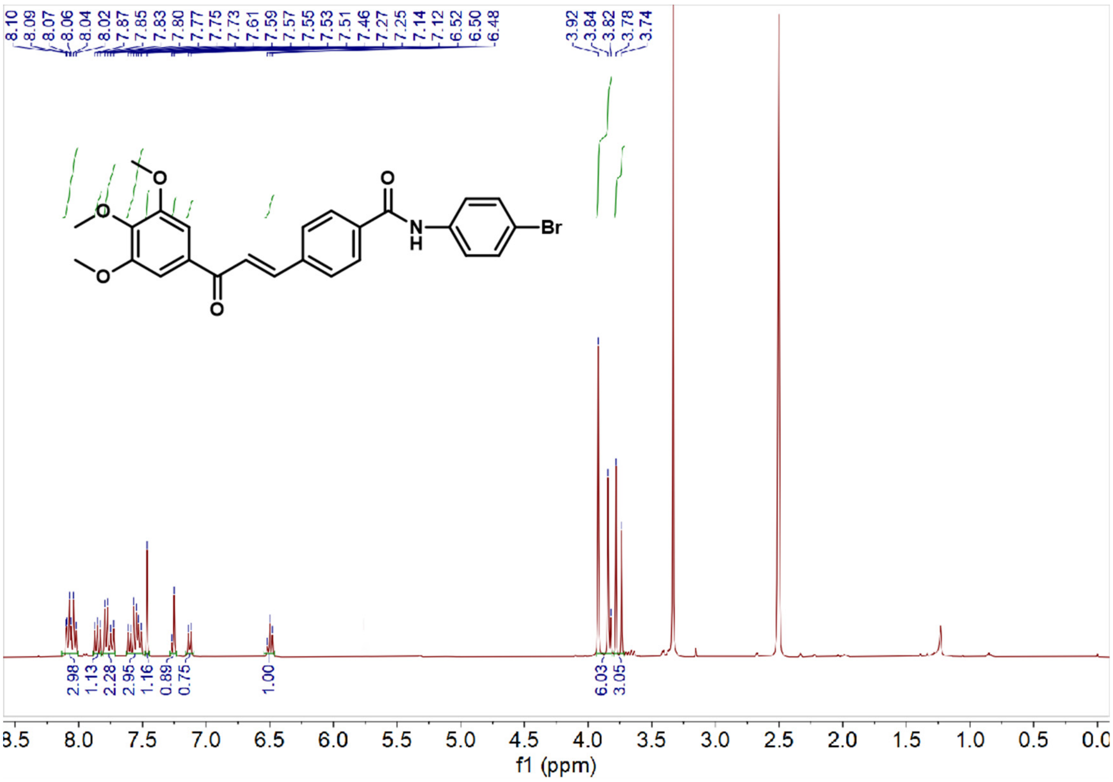

Figure S24 ^{1}H-NMR（**A2**）

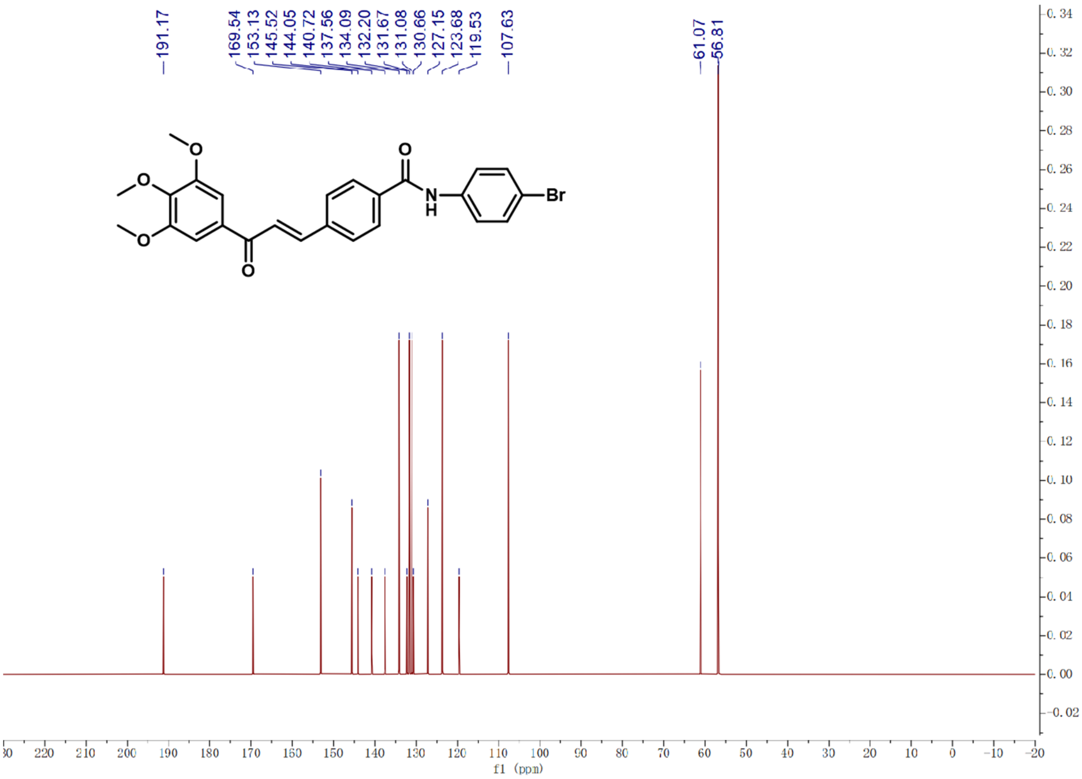

Figure S25 ^{13}C-NMR (**A2**)

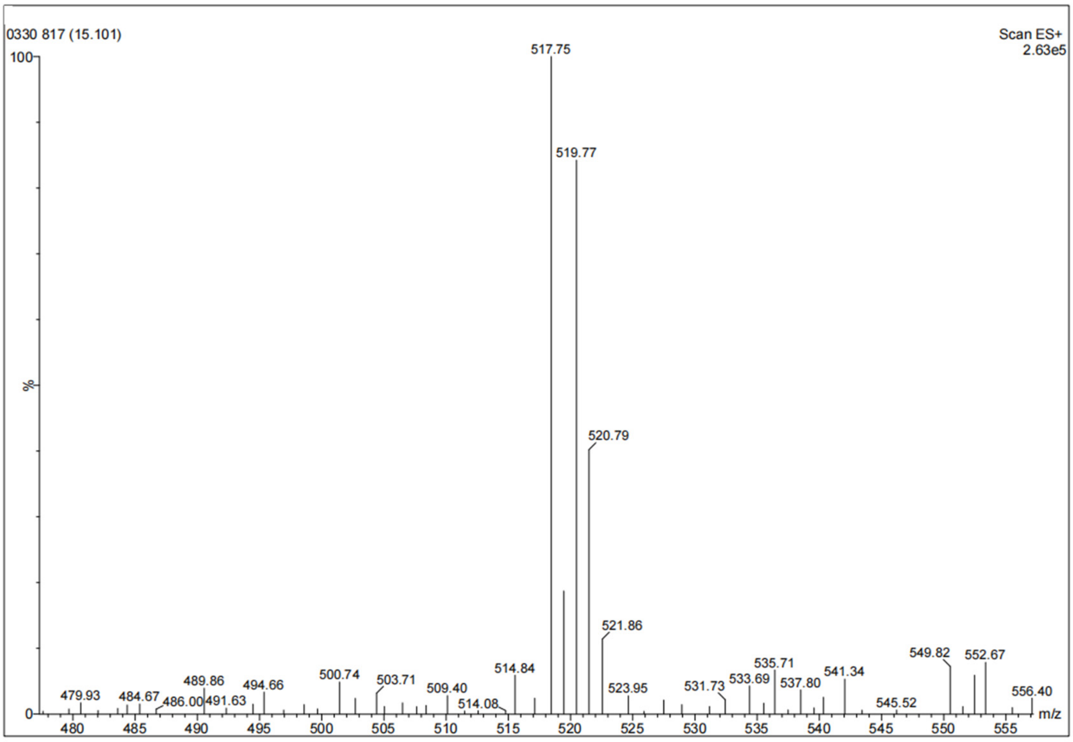

Figure S26 MS (**A2**)

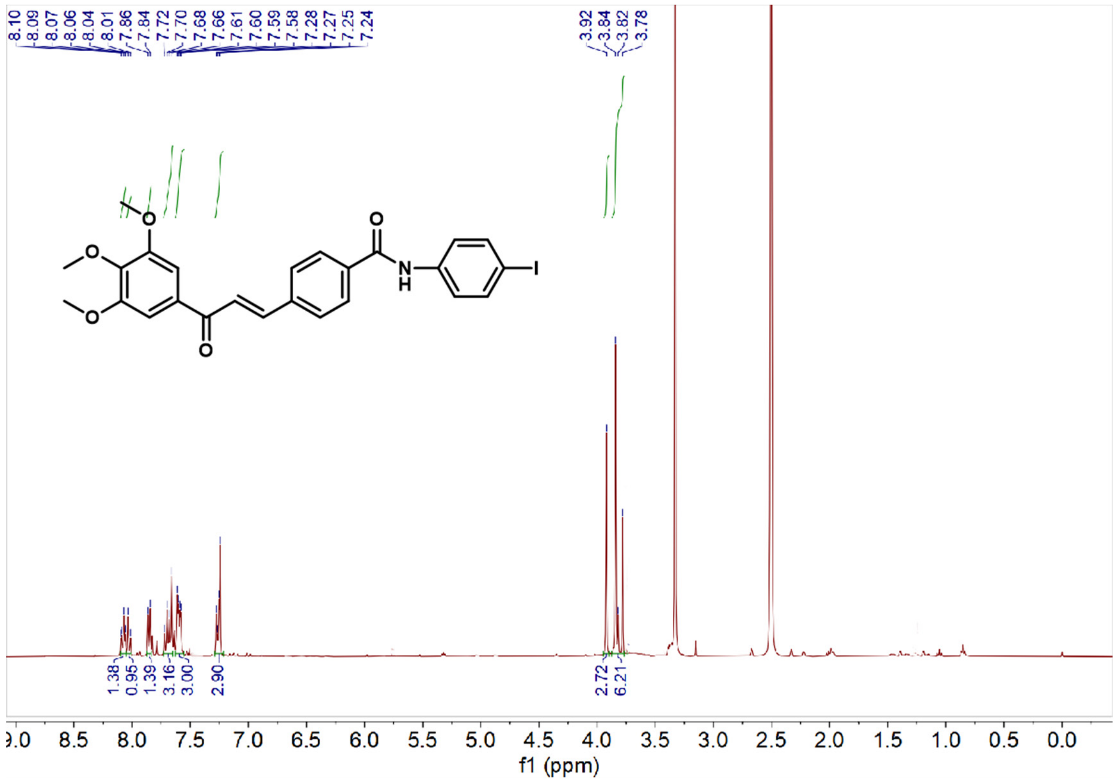

Figure S27 ^{1}H-NMR（**A3**）

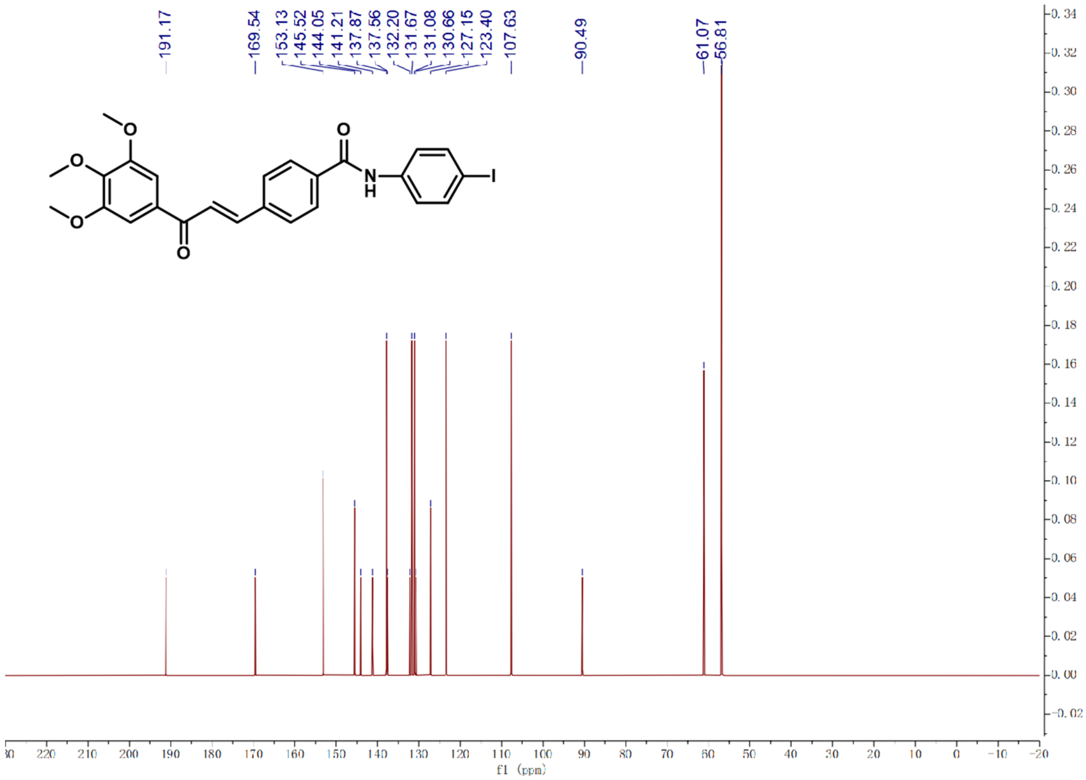

Figure S28 ^{13}C-NMR (**A3**)

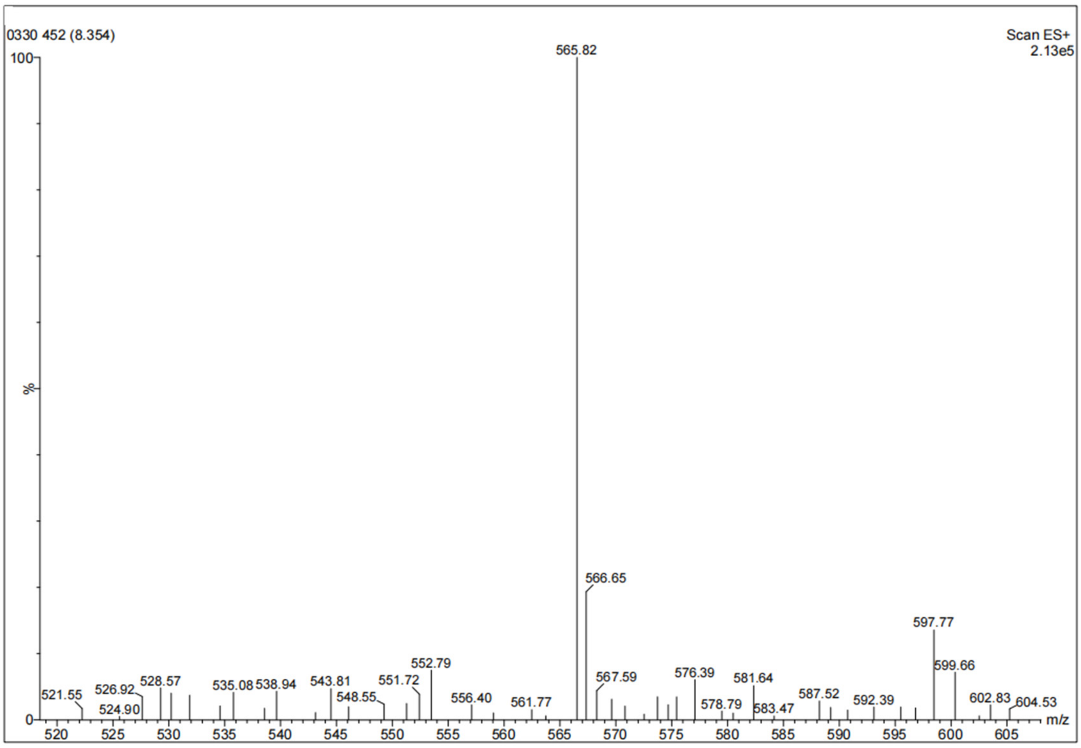

Figure S29 MS (**A3**)

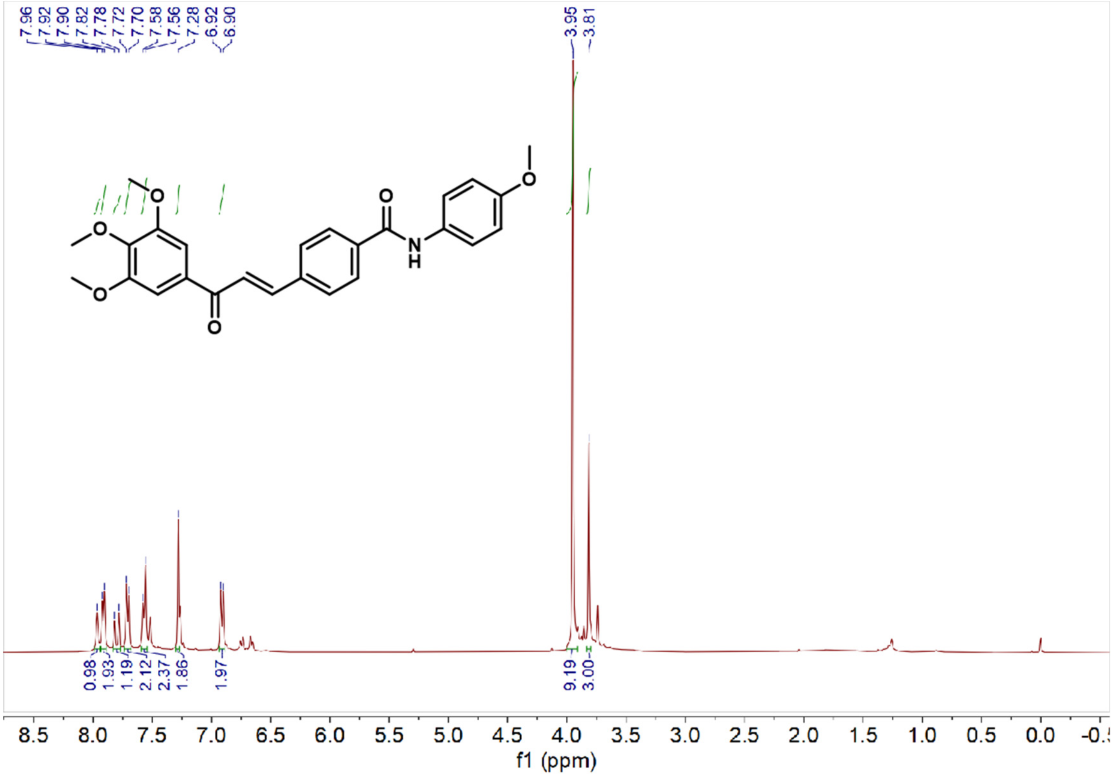

Figure S30 ^{1}H-NMR（**A4**）

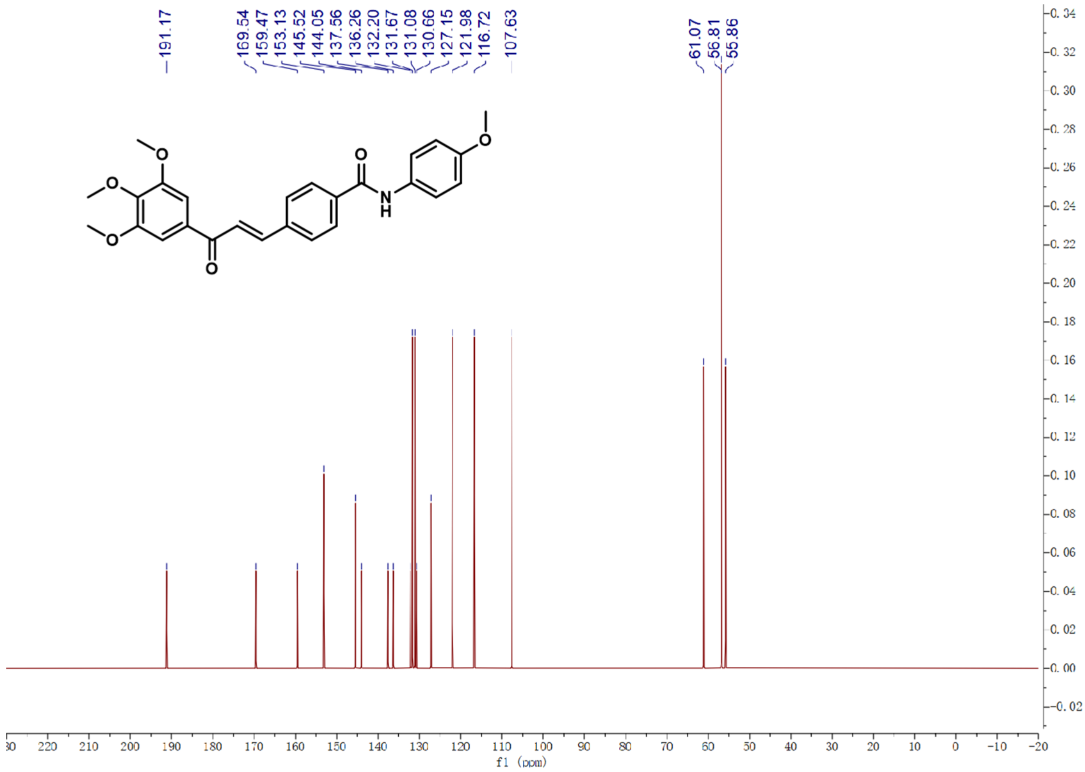

Figure S31 ^{13}C-NMR (**A4**)

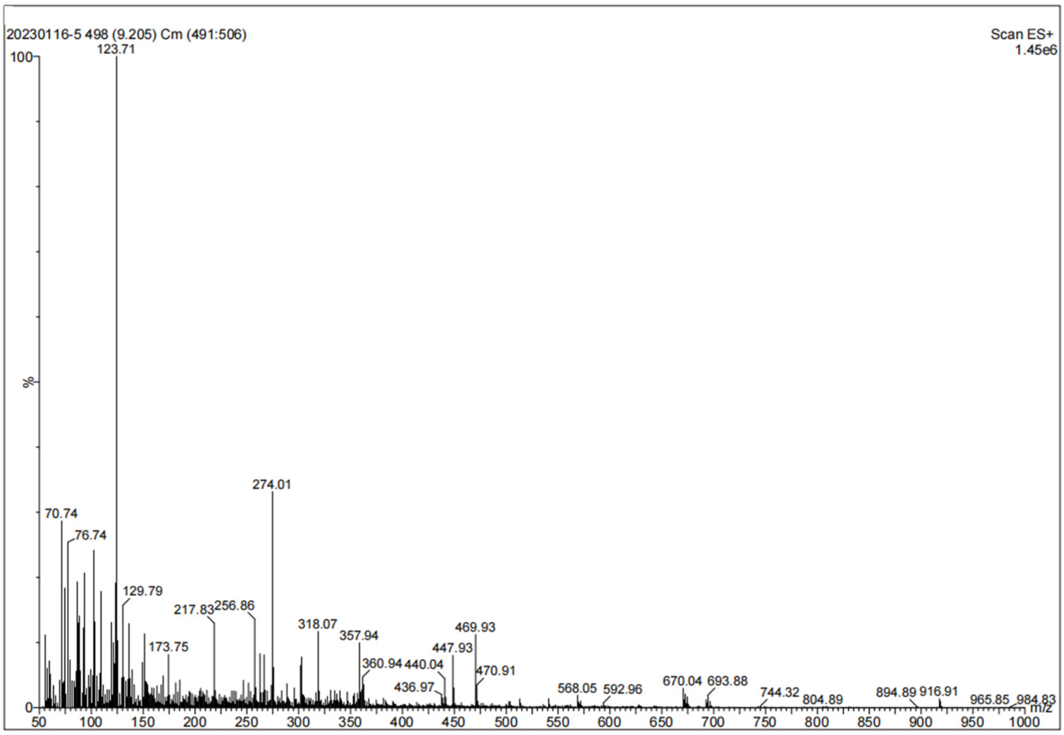

Figure S32 MS (**A4**)

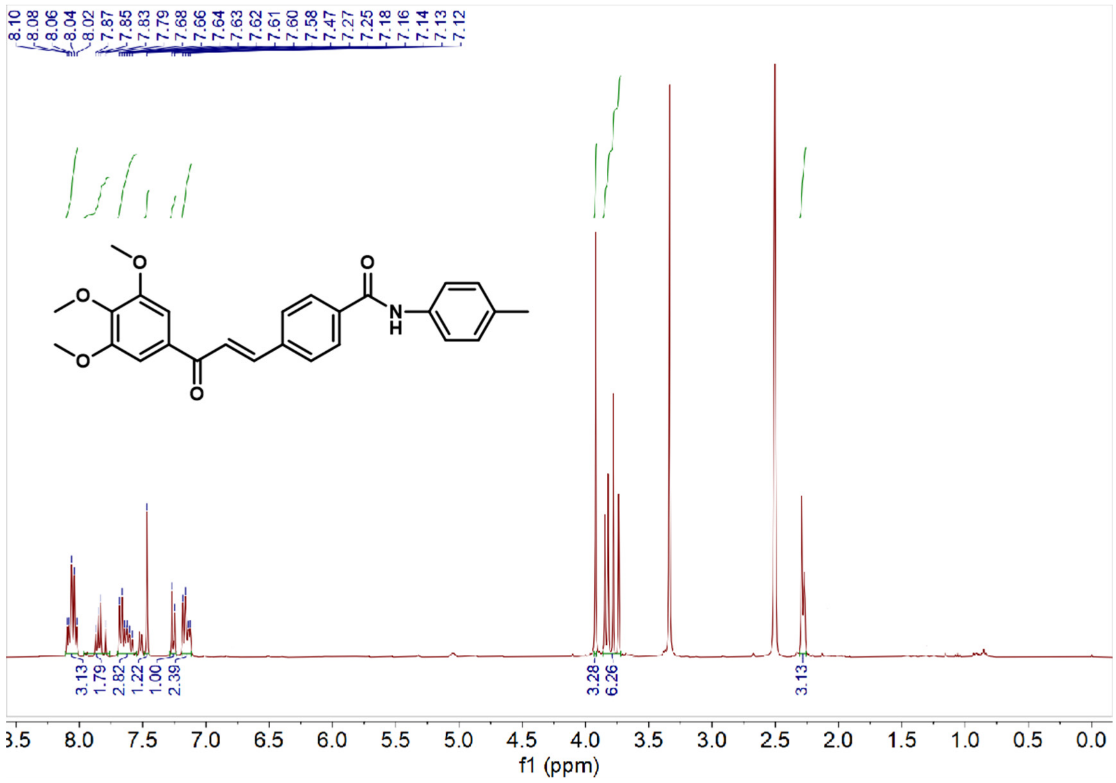

Figure S33 ^{1}H-NMR（**A5**）

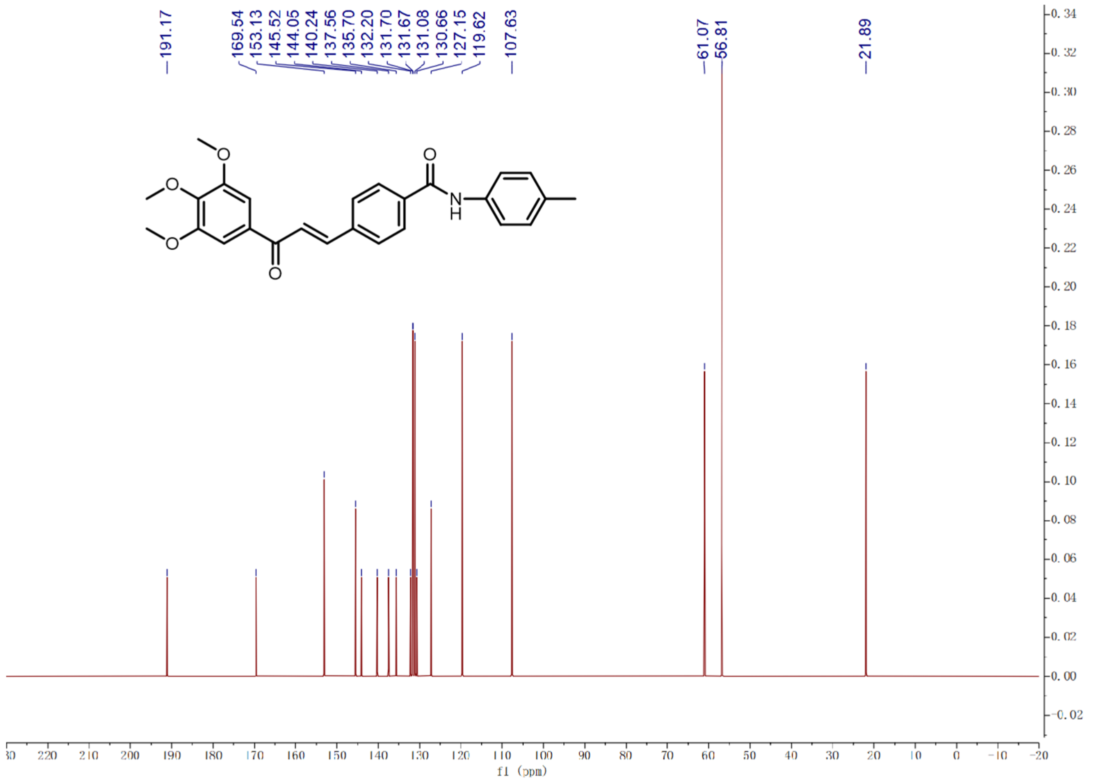

Figure S34 ^{13}C-NMR (**A5**)

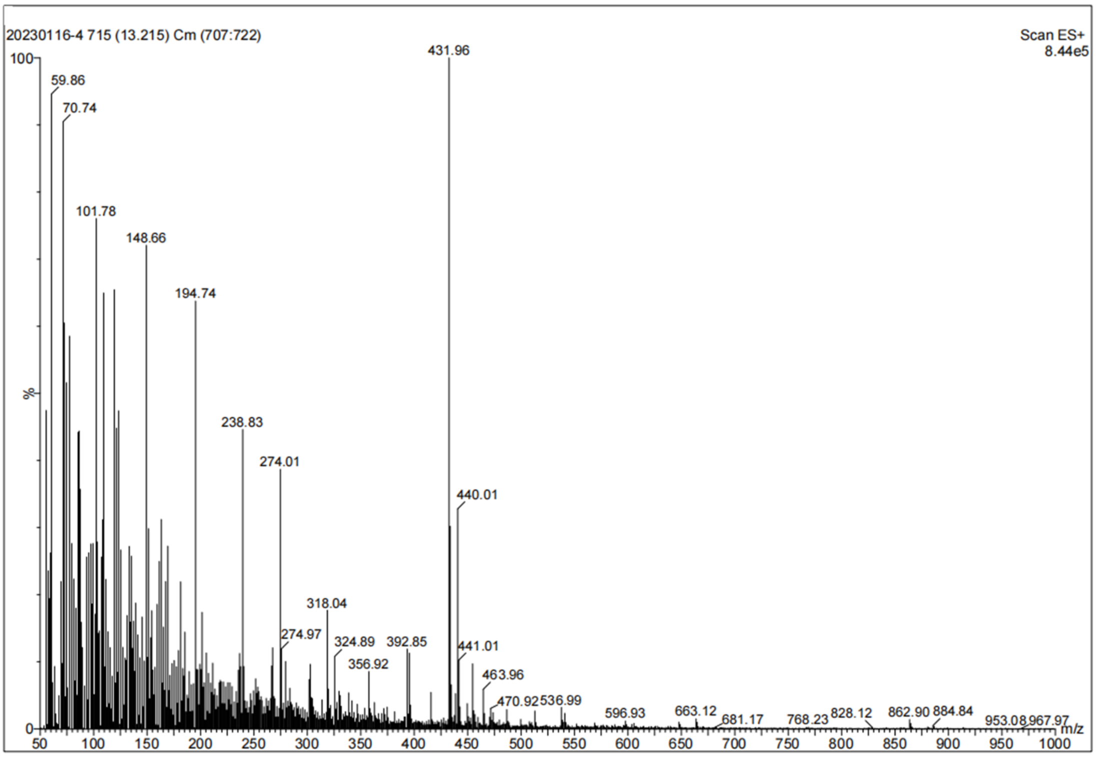

Figure S35 MS (**A5**)

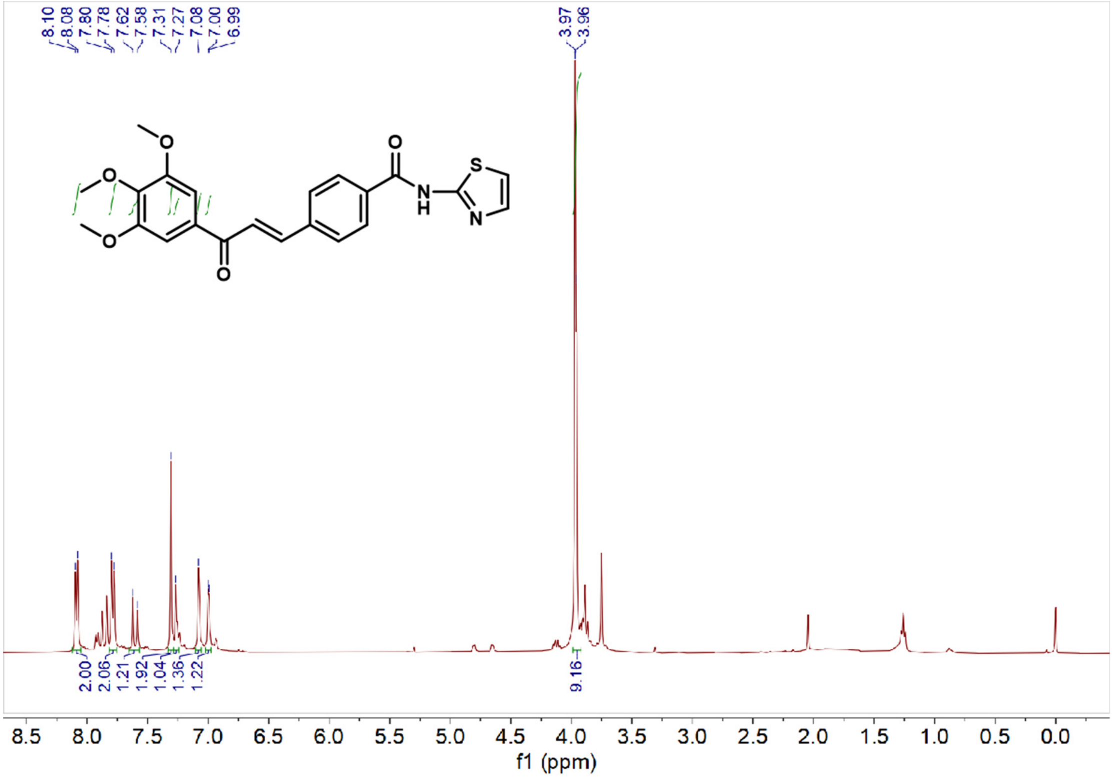

Figure S36 ^{1}H-NMR（**A6**）

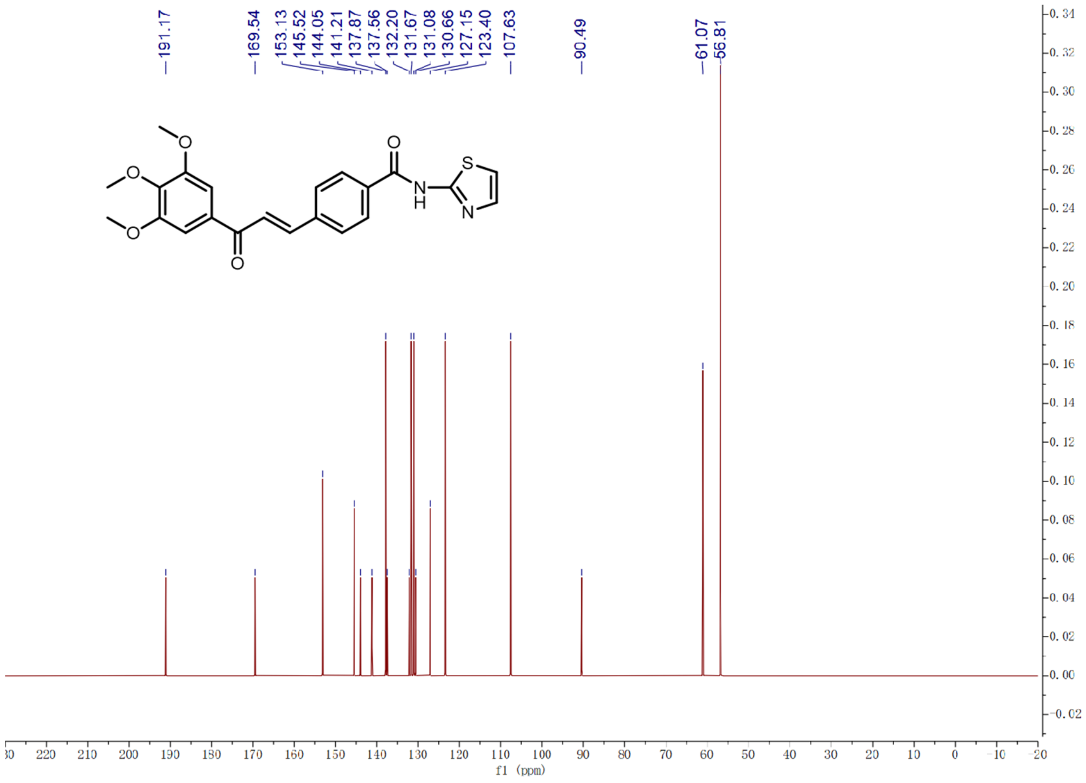

Figure S37 ^{13}C-NMR (**A6**)

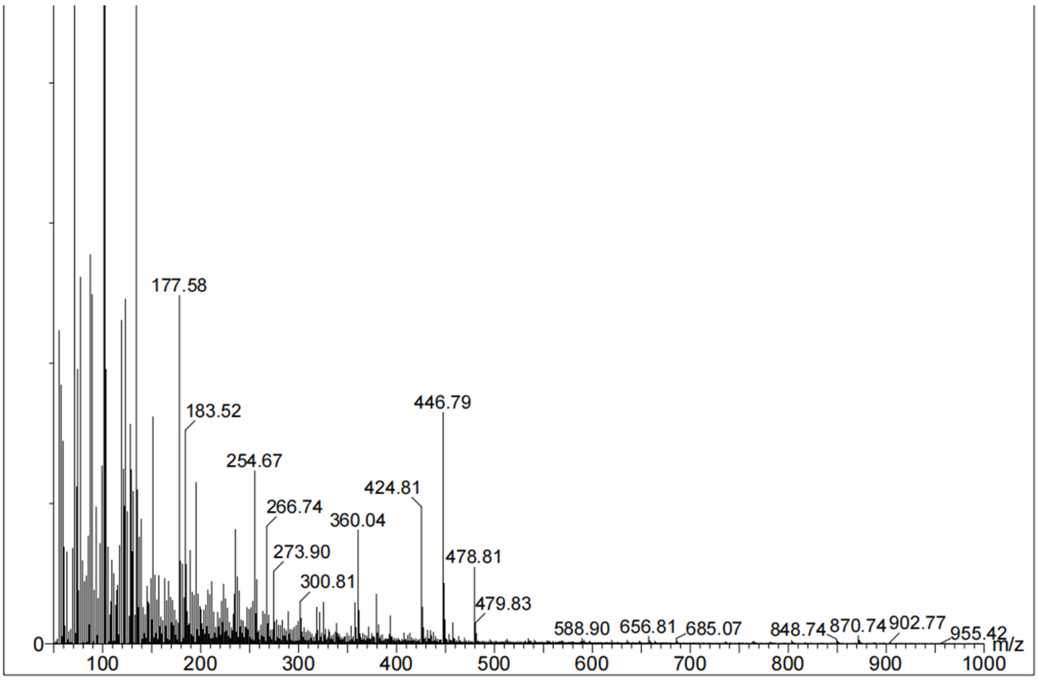

Figure S38 MS (**A6**)

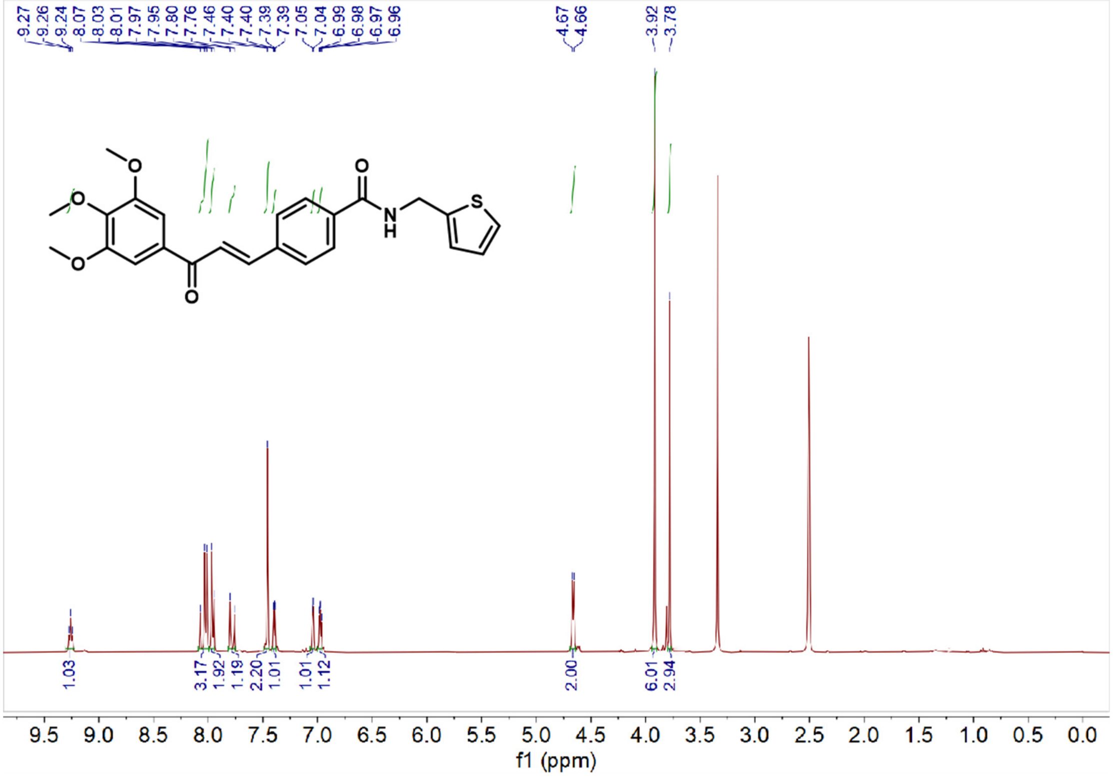

Figure S39 ^{1}H-NMR（**A7**）

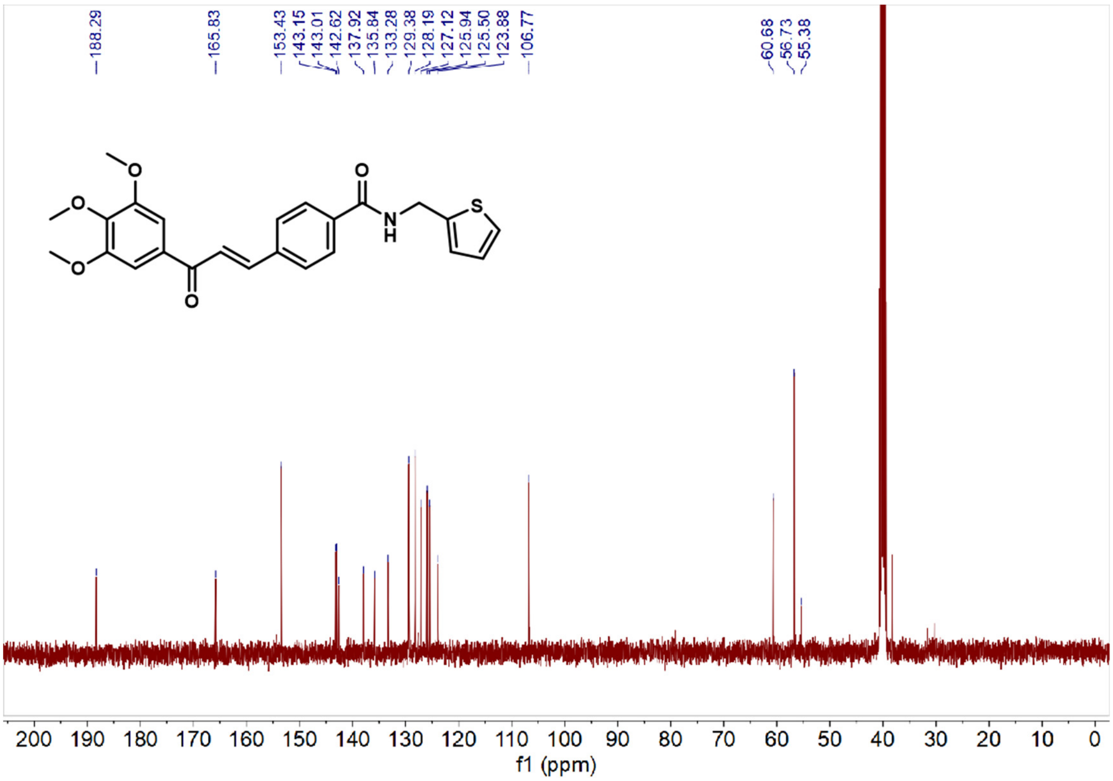

Figure S40 ^{13}C-NMR（**A7**）

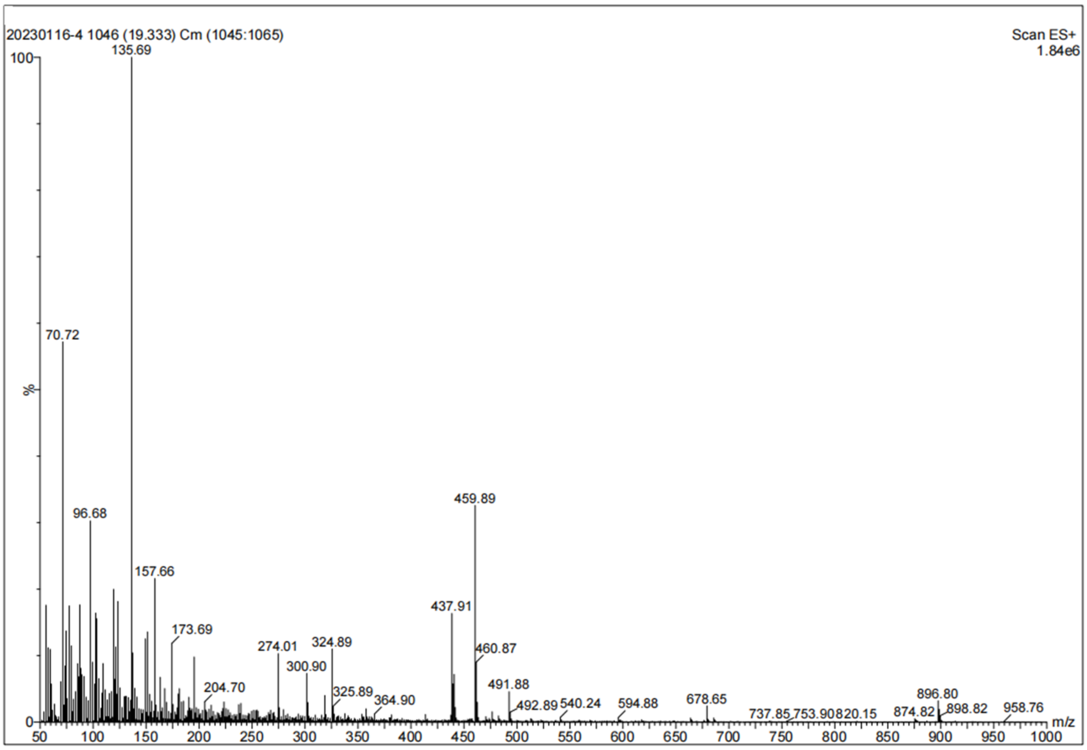

Figure S41 MS（**A7**）

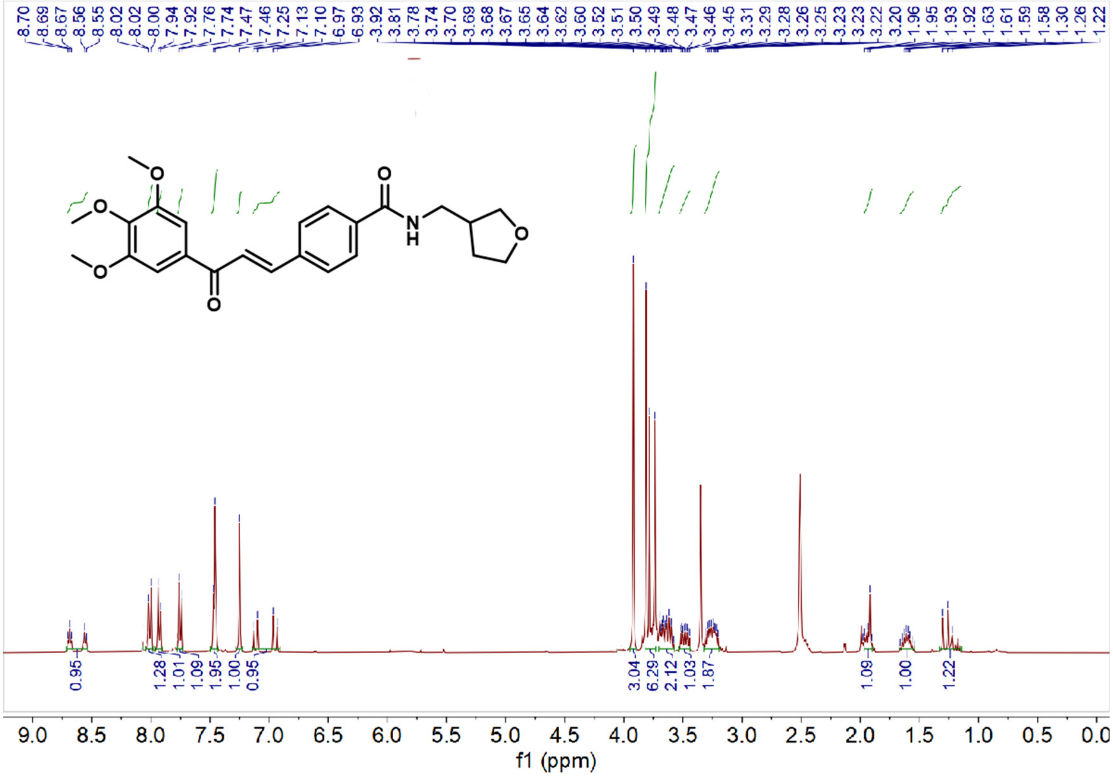

Figure S42 ^{1}H-NMR（**A8**）

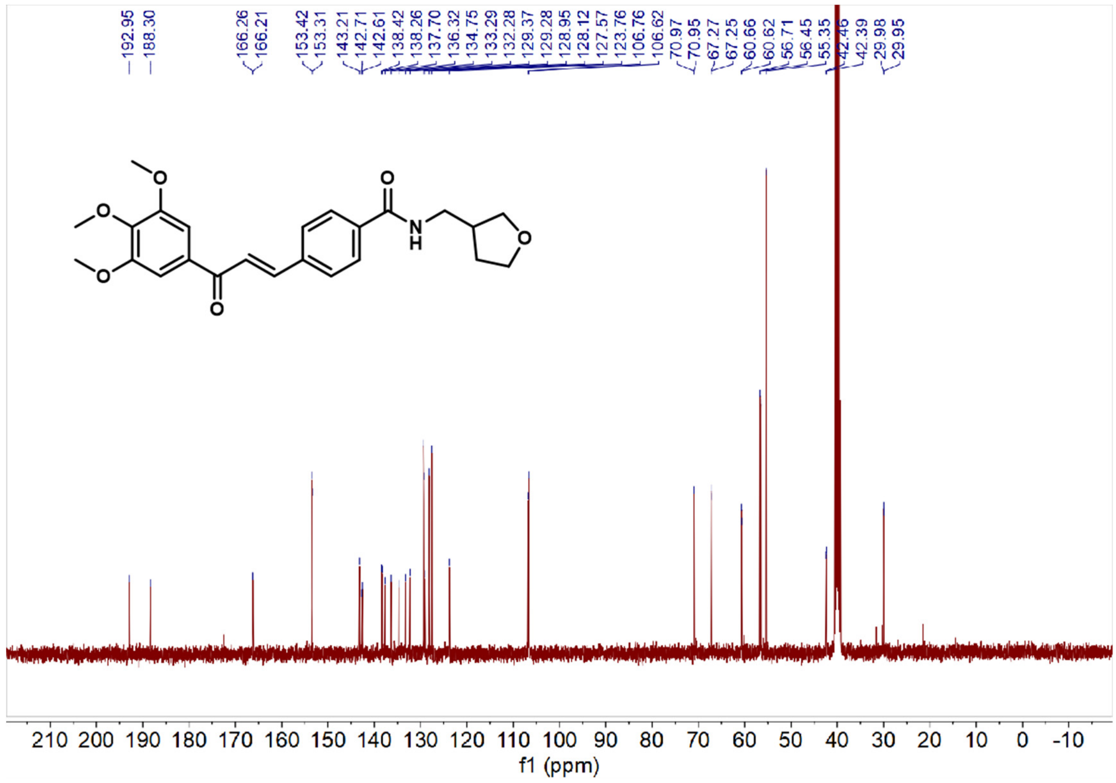

Figure S43 ^{13}C-NMR（**A8**）

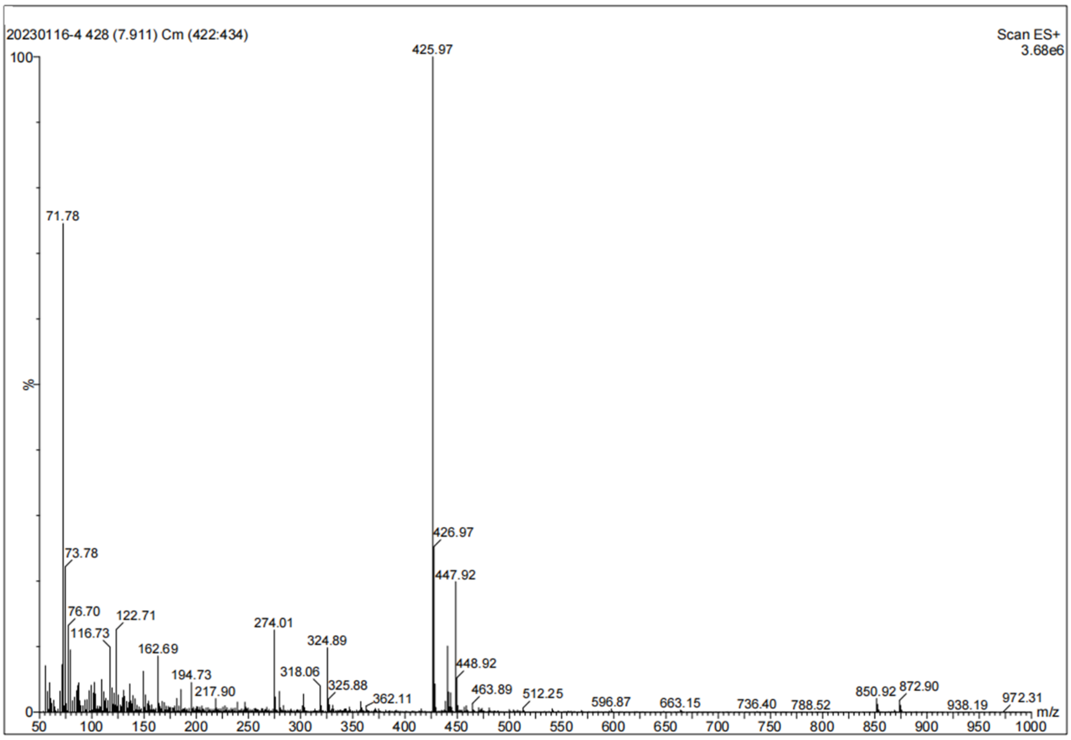

Figure S44 MS（**A8**）

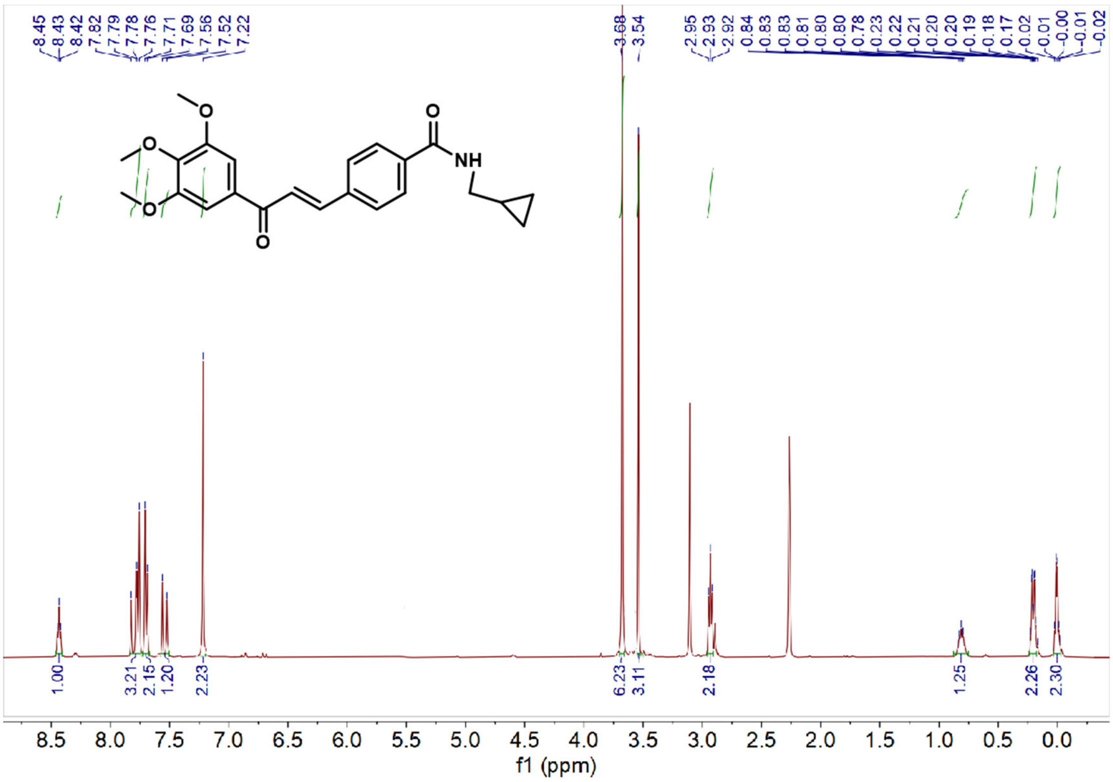

Figure S45 ^{1}H-NMR（**A9**）

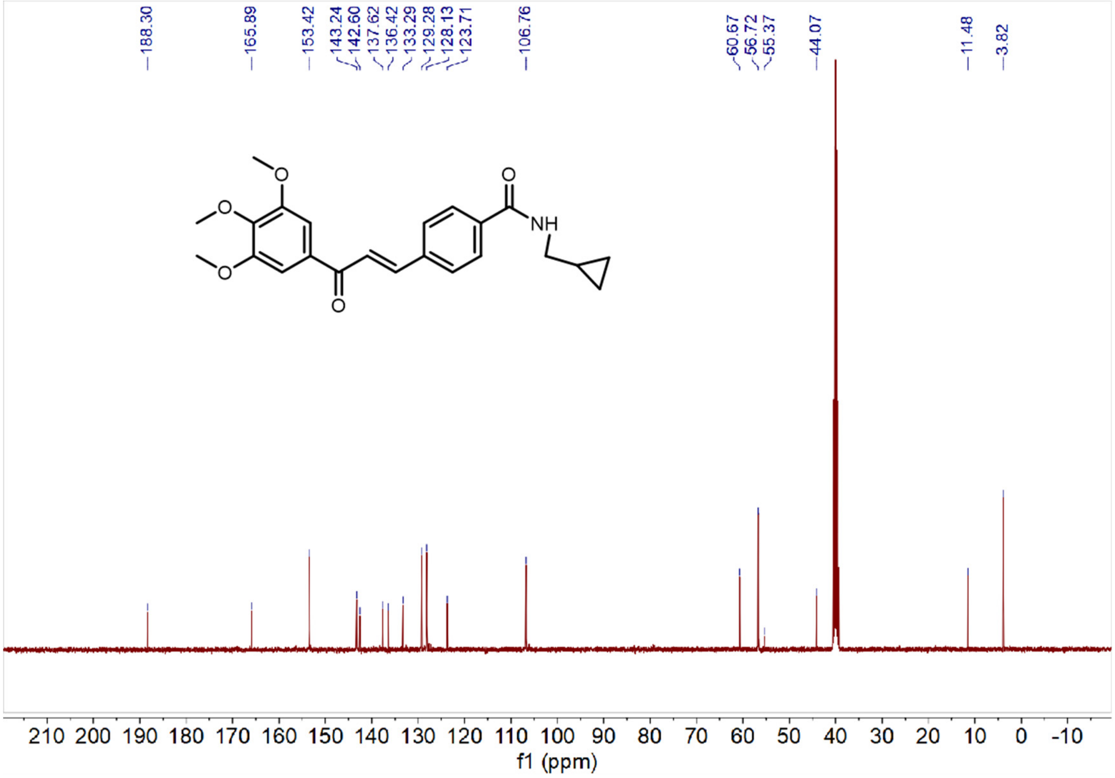

Figure S46 ^{13}C-NMR（**A9**）

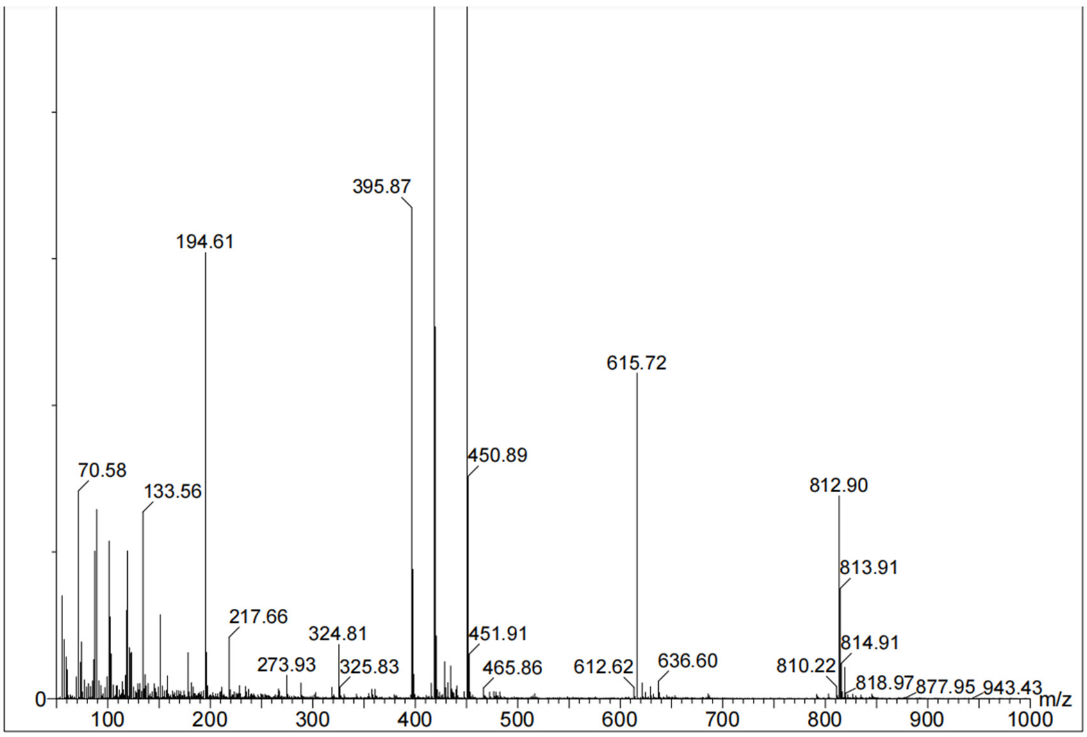

Figure S47 MS（**A9**）

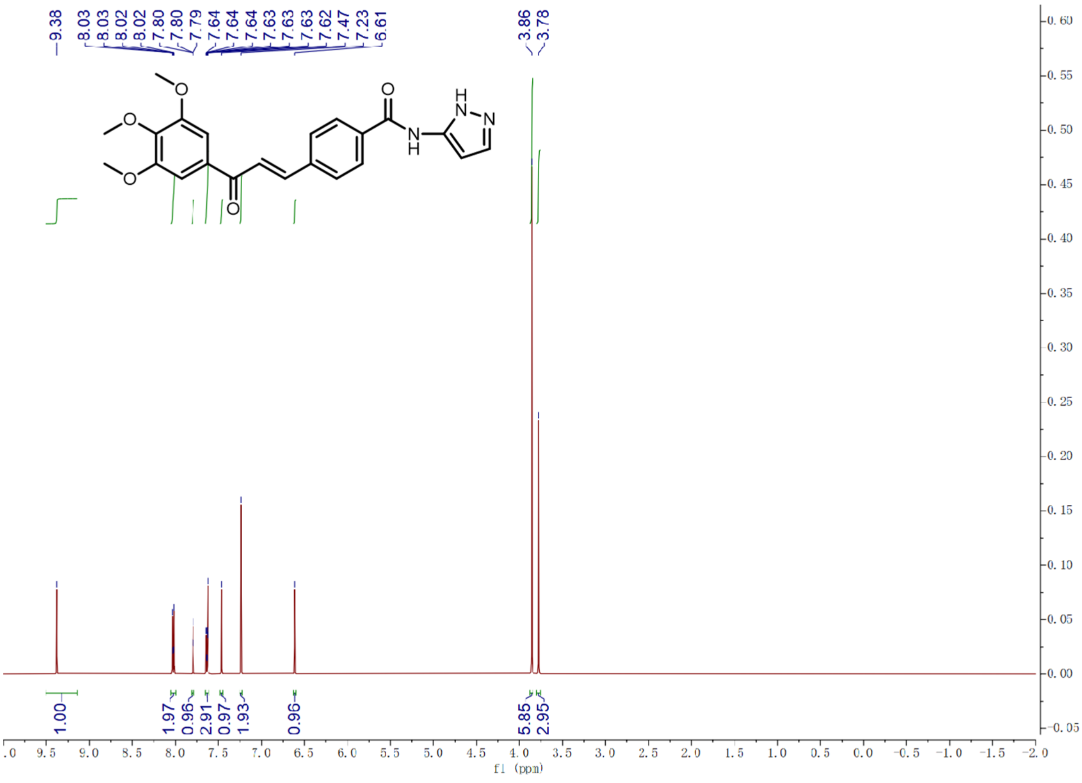

Figure S48 ^{1}H-NMR（**A10**）

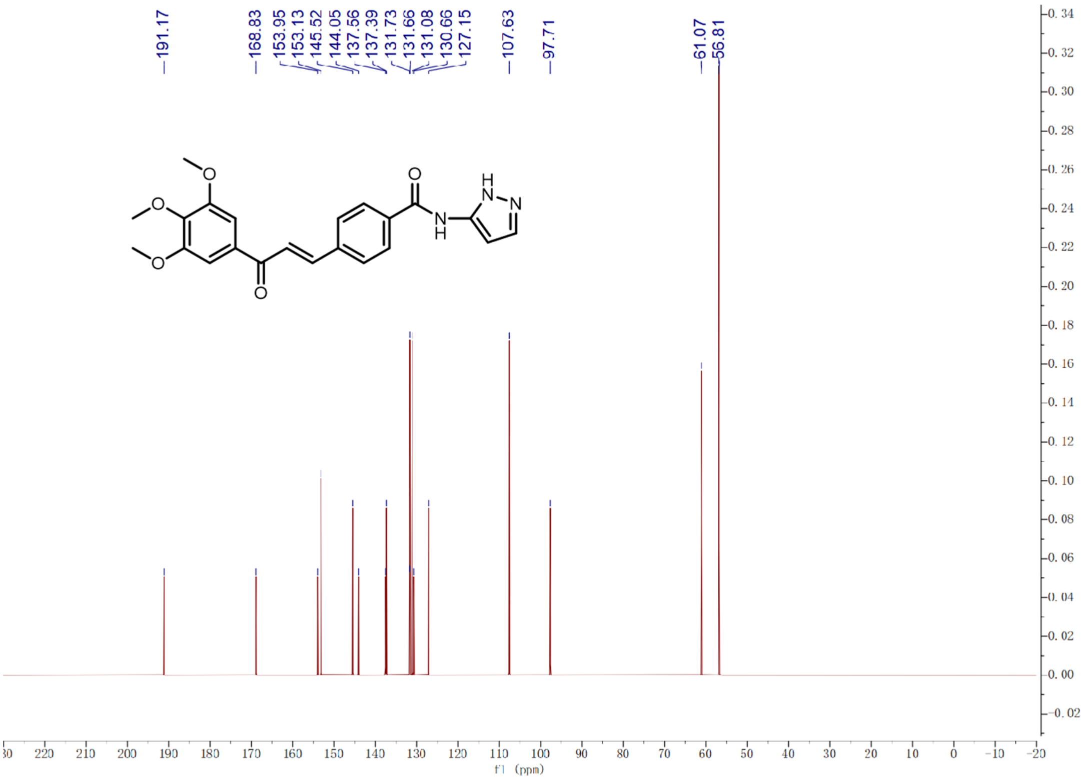

Figure S49 ^{13}C-NMR（**A10**）

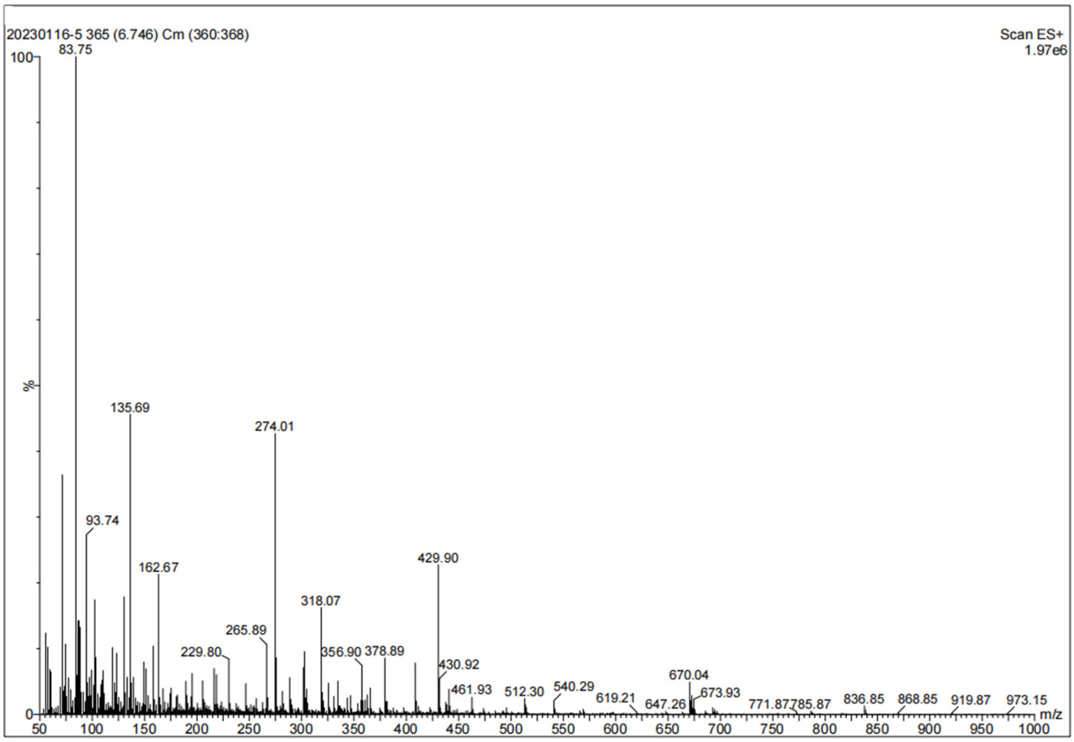

Figure S50 MS（**A10**）

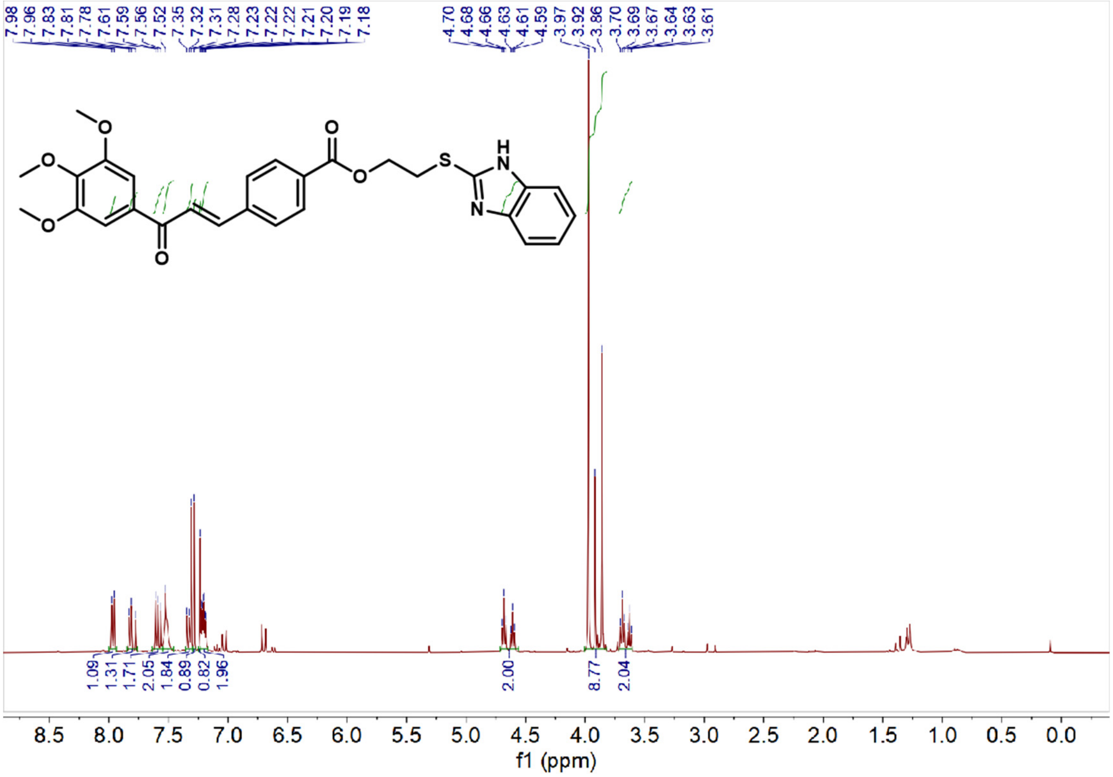

Figure S51 ^{1}H-NMR（**A11**）

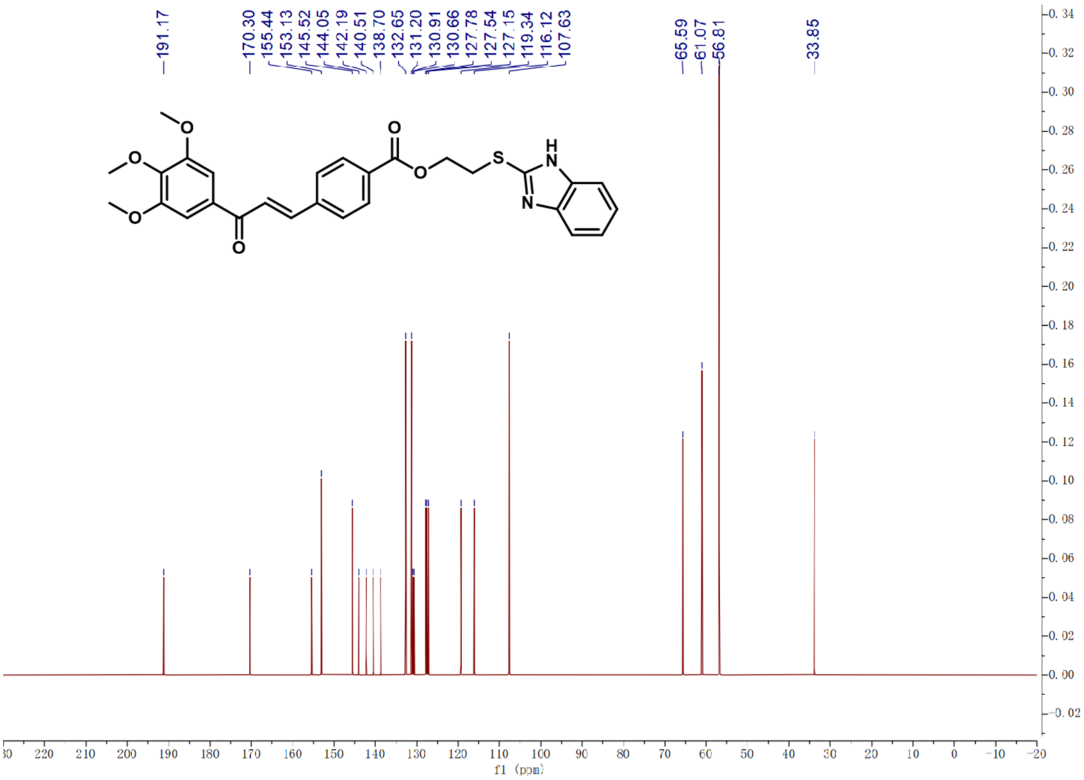

Figure S52 ^{13}C-NMR (**A11**)

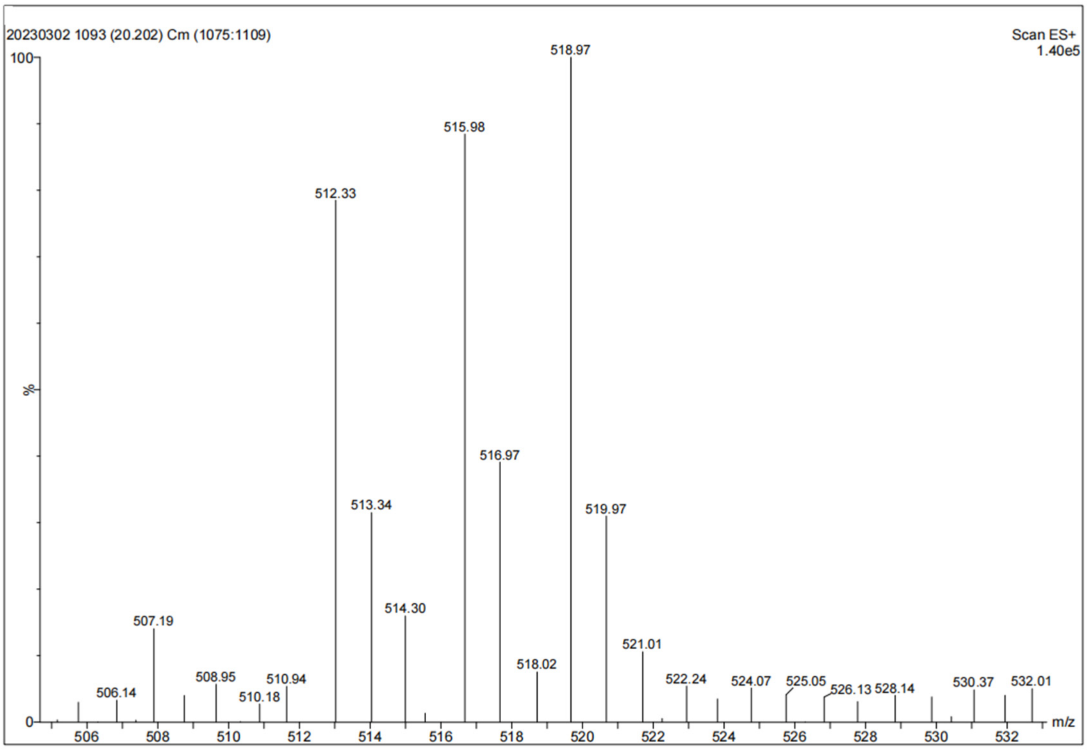

Figure S53 MS（**A11**）

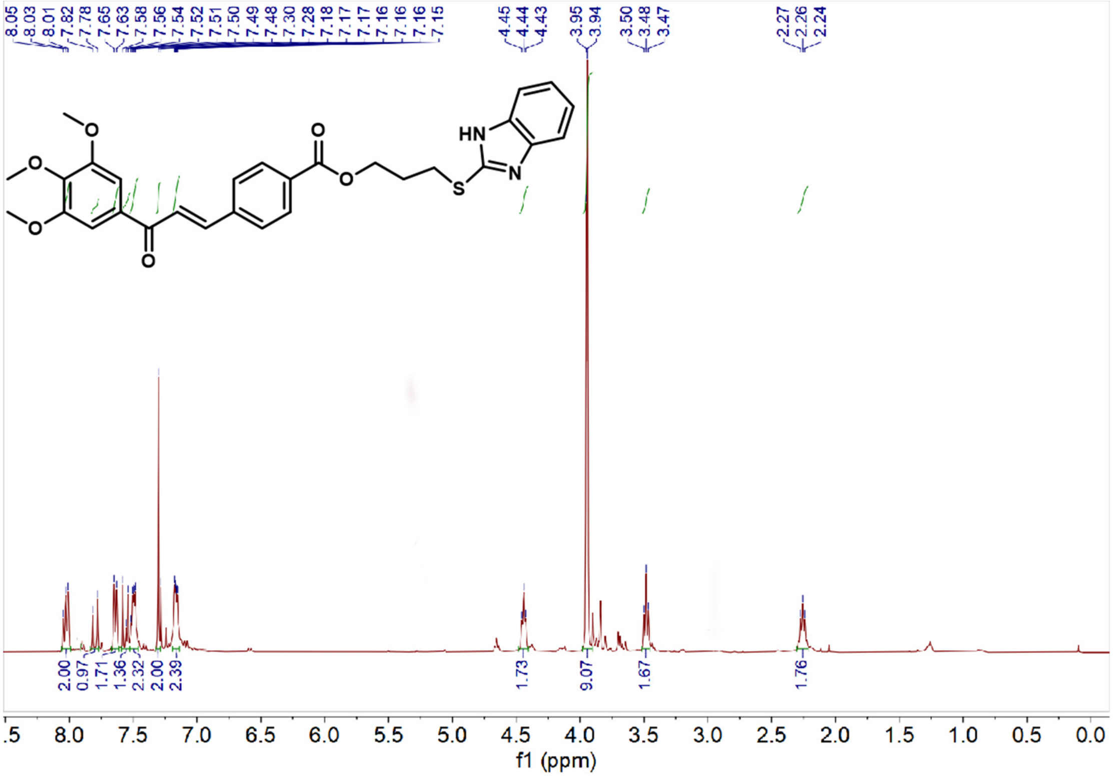

Figure S54 ^{1}H-NMR（**A12**）

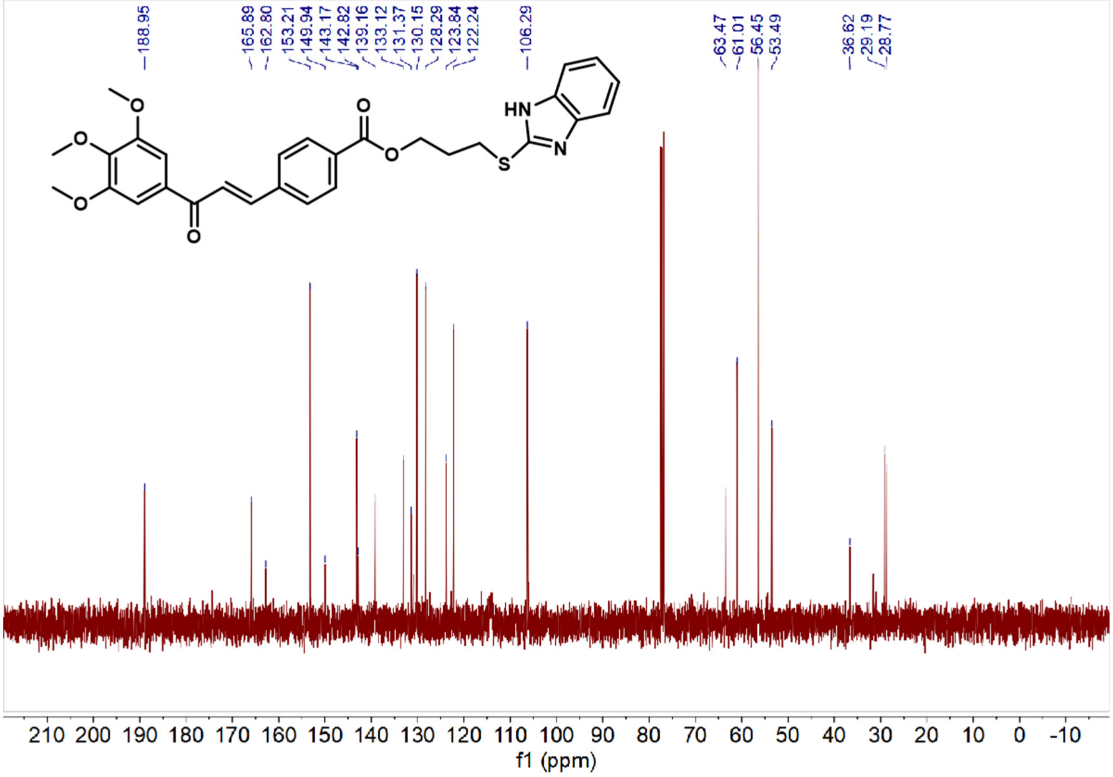

Figure S55 ^{13}C-NMR（**A12**）

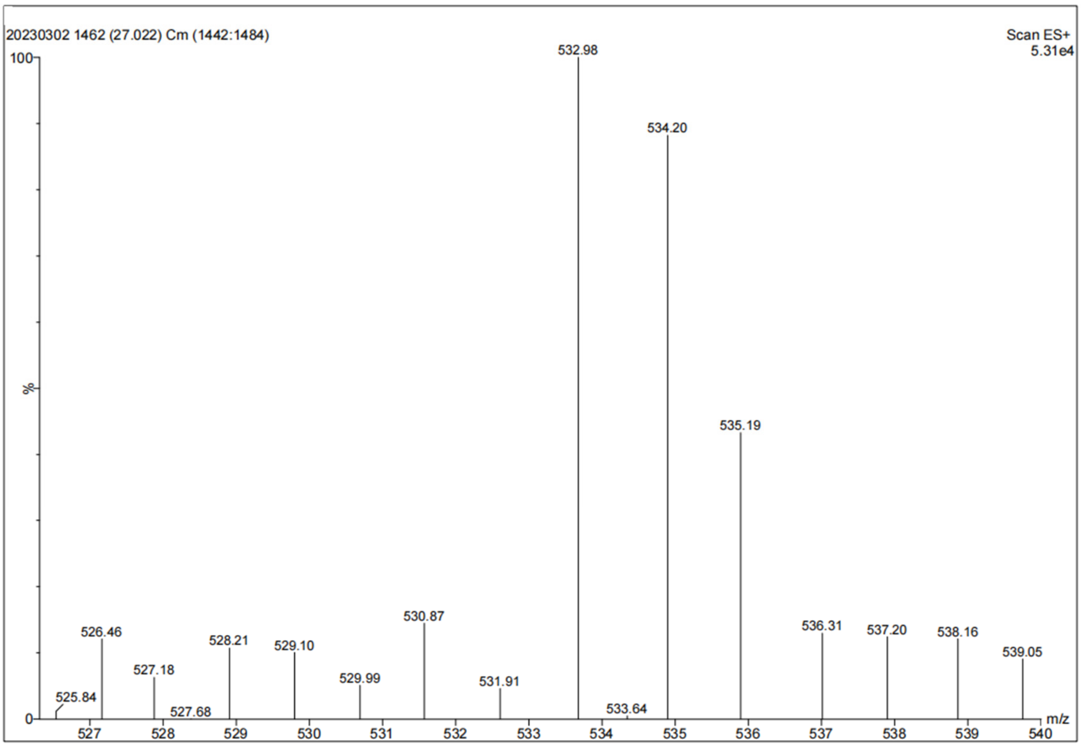

Figure S56 MS（**A12**）

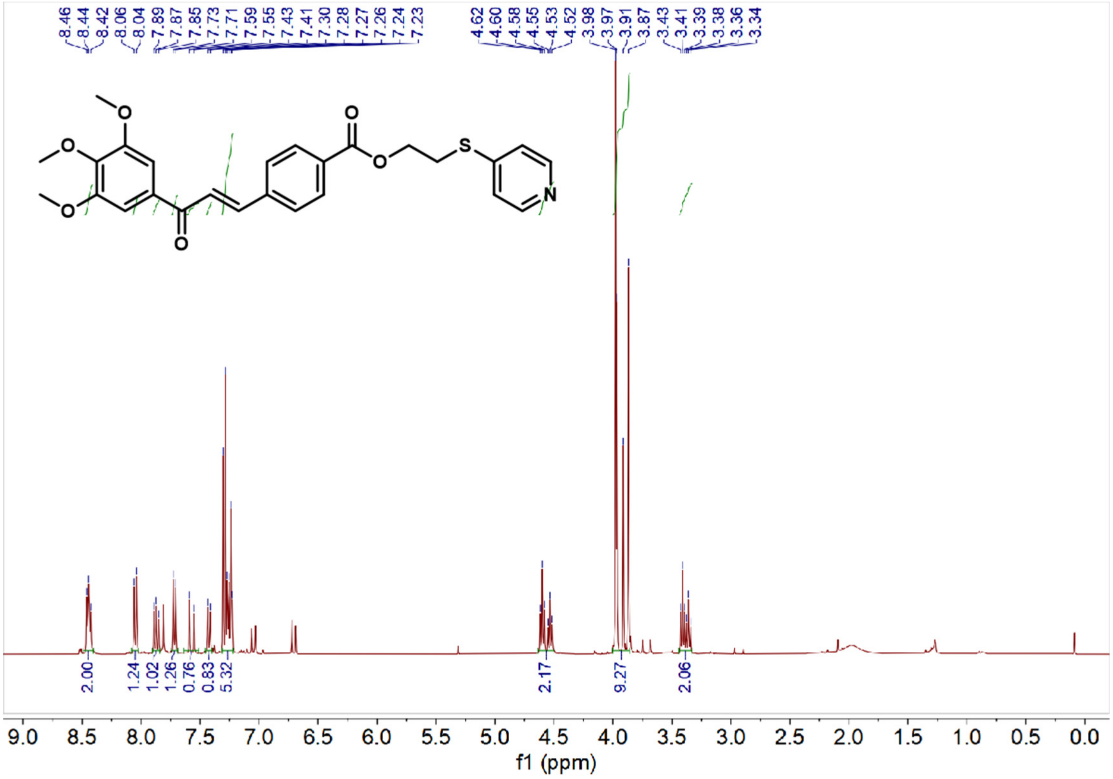

Figure S57 ^{1}H-NMR（**A13**）

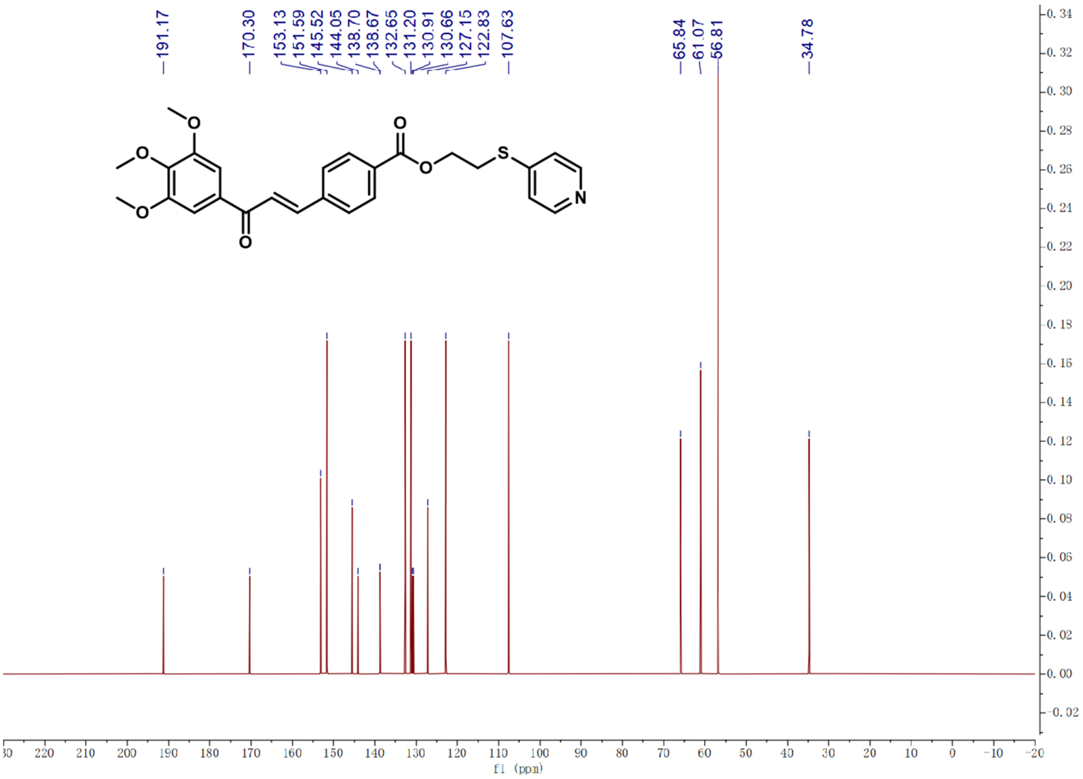

Figure S58 ^{13}C-NMR (**A13**)

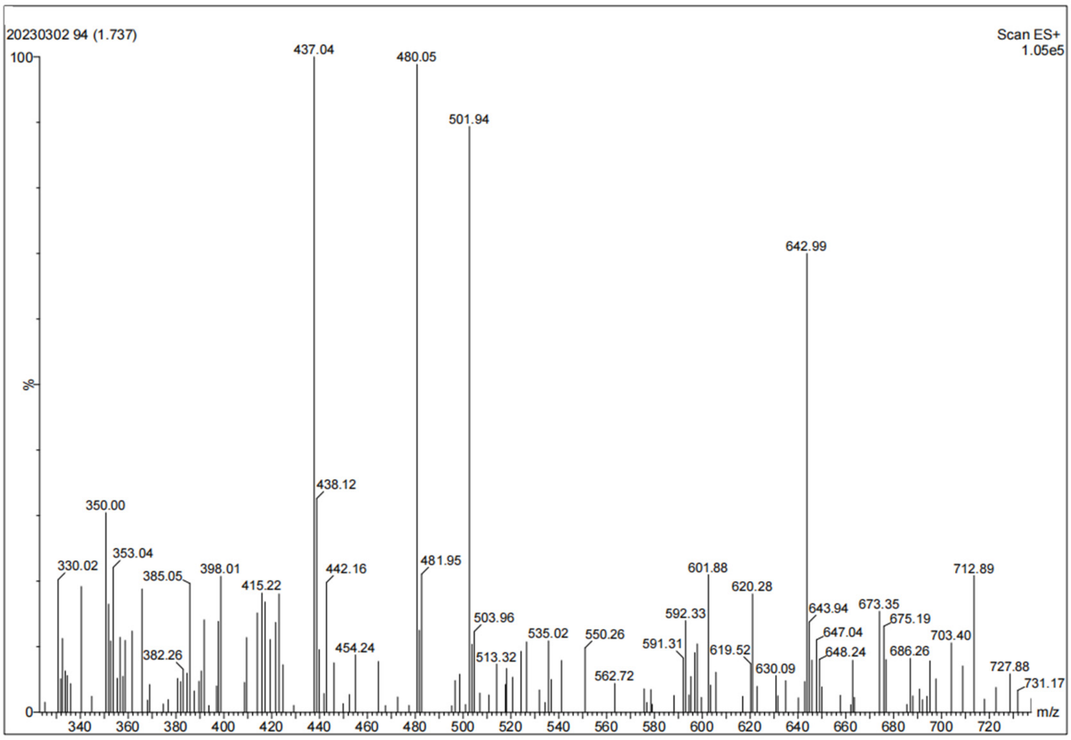

Figure S59 MS（**A13**）

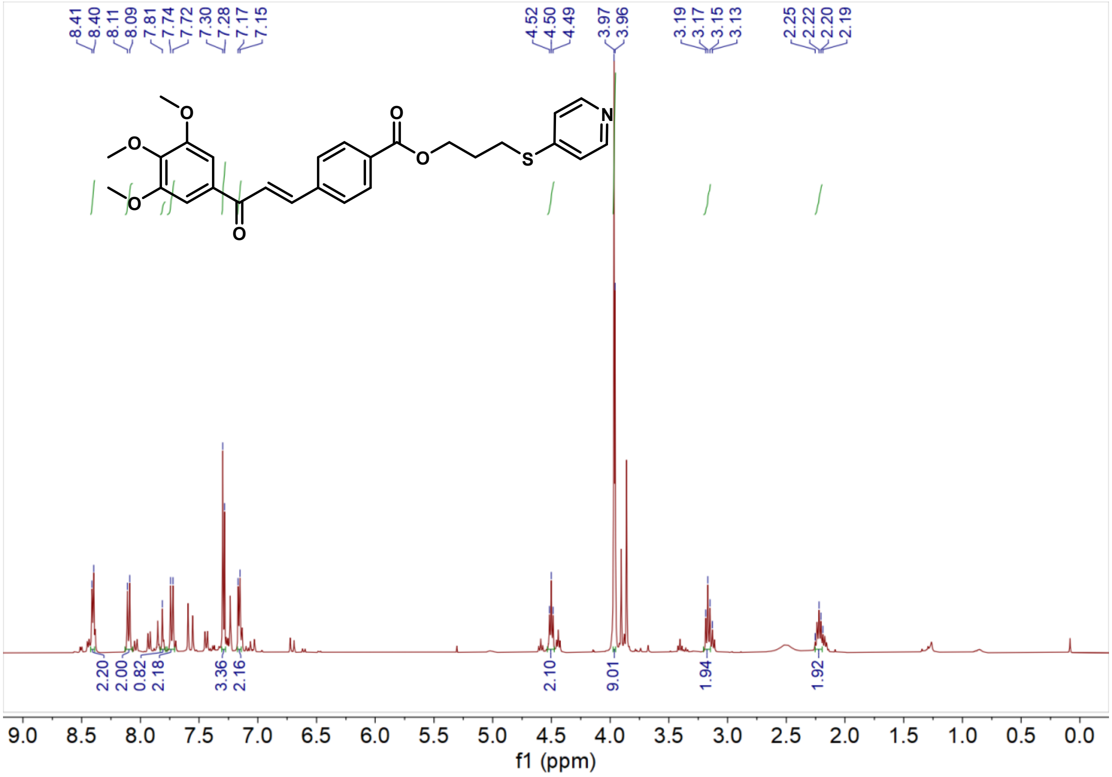

Figure S60 ^{1}H-NMR（**A14**）

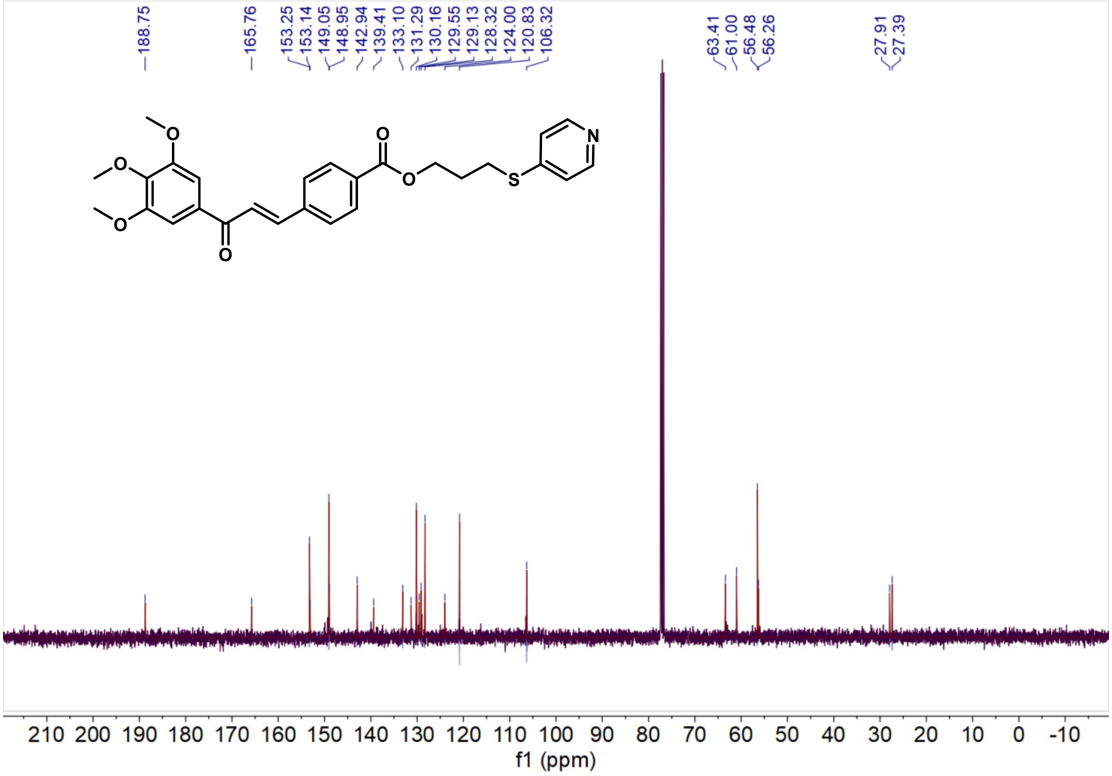

Figure S61 ^{13}C-NMR（**A14**）

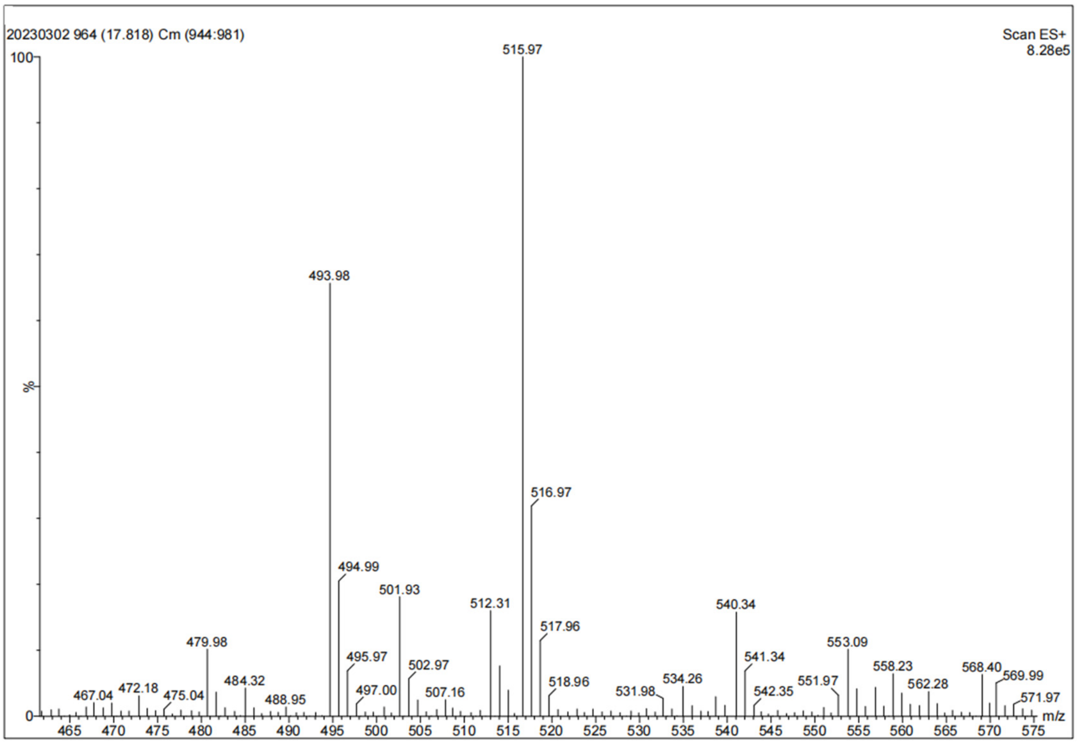

Figure S62 MS（**A14**）

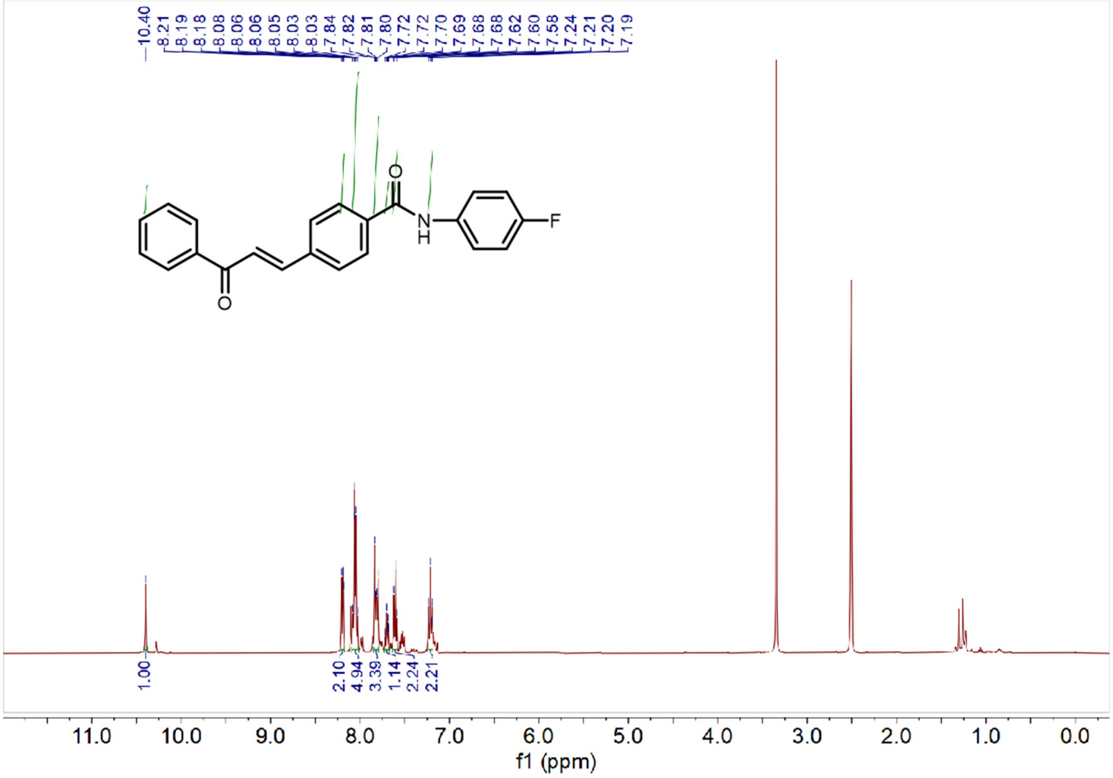

Figure S63 ^{1}H-NMR（**B1**）

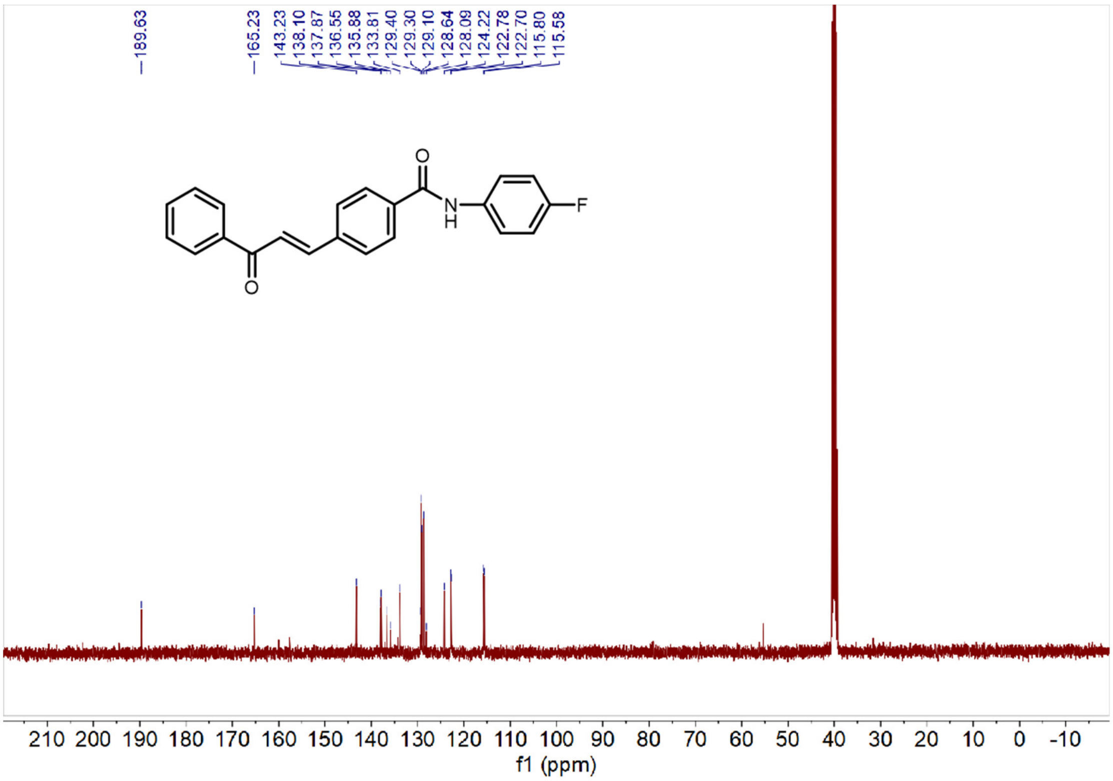

Figure S64 ^{13}C-NMR（**B1**）

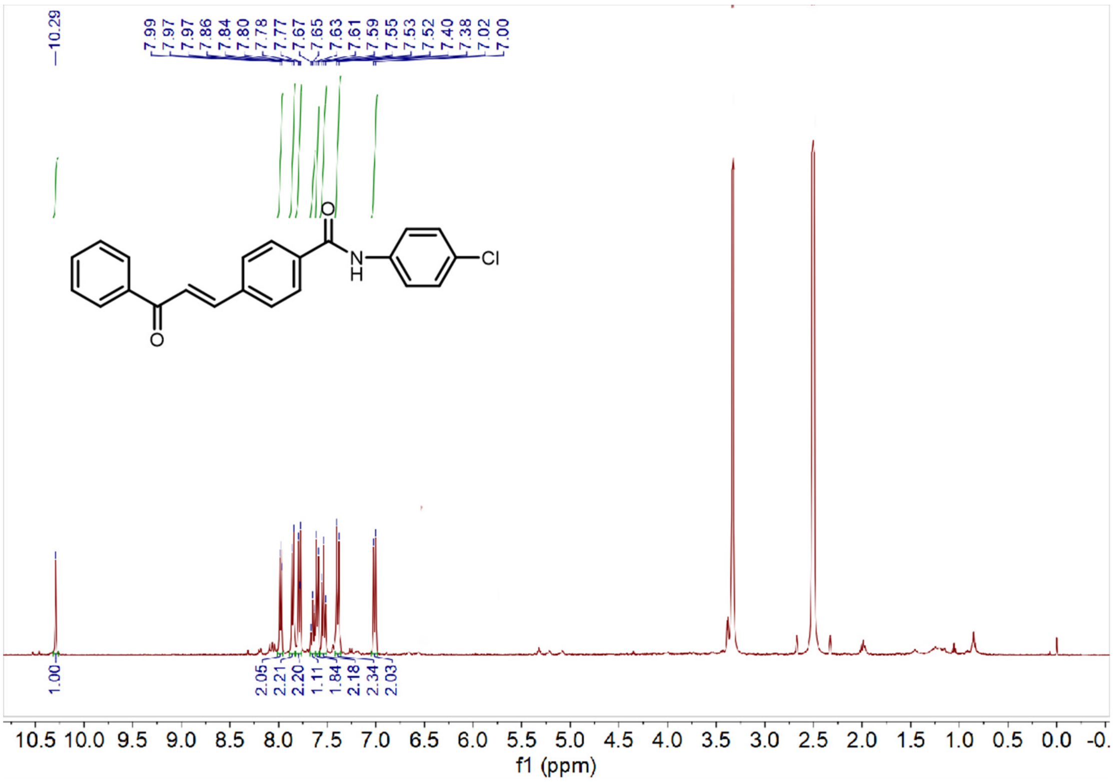

Figure S65 ^{1}H-NMR（**B2**）

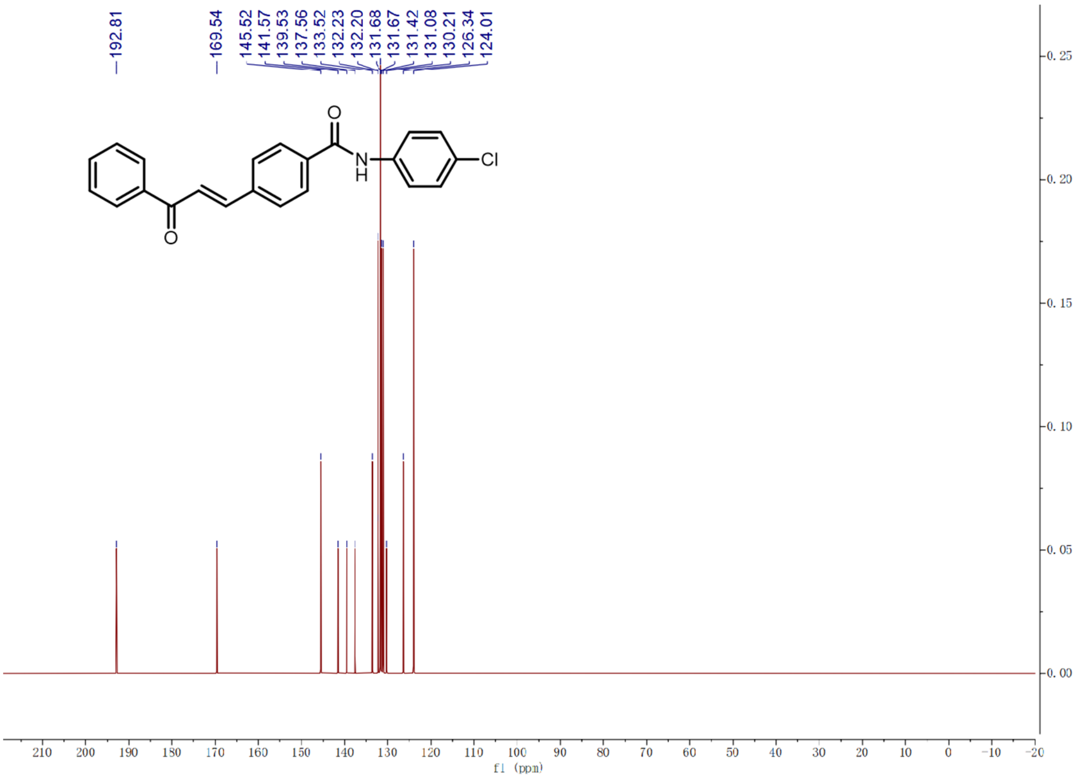

Figure S66 ^{13}C-NMR (**B2**)

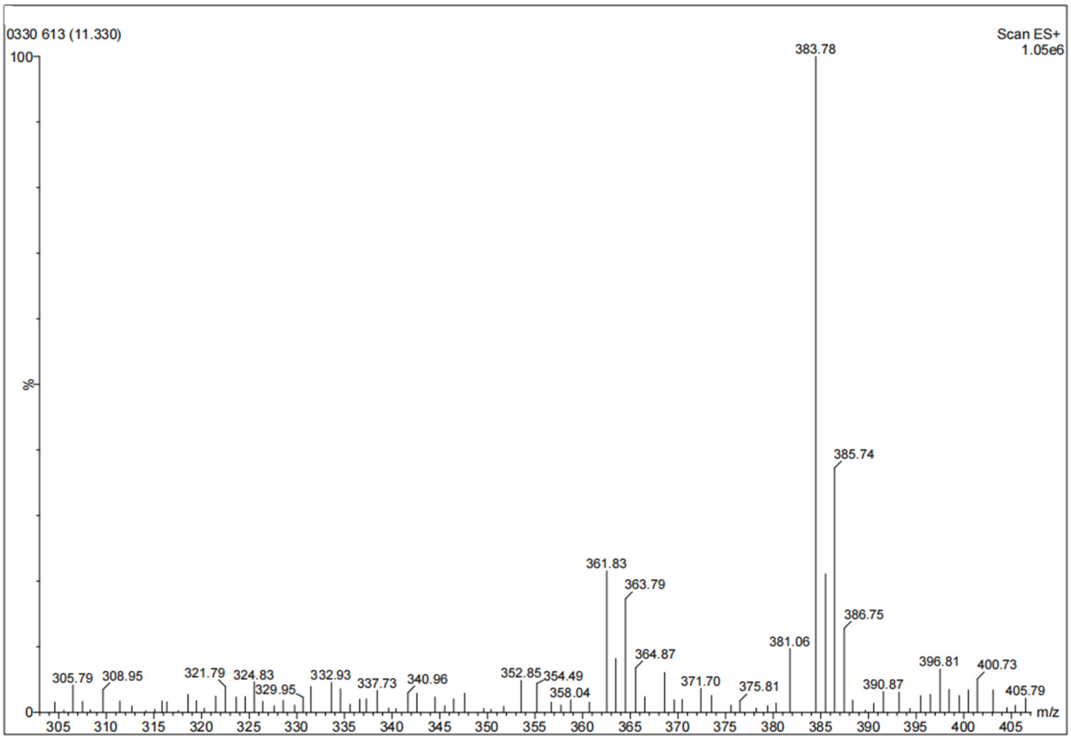

Figure S67 MS (**B2**)

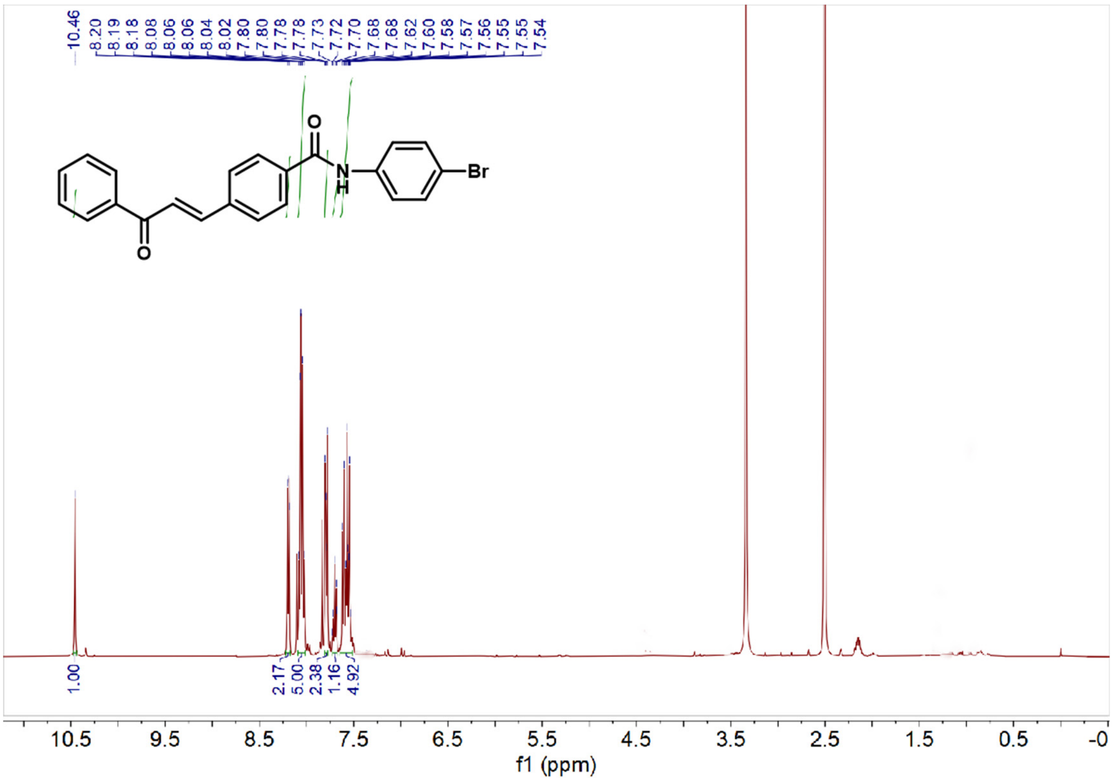

Figure S68 ^{1}H-NMR（**B3**）

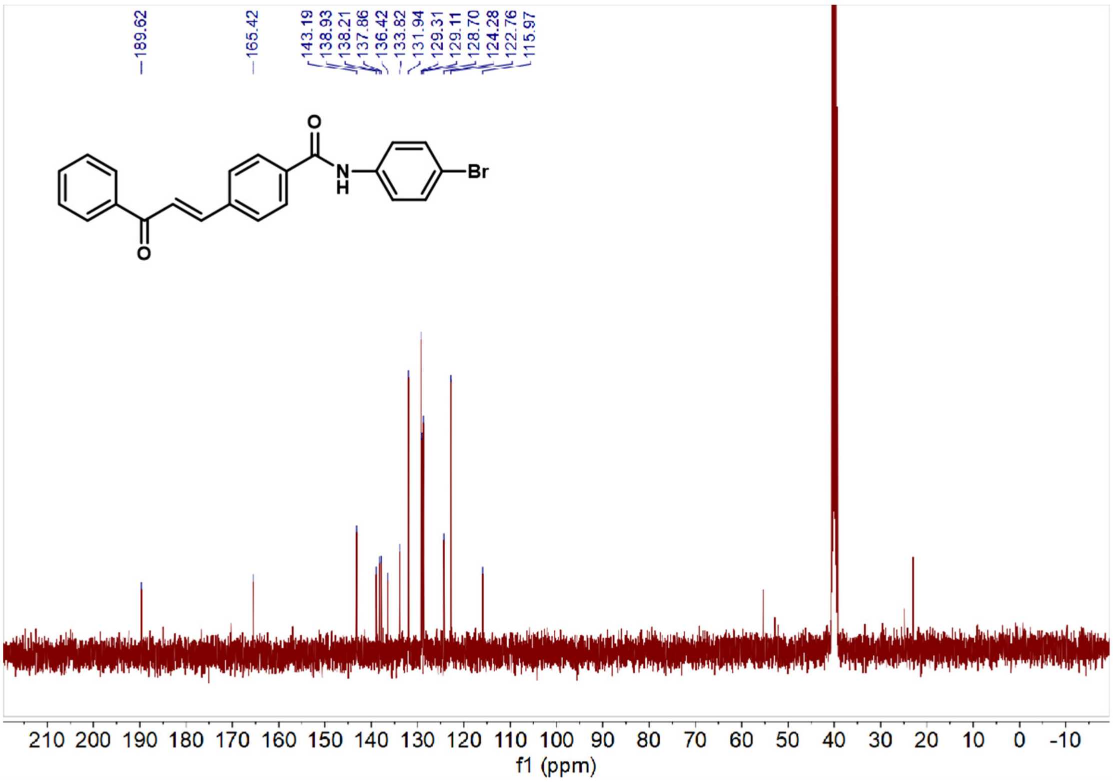

Figure S69 ^{13}C-NMR（**B3**）

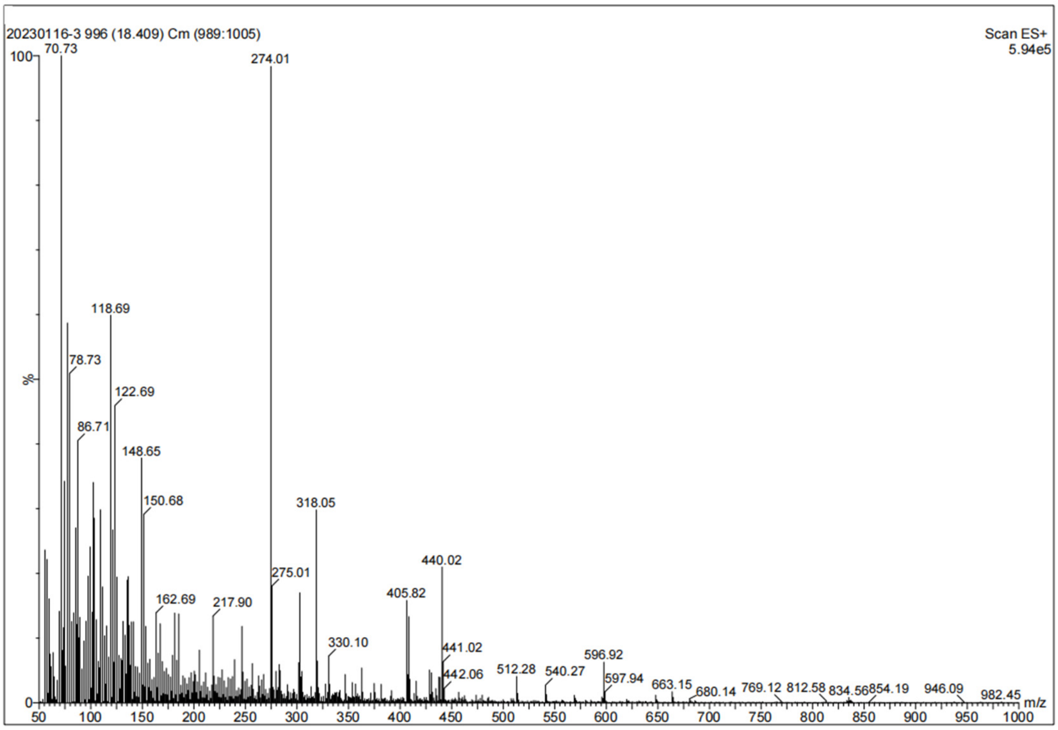

Figure S70 MS（**B3**）

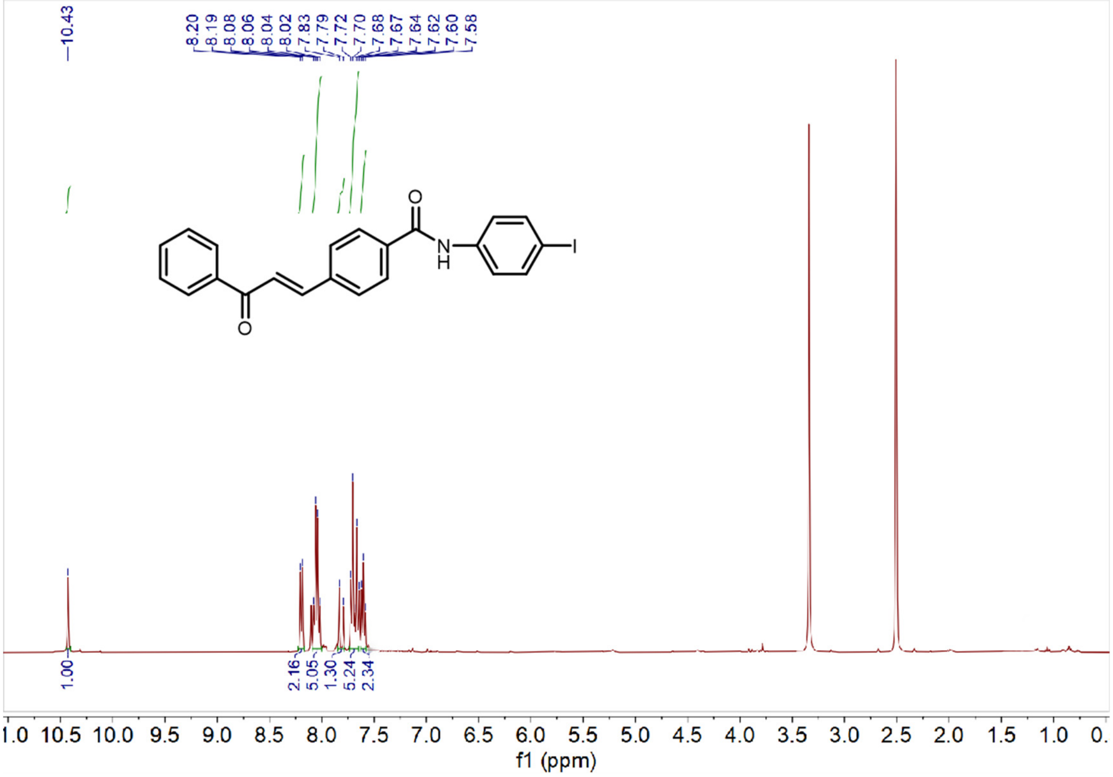

Figure S71 ^{1}H-NMR（**B4**）

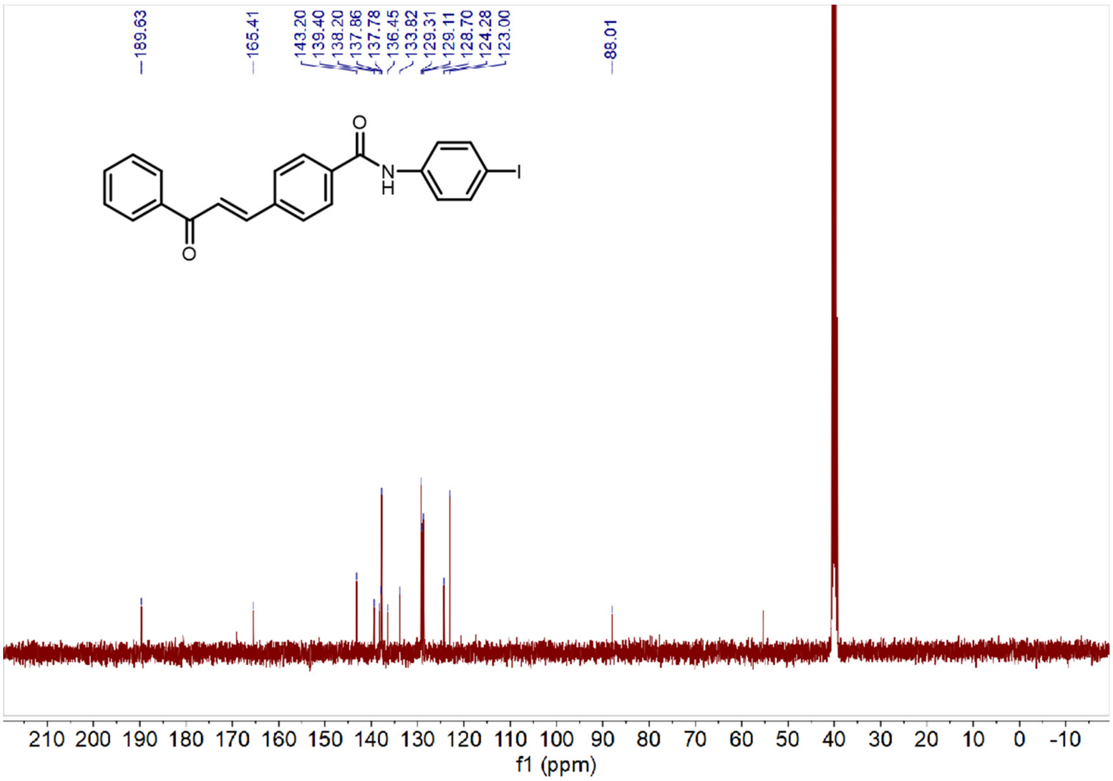

Figure S72 ^{13}C-NMR（**B4**）

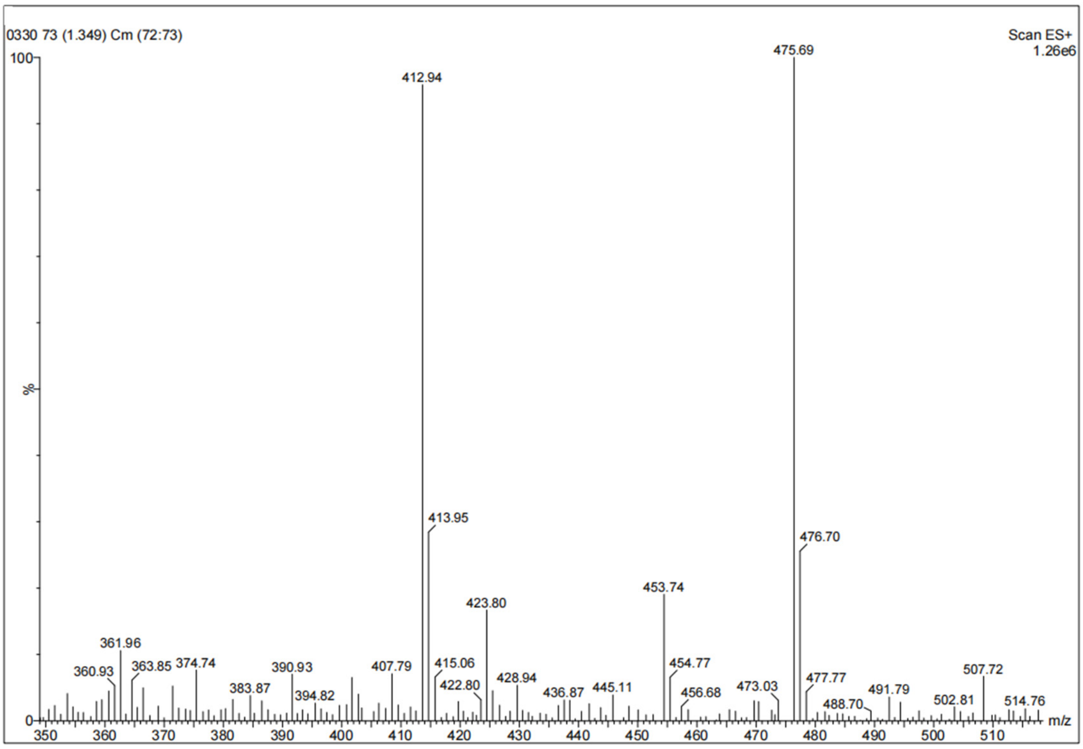

Figure S73 MS (**B4**)

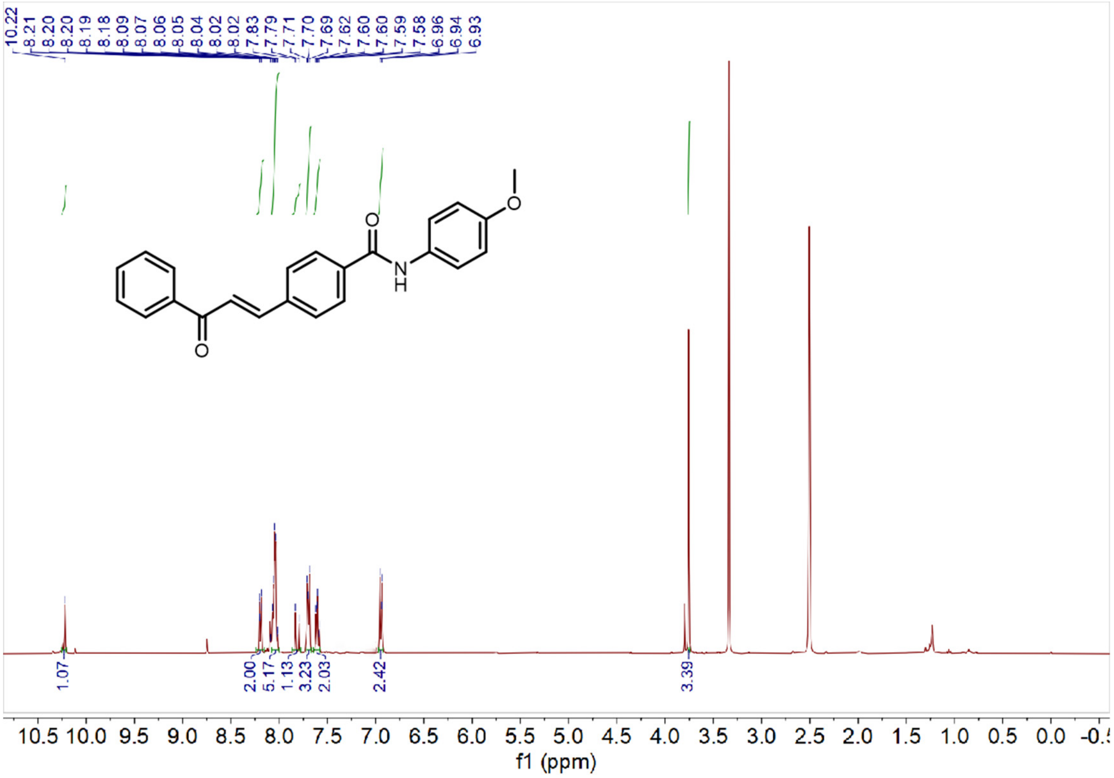

Figure S74 ^{1}H-NMR（**B5**）

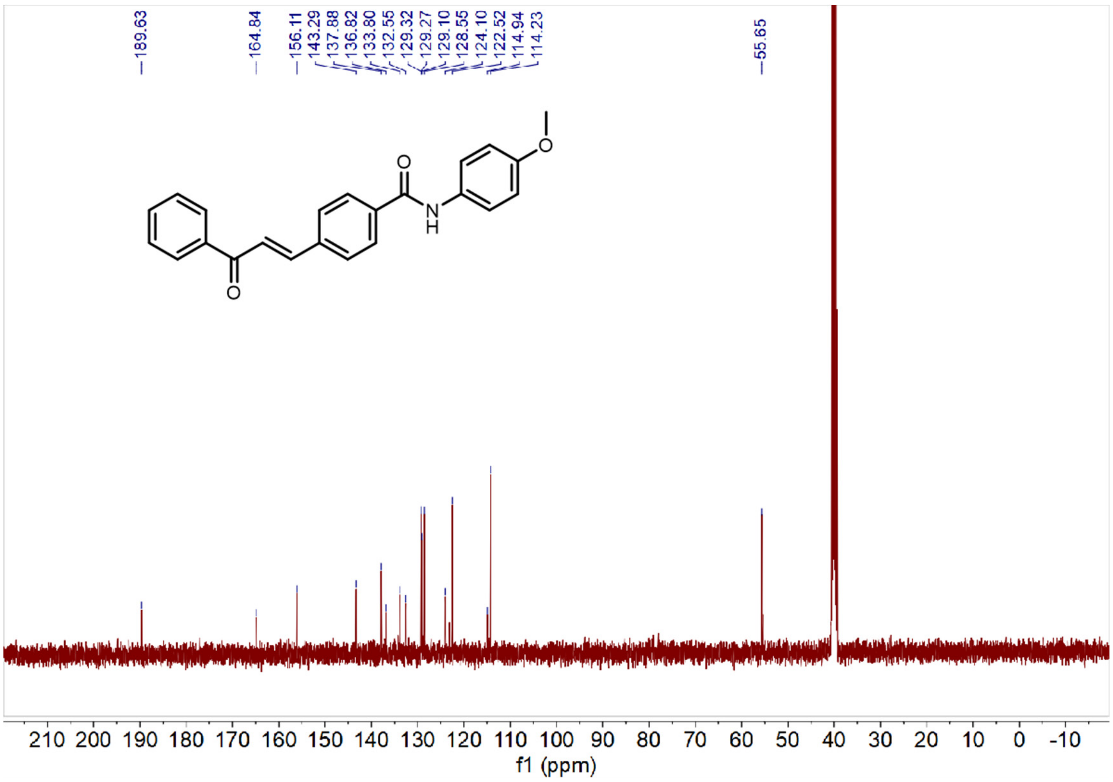

Figure S75 ^{13}C-NMR（**B5**）

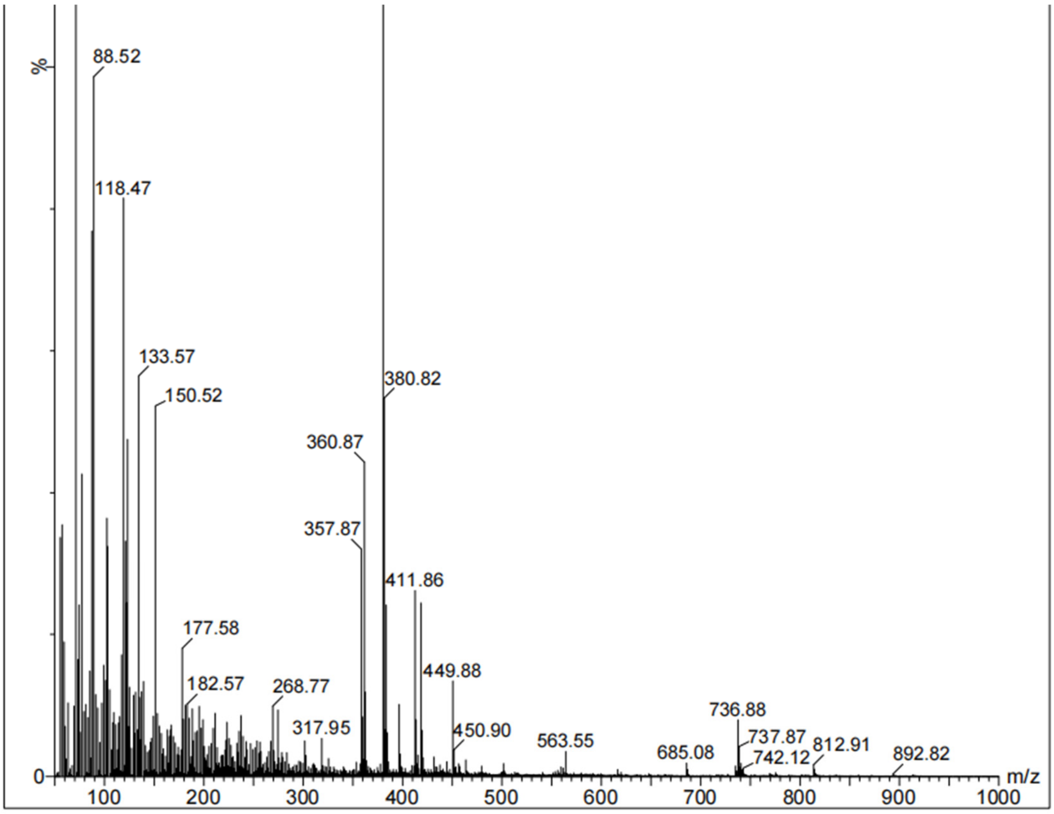

Figure S76 MS（**B5**）

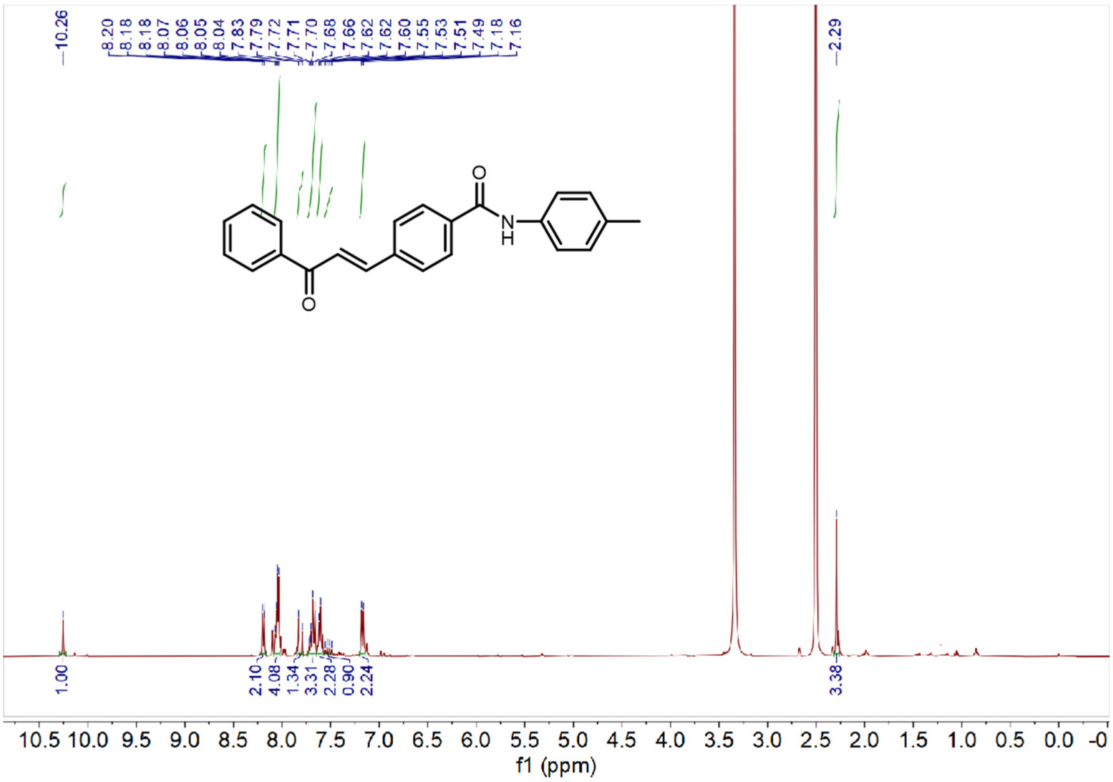

Figure S77 ¹H-NMR（**B6**）

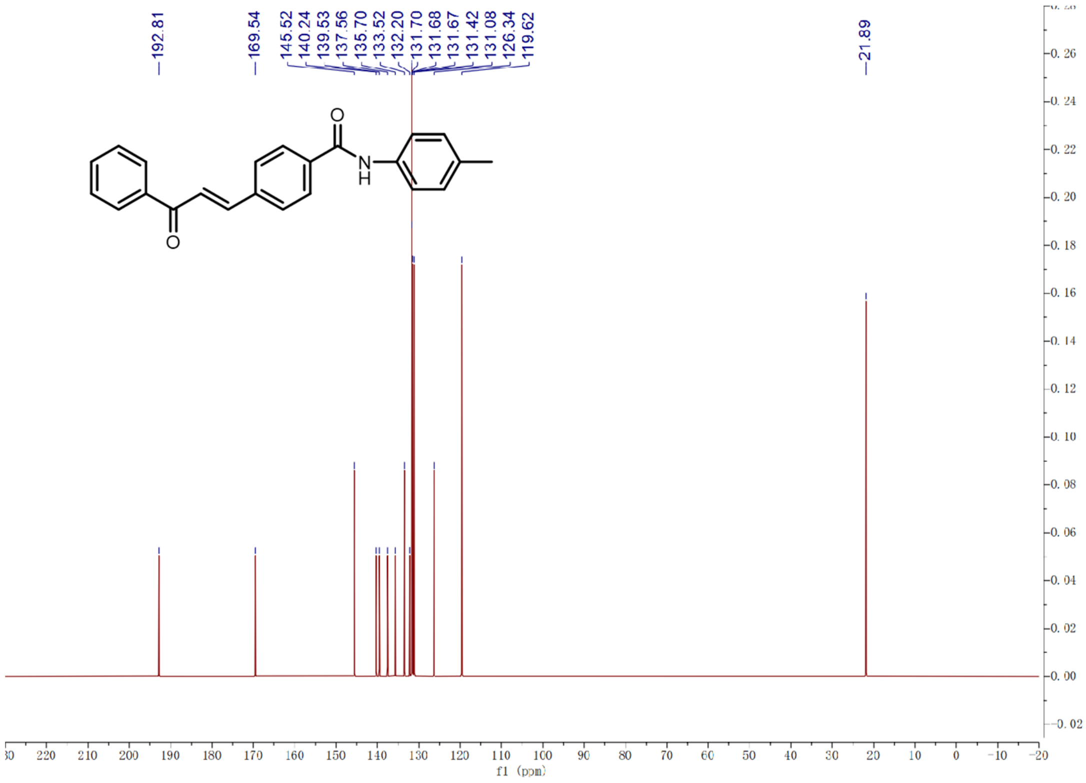

Figure S78 ^{13}C-NMR（**B6**）

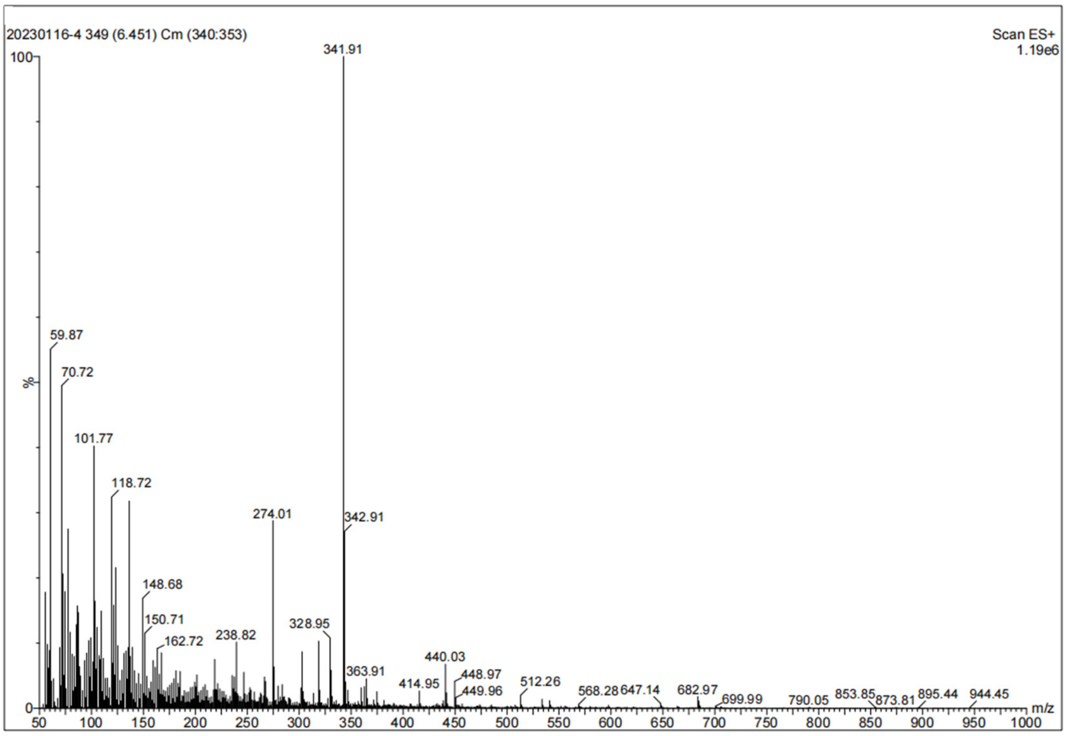

Figure S79 MS（**B6**）

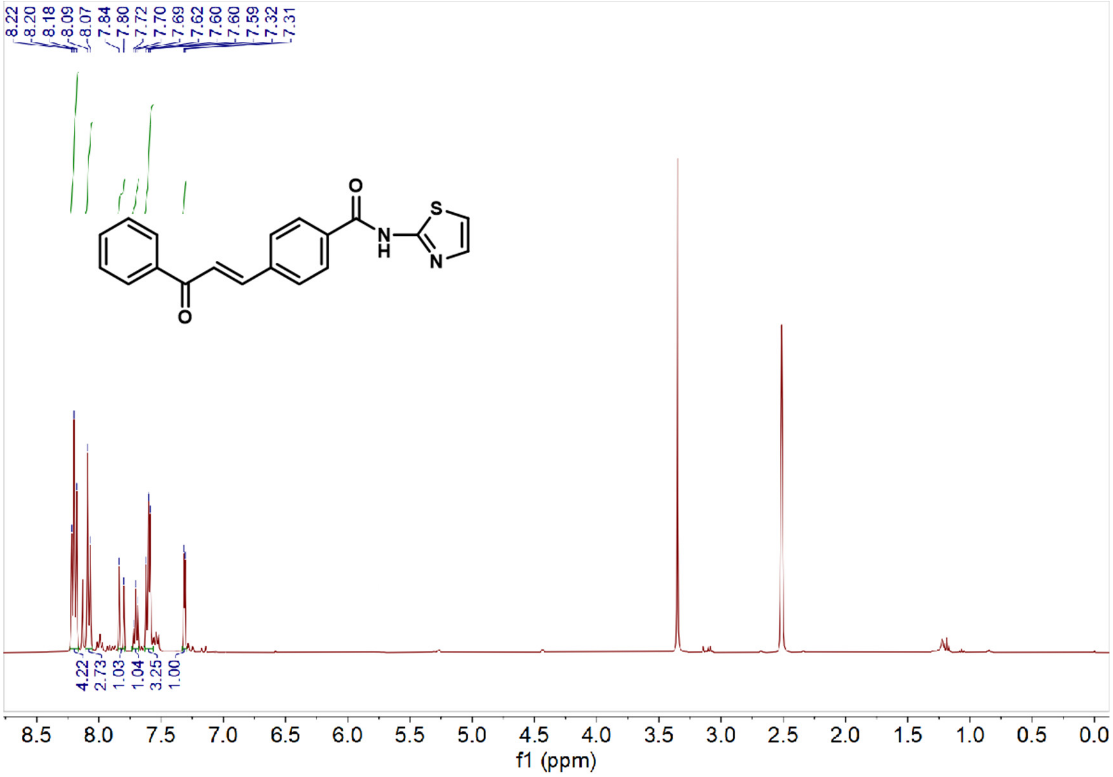

Figure S80 ^{1}H-NMR（**B7**）

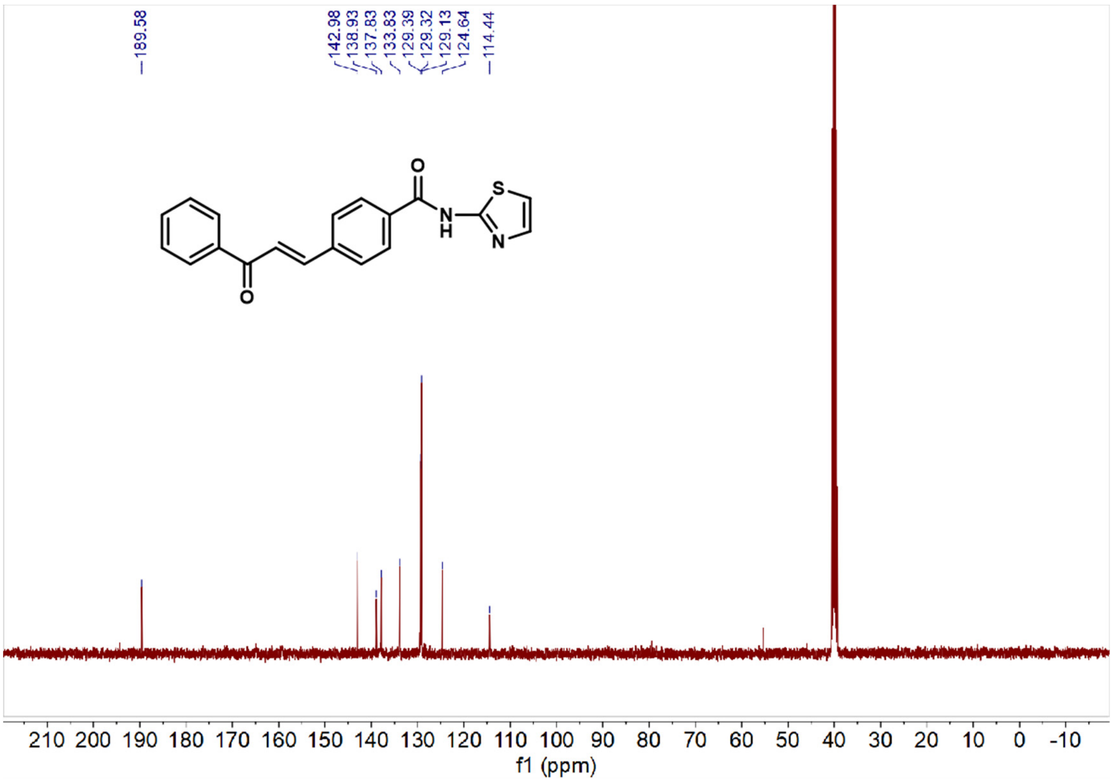

Figure S81 ^{13}C-NMR（**B7**）

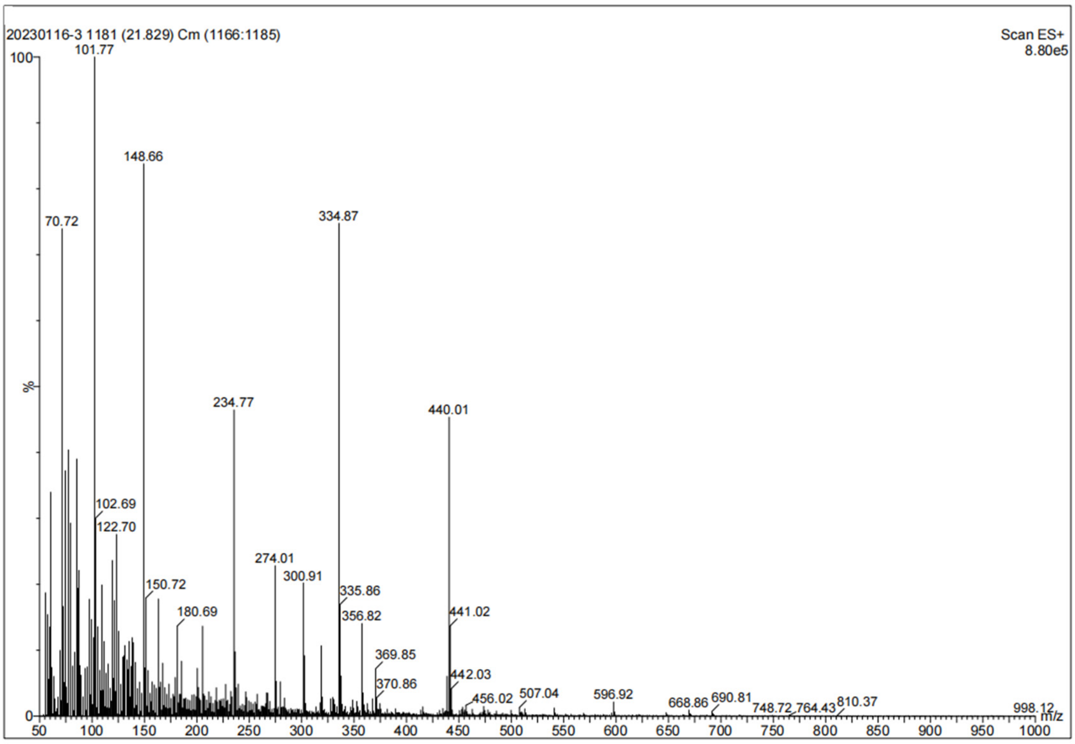

Figure S82 MS（**B7**）

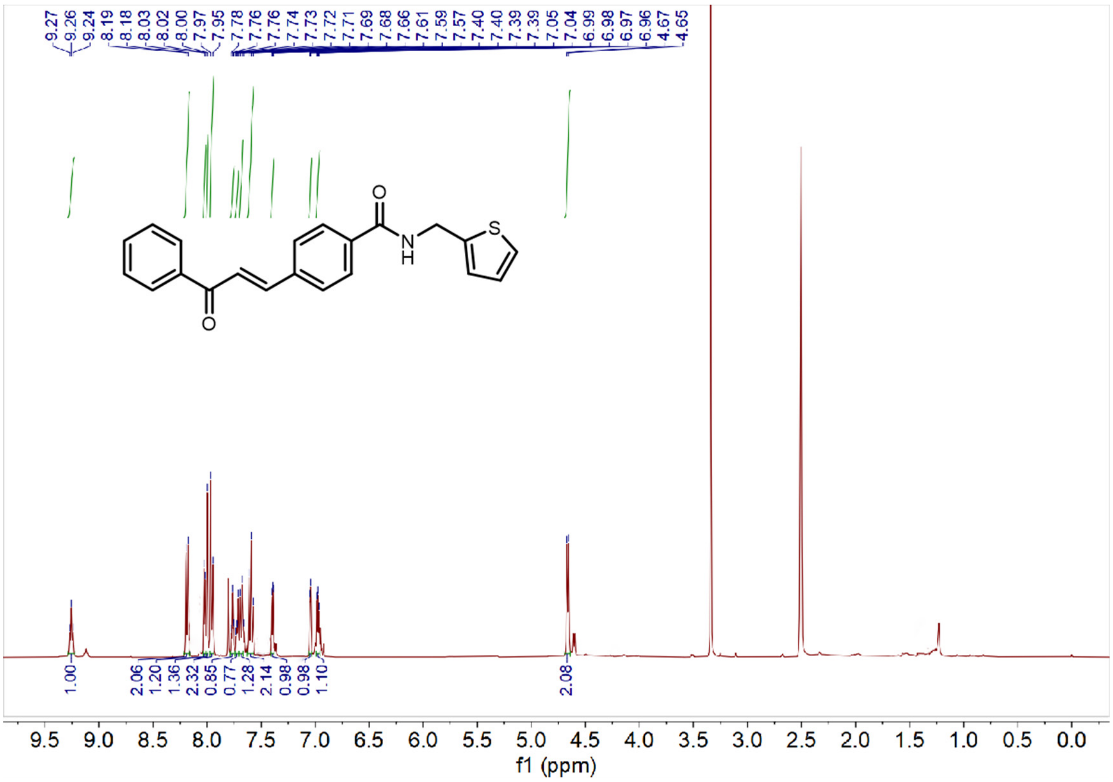

Figure S83 ^{1}H-NMR（**B8**）

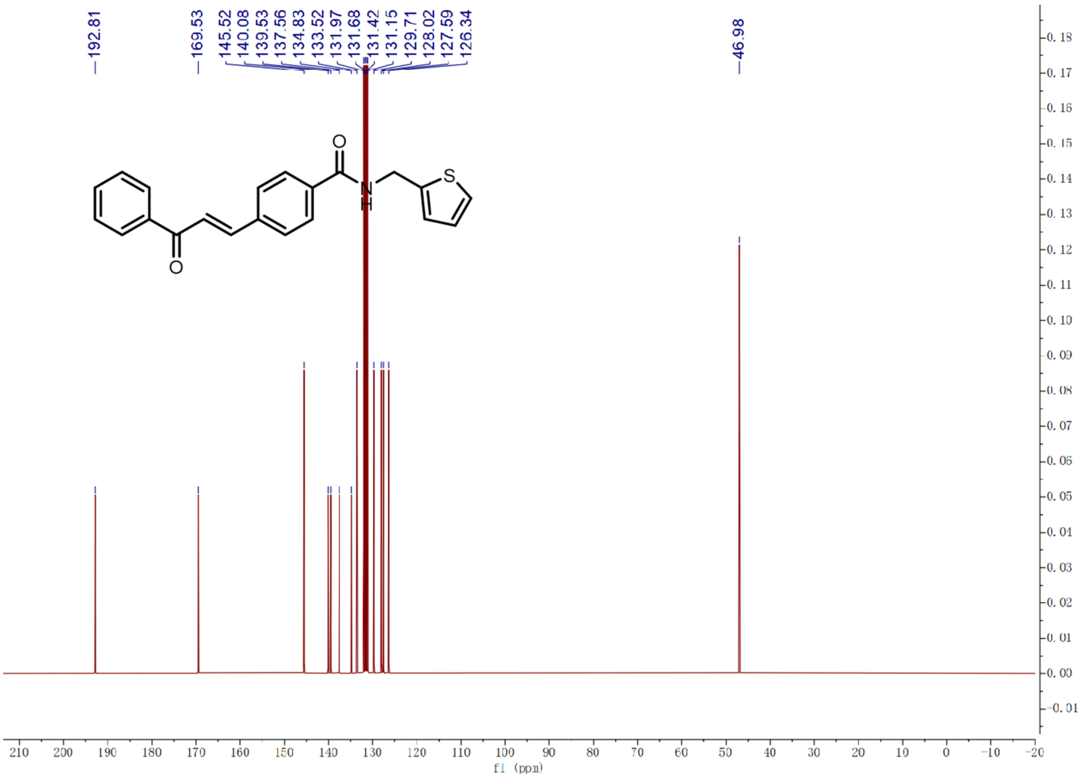

Figure S84 ^{13}C-NMR（**B8**）

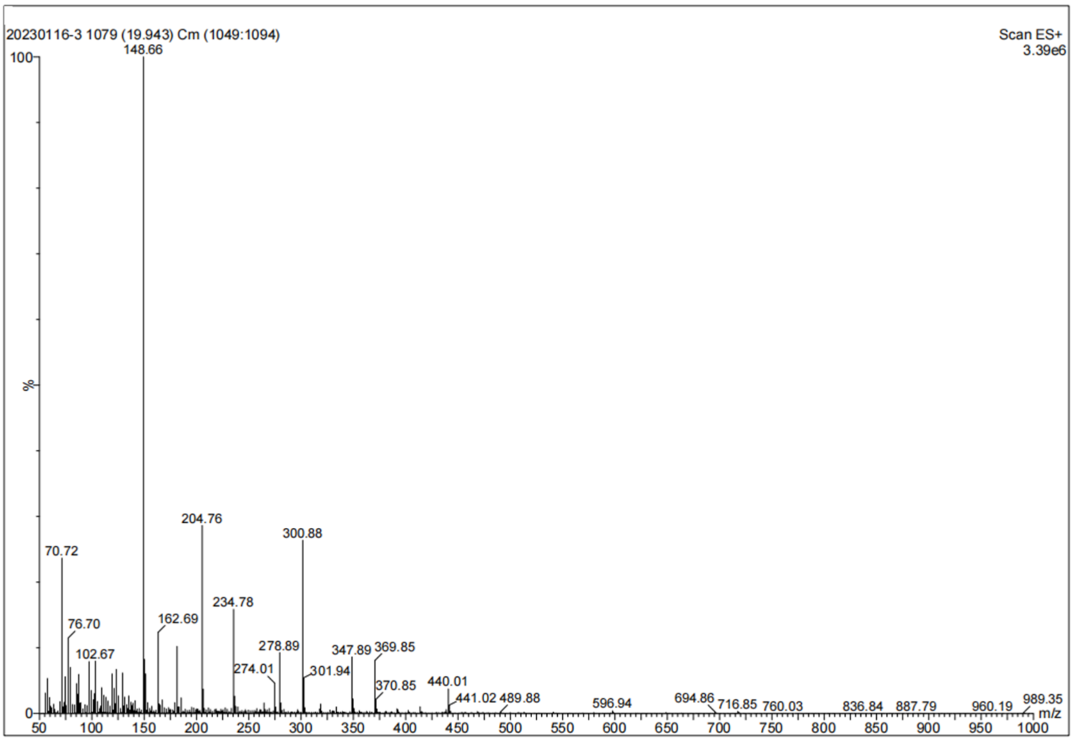

Figure S85 MS（**B8**）

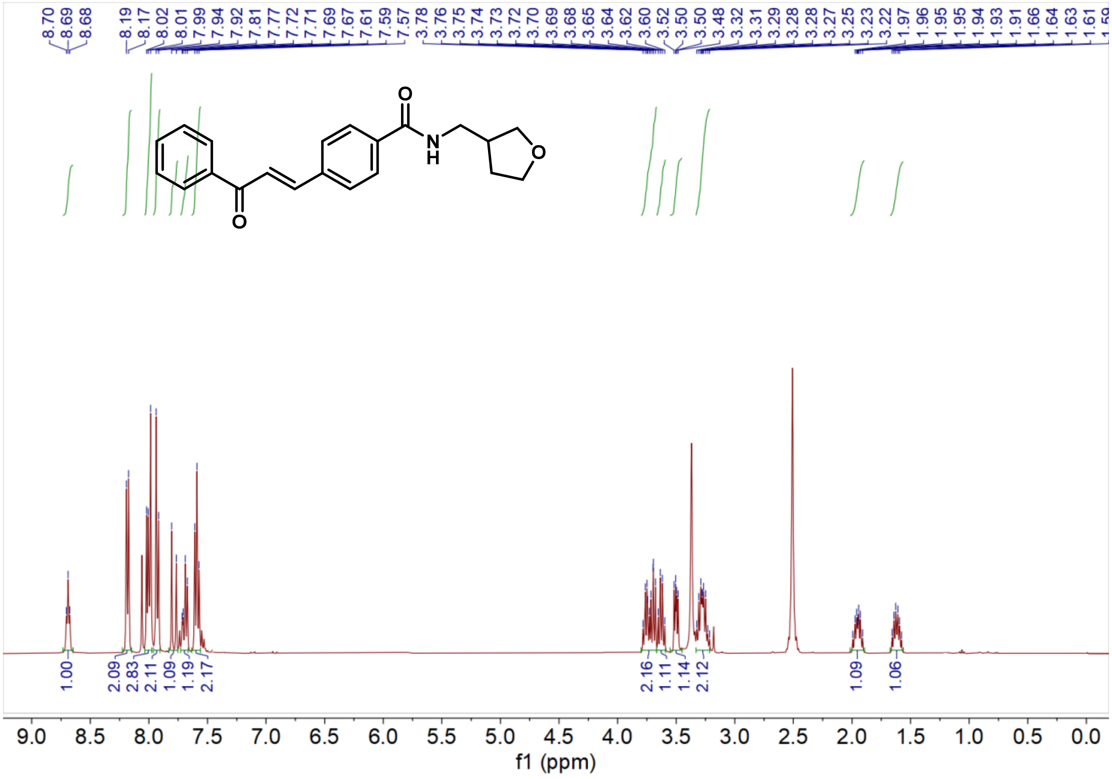

Figure S86 ^{1}H-NMR（**B9**）

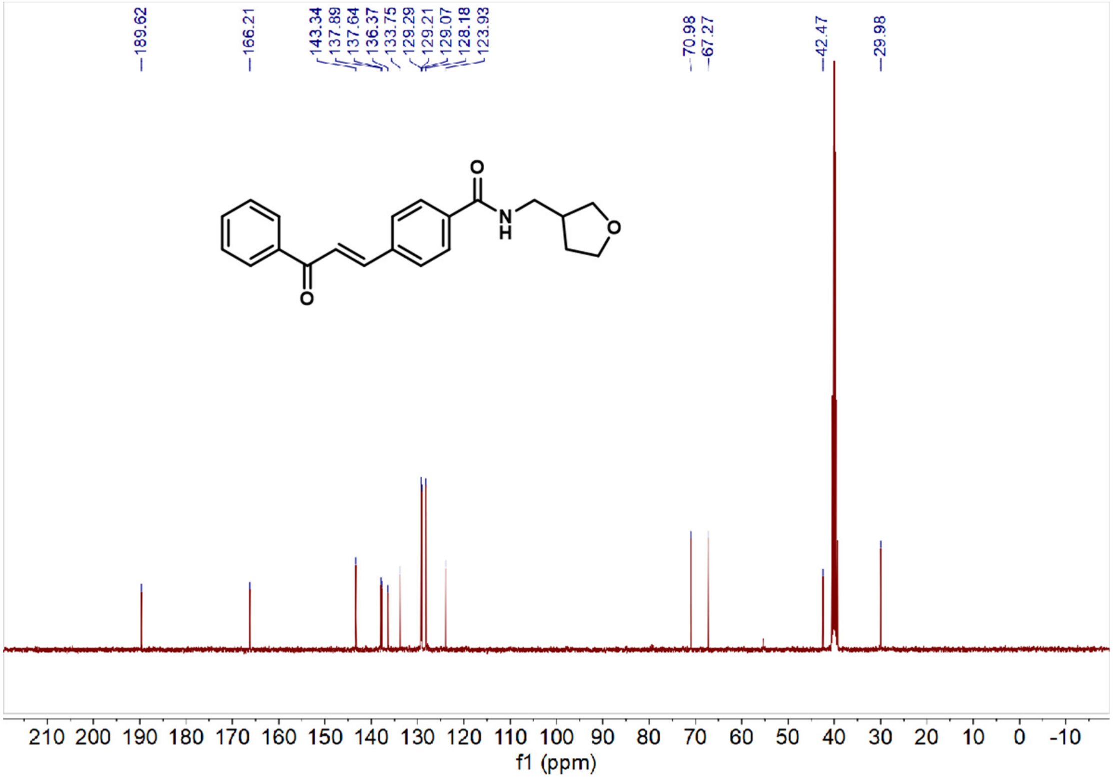

Figure S87 ^{13}C-NMR（**B9**）

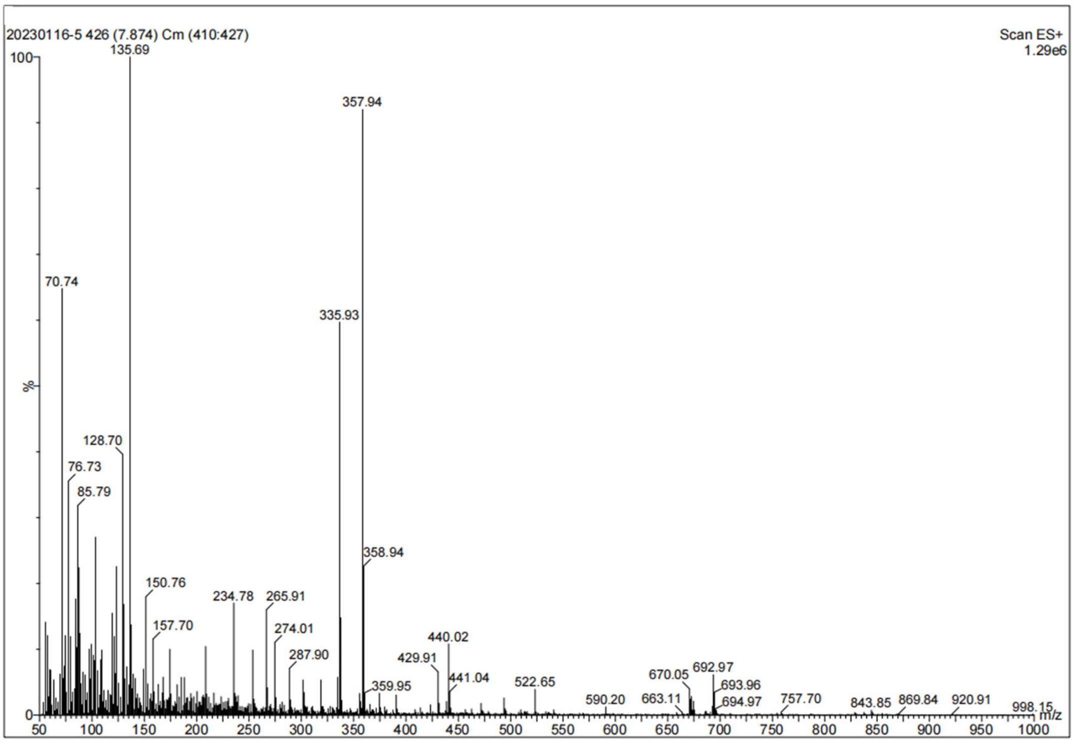

Figure S88 MS（**B9**）

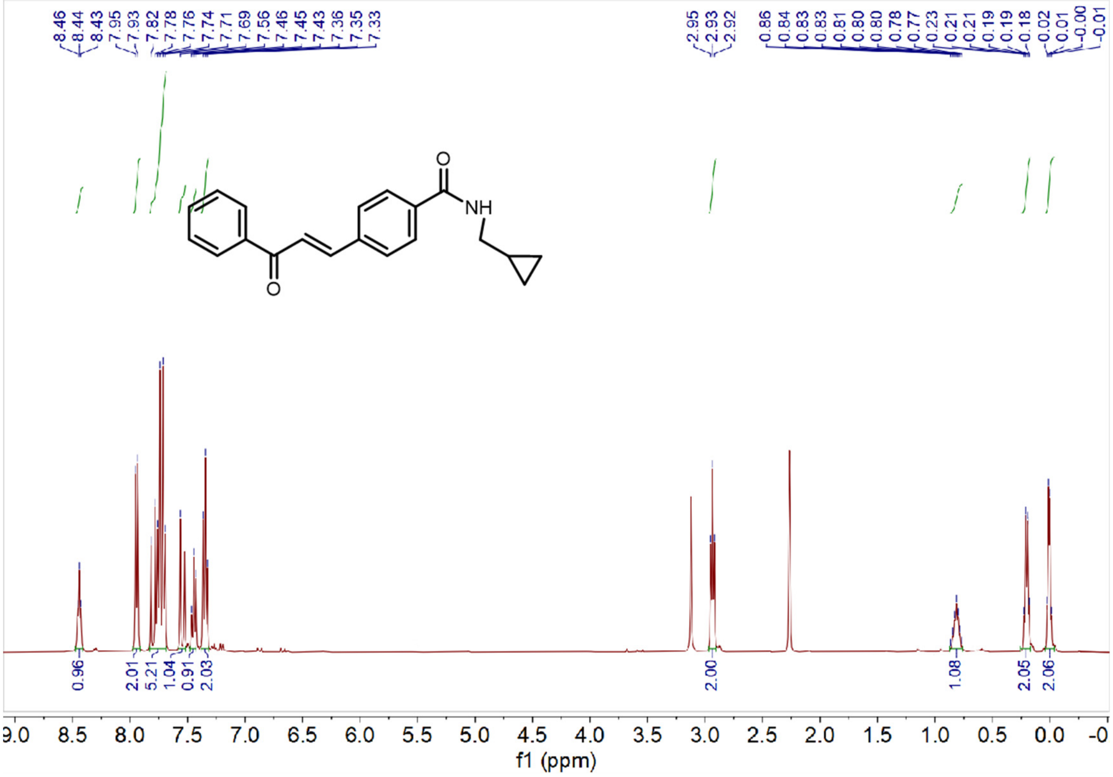

Figure S89 ^{1}H-NMR（**B10**）

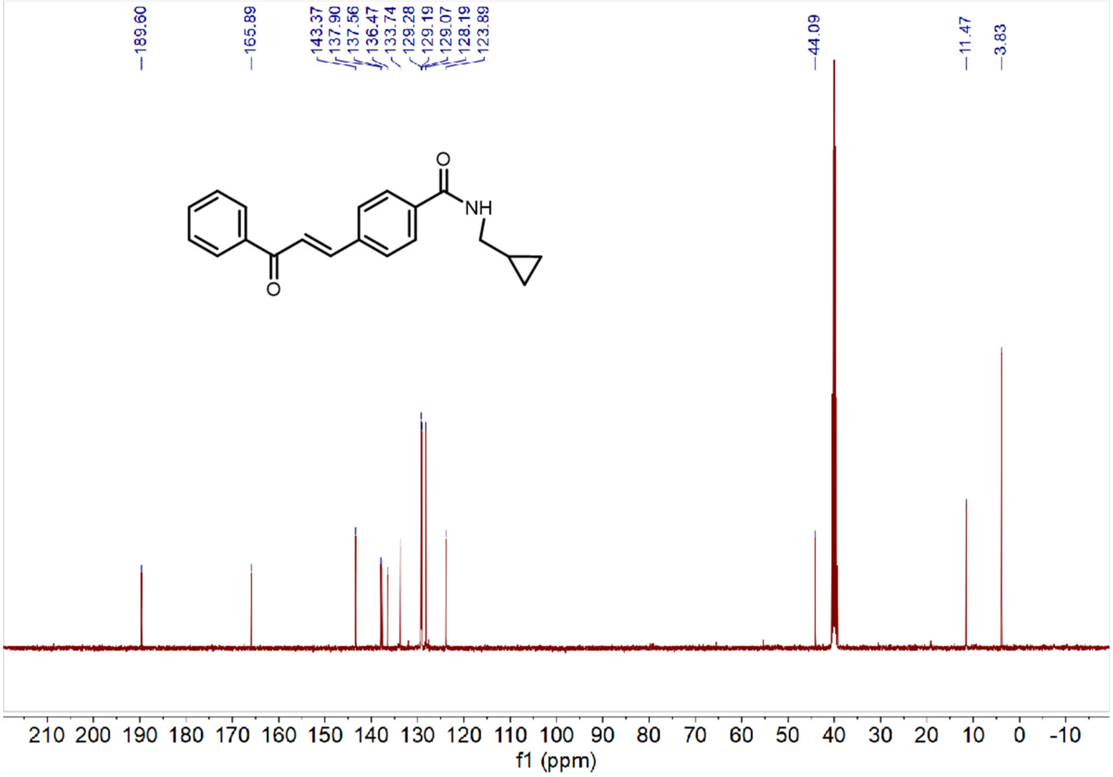

Figure S90 ^{13}C-NMR（**B10**）

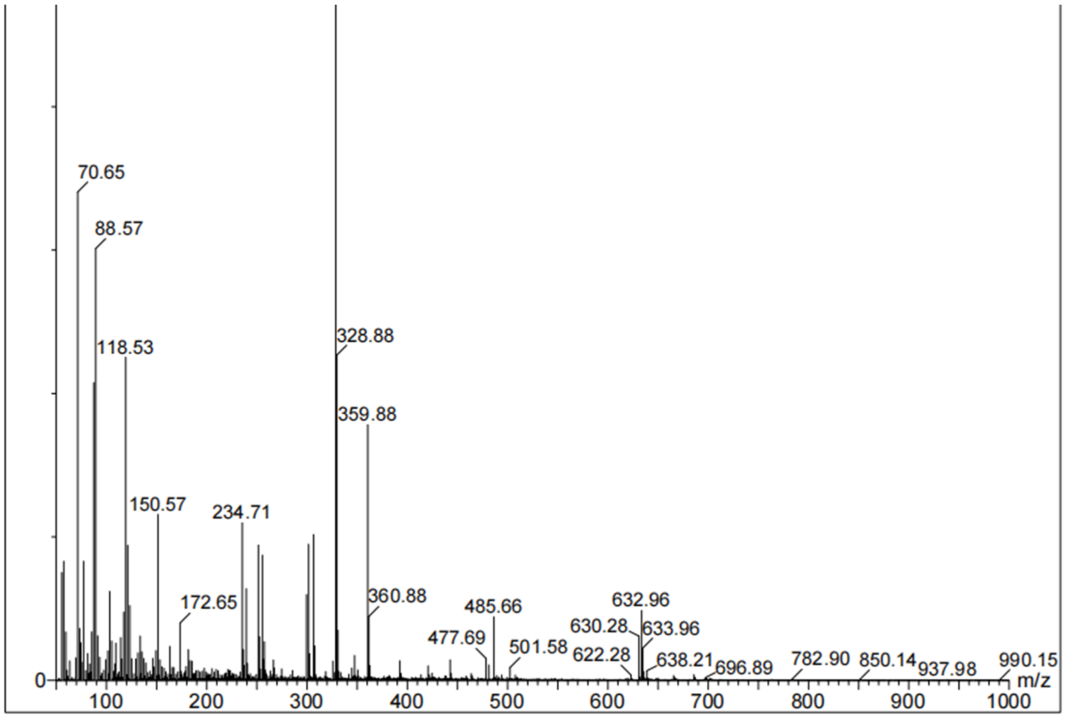

Figure S91 MS（**B10**）

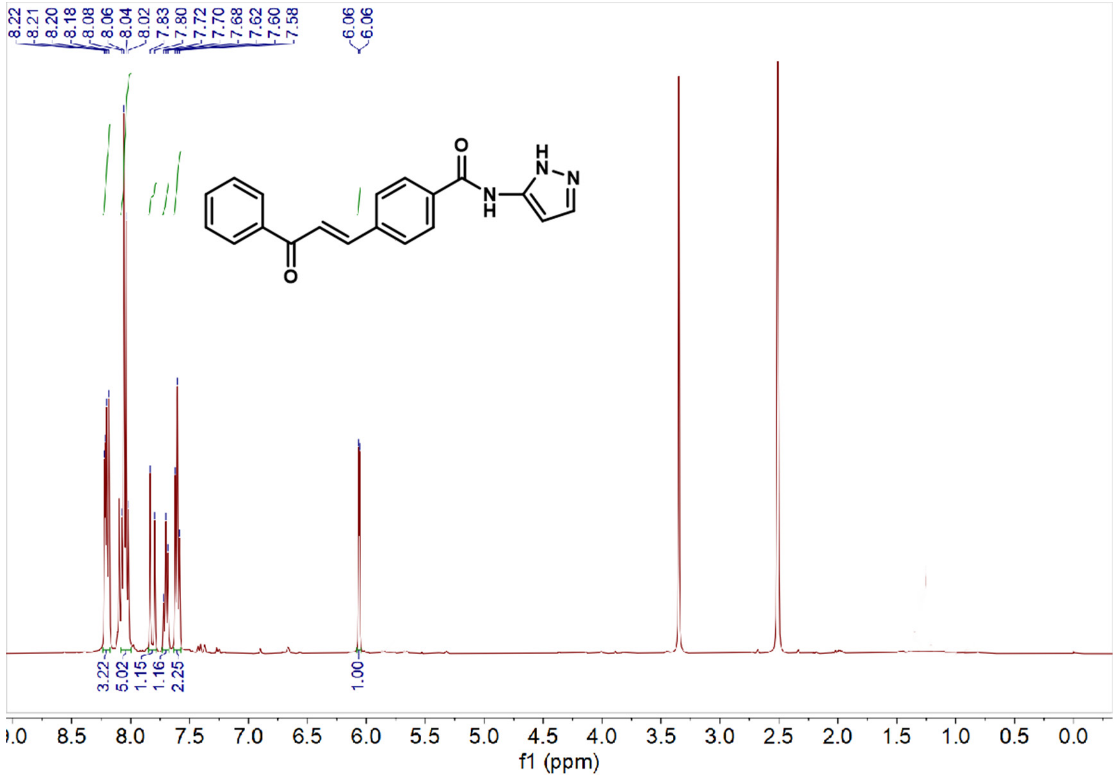

Figure S92 ^{1}H-NMR（**B11**）

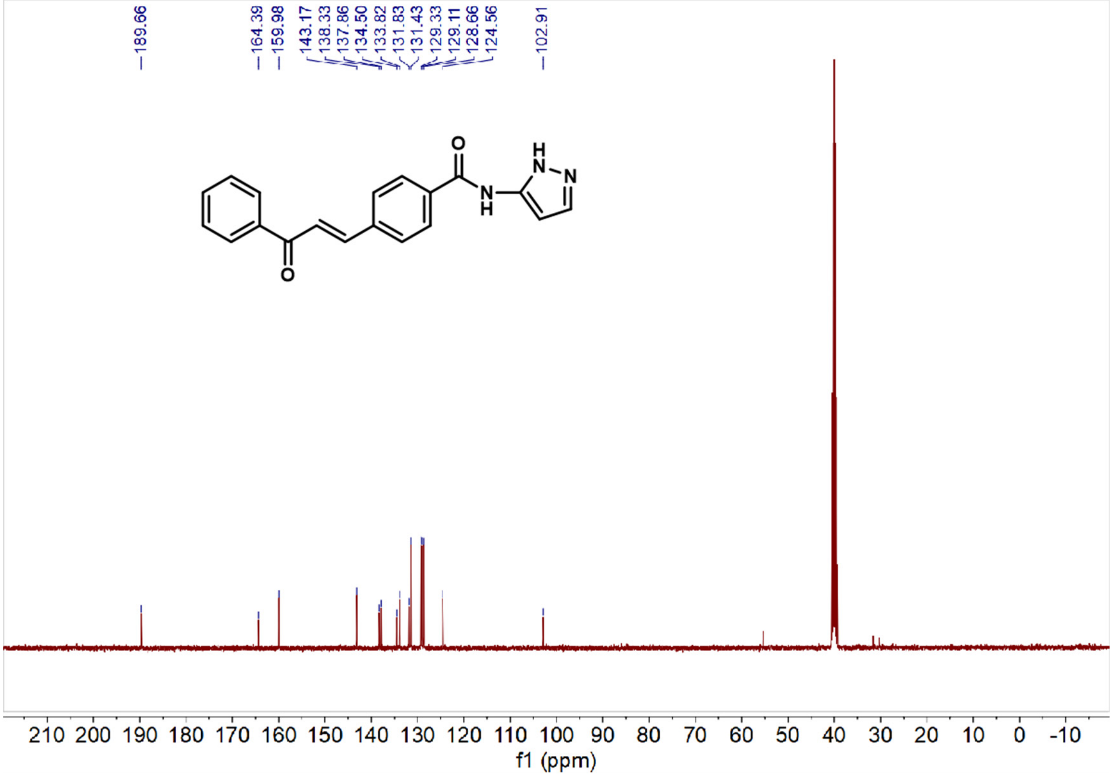

Figure S93 ^{13}C-NMR（**B11**）

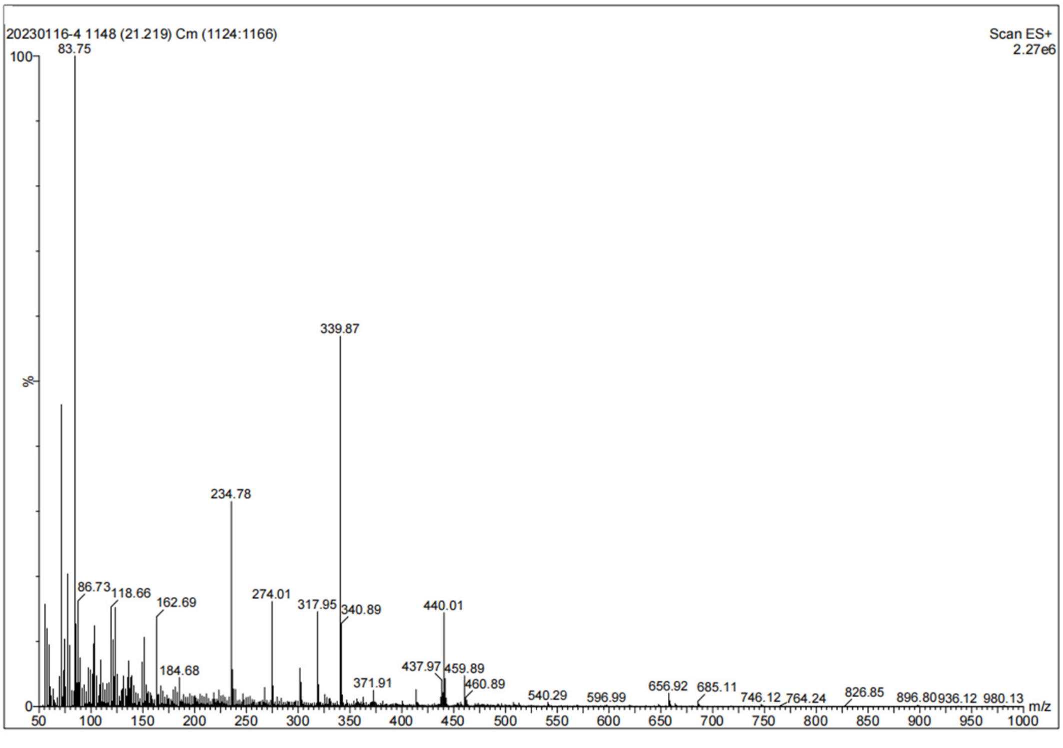

Figure S94 MS（**B11**）

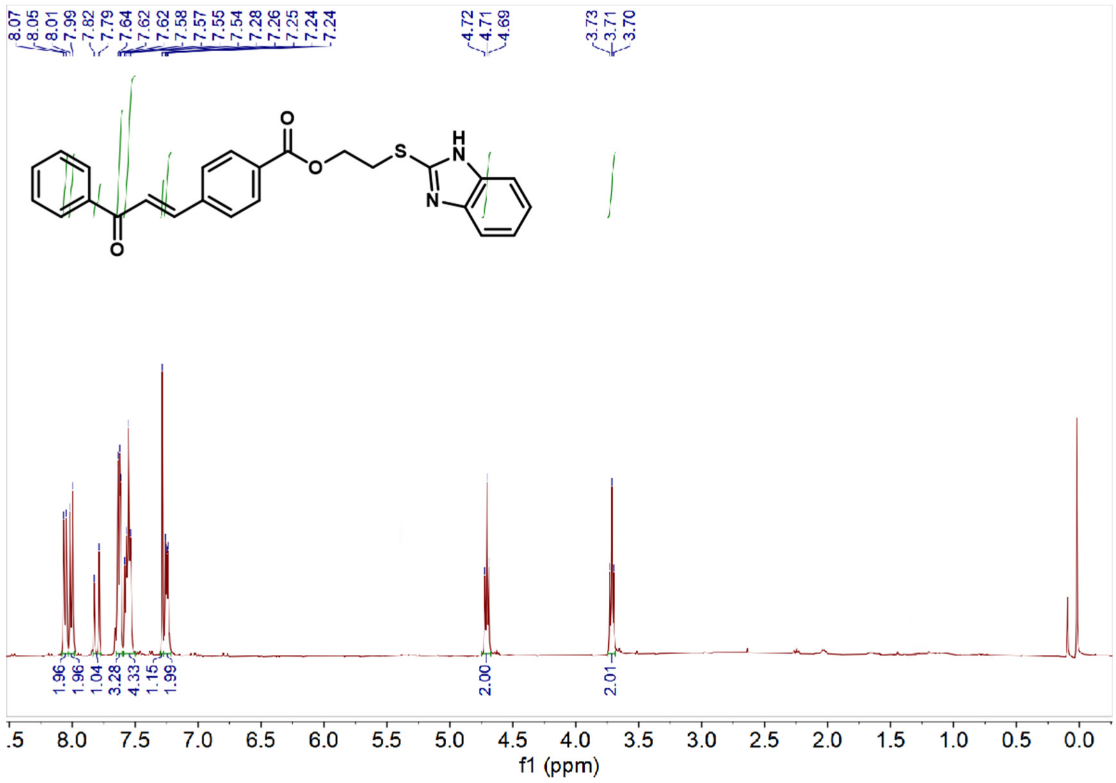

Figure S95 ^{1}H-NMR（**B12**）

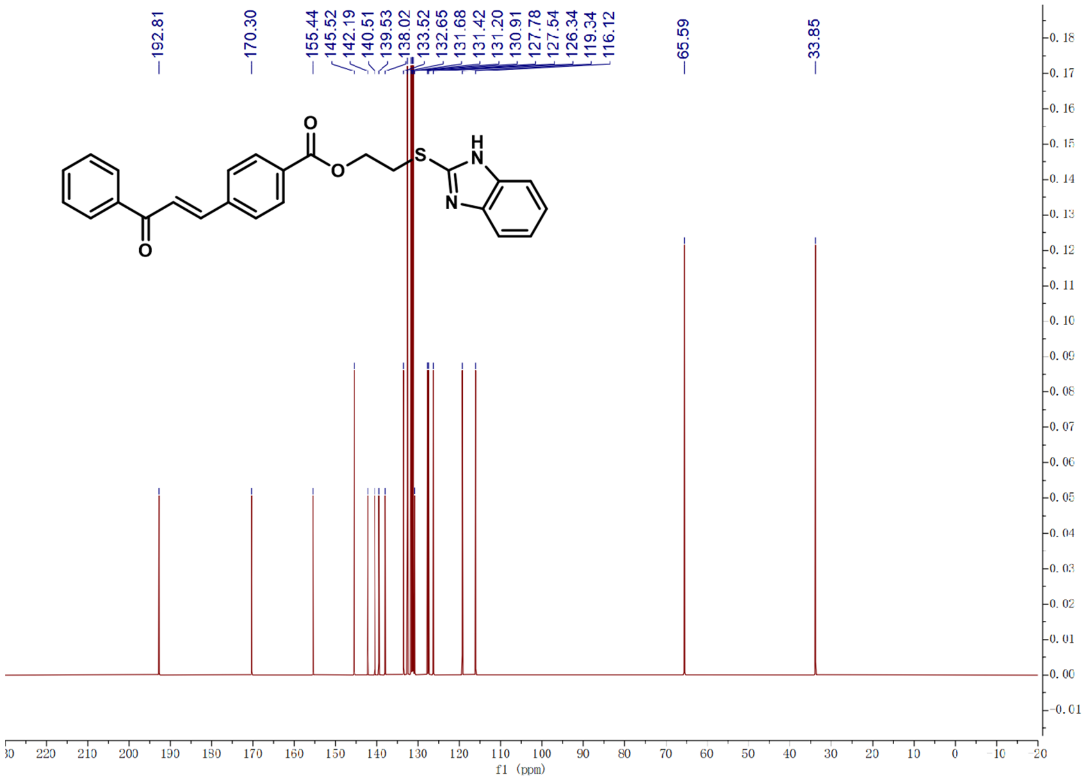

Figure S96 ^{13}C-NMR（**B12**）

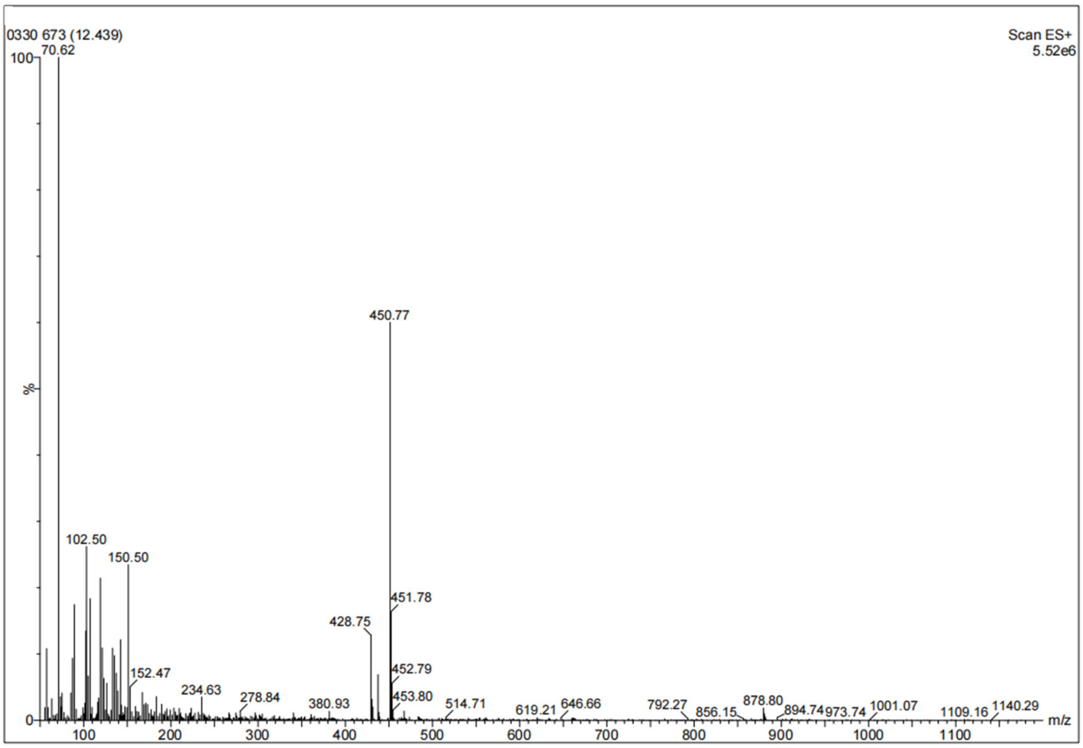

Figure S97 MS（**B12**）

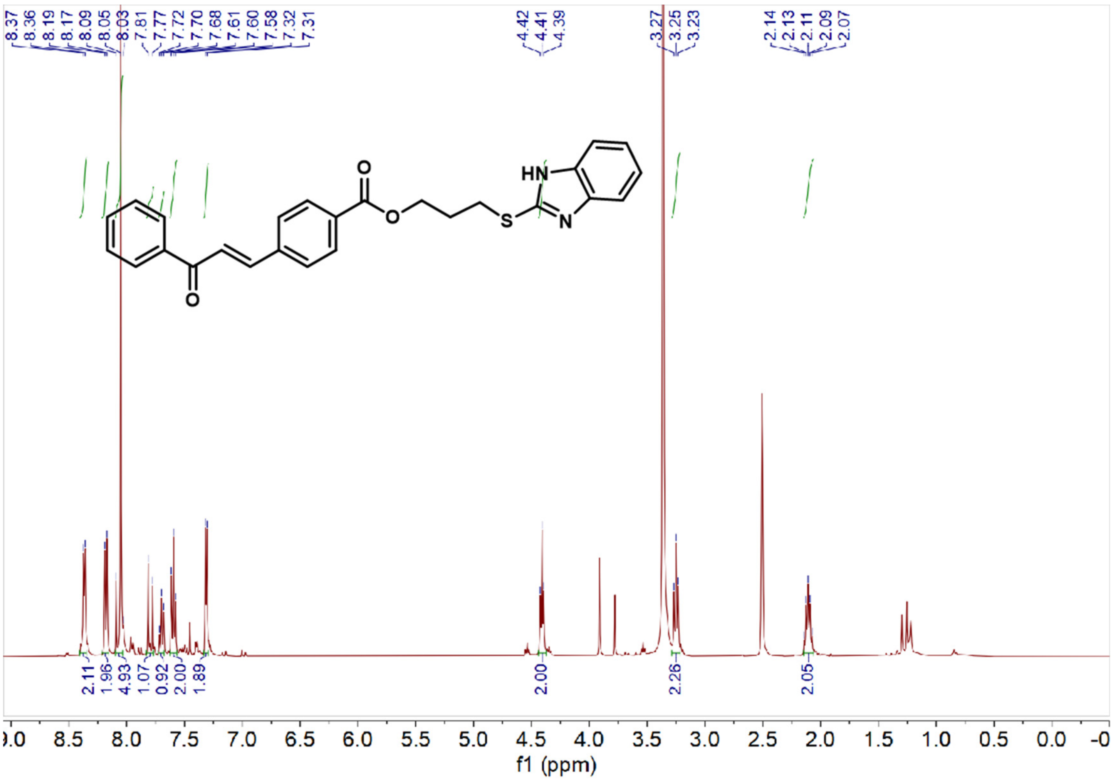

Figure S98 ^{1}H-NMR（**B13**）

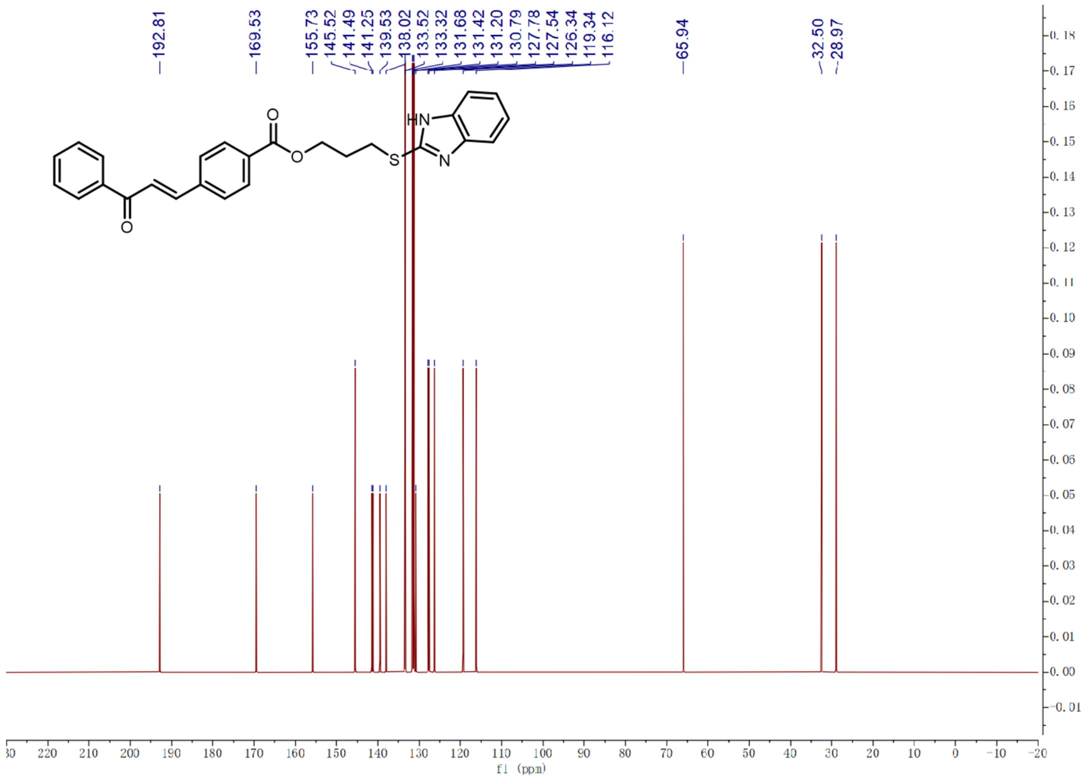

Figure S99 ^{13}C-NMR（**B13**）

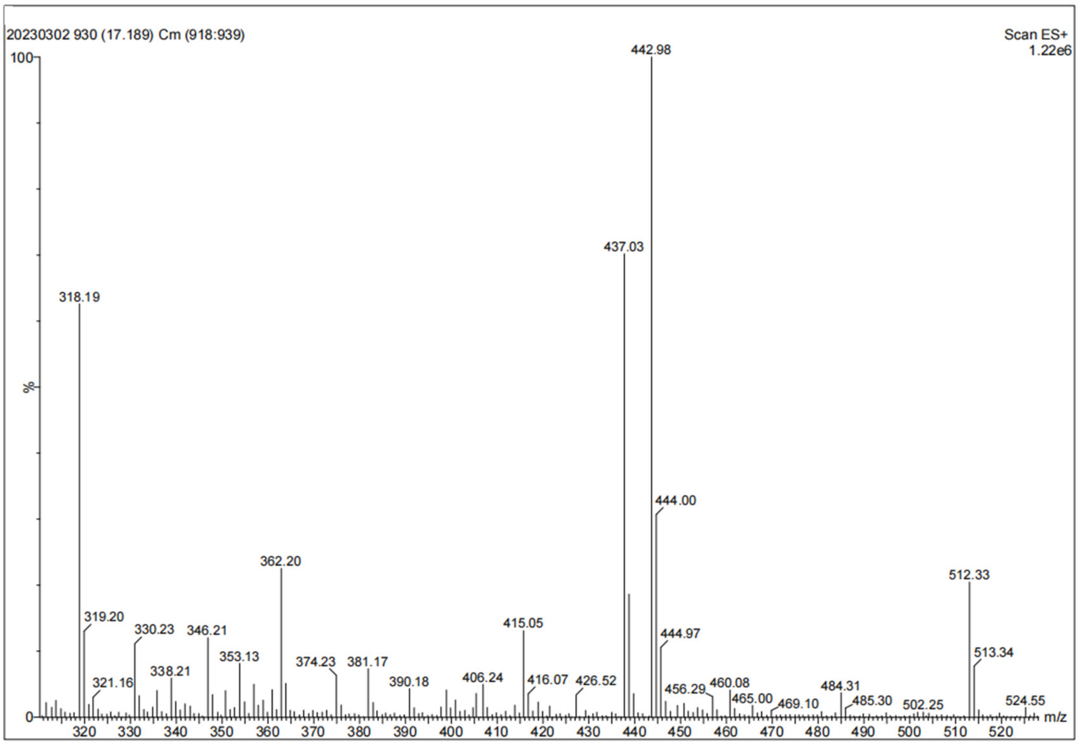

Figure S100 MS（**B13**）

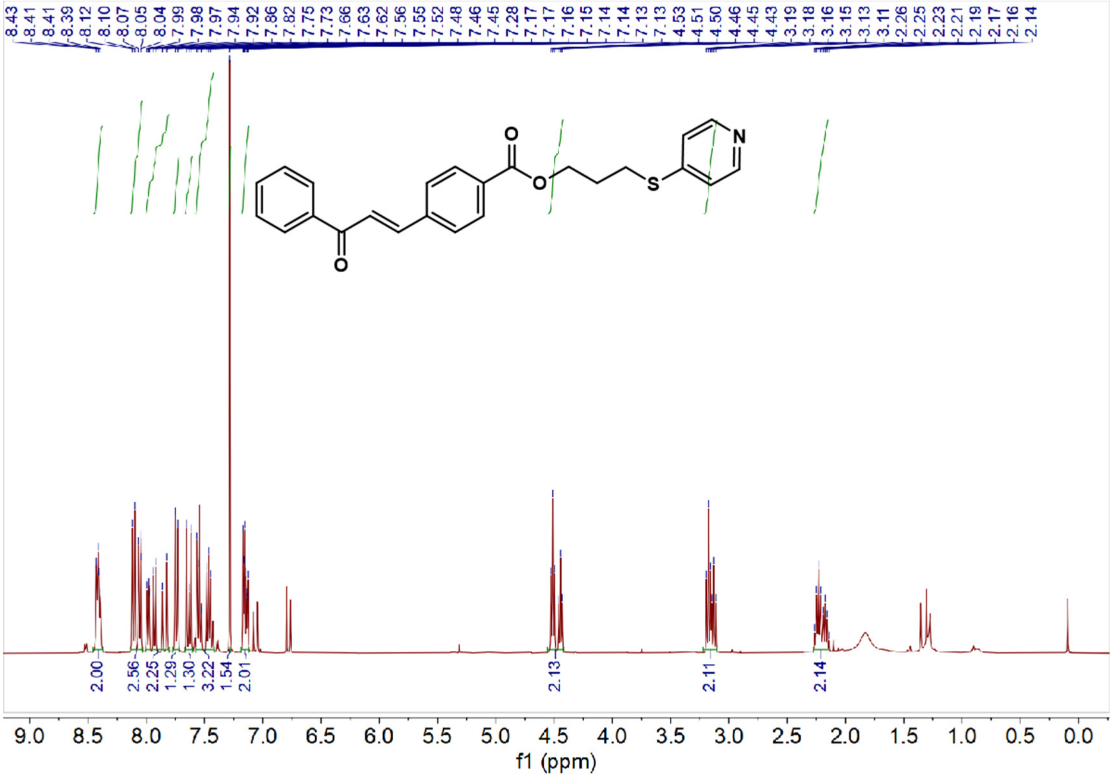

Figure S101 ^{1}H-NMR（**B14**)

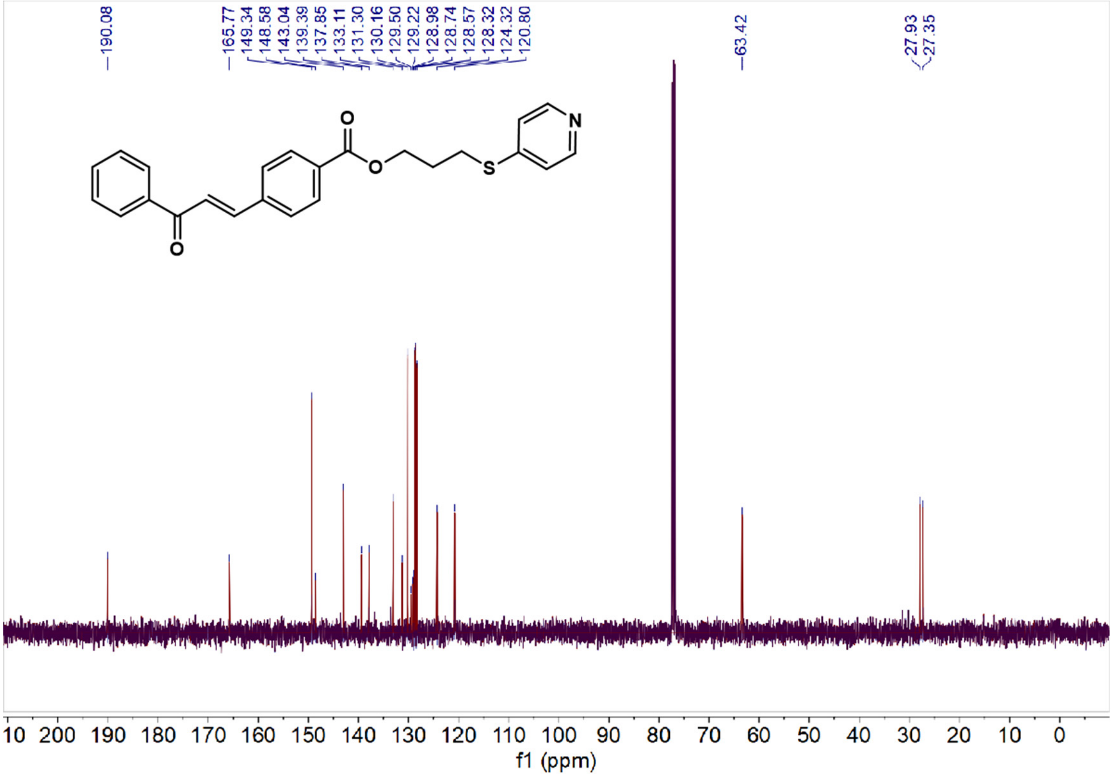

Figure S102 ^{13}C-NMR（**B14**）

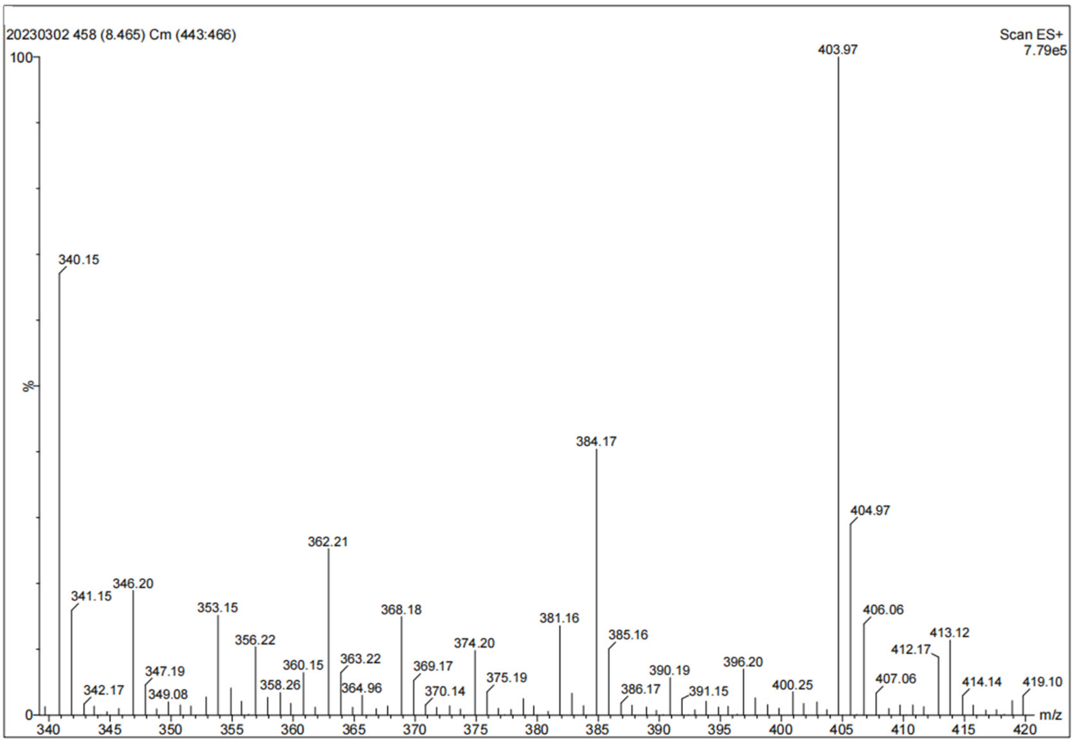

Figure S103 MS（**B14**）
